# Identification of a *cis*-regulatory element by transient analysis of co-ordinately regulated genes

**DOI:** 10.1186/1746-4811-4-17

**Published:** 2008-07-07

**Authors:** Andrew P Dare, Robert J Schaffer, Kui Lin-Wang, Andrew C Allan, Roger P Hellens

**Affiliations:** 1 The Horticultural and Food Research Institute of New Zealand, PB 92169, Auckland, 1142, New Zealand

## Abstract

**Background:**

Transcription factors (TFs) co-ordinately regulate target genes that are dispersed throughout the genome. This co-ordinate regulation is achieved, in part, through the interaction of transcription factors with conserved *cis*-regulatory motifs that are in close proximity to the target genes. While much is known about the families of transcription factors that regulate gene expression in plants, there are few well characterised *cis*-regulatory motifs.

In Arabidopsis, over-expression of the MYB transcription factor *PAP1 *(*PRODUCTION OF ANTHOCYANIN PIGMENT 1*) leads to transgenic plants with elevated anthocyanin levels due to the co-ordinated up-regulation of genes in the anthocyanin biosynthetic pathway. In addition to the anthocyanin biosynthetic genes, there are a number of un-associated genes that also change in expression level. This may be a direct or indirect consequence of the over-expression of PAP1.

**Results:**

Oligo array analysis of PAP1 over-expression Arabidopsis plants identified genes co-ordinately up-regulated in response to the elevated expression of this transcription factor. Transient assays on the promoter regions of 33 of these up-regulated genes identified eight promoter fragments that were transactivated by PAP1. Bioinformatic analysis on these promoters revealed a common *cis*-regulatory motif that we showed is required for PAP1 dependent transactivation.

**Conclusion:**

Co-ordinated gene regulation by individual transcription factors is a complex collection of both direct and indirect effects. Transient transactivation assays provide a rapid method to identify direct target genes from indirect target genes. Bioinformatic analysis of the promoters of these direct target genes is able to locate motifs that are common to this sub-set of promoters, which is impossible to identify with the larger set of direct and indirect target genes. While this type of analysis does not prove a direct interaction between protein and DNA, it does provide a tool to characterise *cis*-regulatory sequences that are necessary for transcription activation in a complex list of co-ordinately regulated genes.

## Background

DNA sequence motifs that recruit the transcription factors necessary to regulate the expression of a gene, are most commonly found in the flanking DNA regions and provide specificity to the core transcriptional machinery [1]. In plants with annotated whole genome sequence such as Arabidopsis [2], flanking DNA sequences upstream of the coding region can easily be defined. Such sequences are commonly referred to as the promoter and while they can be difficult to delineate in the absence of experimental characterisation, they can be defined as the intergenic sequence upstream of the ATG, and often limited to a defined length eg. 3 kb [3]. In this definition the promoter fragment includes the 5' untranslated region (5'UTR).

DNAse I footprinting [4] and electrophoretic or gel mobility shift assays [5] have been extensively used to characterise *cis*-regulatory elements. Both methods rely on the direct interaction between DNA fragments that contain the DNA-binding region and the corresponding transcription factor. More recently, ChIP-microarray (also known as ChIP-chip) has been used to immunoprecipitate DNA associated with a TF of interest. The DNA from this complex is then used to probe a genomic DNA microarray [6,7]. Studies which have used ChIP to identify TF binding sites include the analysis of the *AGAMOUS *[8], *AGL15*, [9] and the *FLOWERING LOCUS C PROTEIN *(*FLC*) [10] MADS box genes from Arabidopsis, all of which have been shown to bind to a CArG box contained in the promoter of the target gene.

Surface plasmon resonance (SPR) is an emerging technology that allows the characterisation of protein DNA interactions *in-vitro *[11]. Importantly, this technique allows an assessment of DNA-protein kinetics, affinity and specificity in real time. A number of plant TF binding sites have been investigated using this technique including, ZPT2-2 from petunia [12] and VRN-1 from Arabidopsis [13].

Transcription factor-DNA interactions do not however infer transcriptional activation, for example the *Antirrhinum *MYB305 protein has been shown to bind the CHS promoter in gel-shift analysis but failed to induce transcriptional activation of the gene in yeast-1-hybrid assays [14]. In addition, these experimental approaches rely on the need to purify TF protein beforehand and will only reflect *in-vitro *binding. Often these associations require co-factors or additional transcription factors that facilitate the interaction of a protein to its *cis*-regulatory regions [15,16]. Alternatively, yeast-1-hybrid assays determine protein-DNA interactions through transcriptional activation of several reporter genes: *HIS3*, *URA3 *and *LEU2 in-vivo *[17,18]. While these assays are effective at analysing simple protein-DNA interactions, the absence of any plant-derived factors other than the TF under investigation, can limit the applicability of this technique. The limited number of well characterised TF binding sites highlights the difficulty in adopting these approaches for large-scale characterisation of *cis-*regulatory sites in plants [3].

Whilst there are relatively few confirmed *cis*-regulatory sites in relation to the number of known transcription factors, a number of TF classes have consensus binding sites proposed. The bZIP class of transcription factors have been shown to preferentially bind palindromic sites such as the G-box (CACGTG) [19], A box (TACGTA) and C-box (GACGTC) [20,21]. Several plant MADS box genes including; *DEFICIENS *(*DEF*) and *GLOBOSA *(*GLO*) from *Antirrhinum *[22], *APETALA-1 *(*AP1*), *APETALA-3 *(*AP3*), *PISTILLATA *(*PI*) [23] and *AGAMOUS (AG*) [24] in Arabidopsis have been shown to bind variations of a CArG motif, and a consensus CArG sequence has been described as CC(A/T)_6_GG [25]. *LEAFY (LFY) *controls the switch from vegetative to reproductive development in Arabidopsis [26] and interacts with the consensus *LFY *binding site (CCANTG) to activate *AP1 *in the meristem identity pathway and the floral homeotic *AG *gene [27]. The WRKY TF class has been implicated in responses such as pathogen defence, senescence and trichome development [28] and bind to a conserved W box TTTGAC(C/T) motif contained in their respective target promoters [29-32]. MYB transcription factors regulate a diverse range of pathways including secondary metabolism, signal transduction and defence responses [33]. Two MYB binding site sequence variants described in plants by Romero *et al*. (1998) are the type II (GTT(A/T)GTT(G/A) and IIG G(G/T)T(A/T)GGT(G/A) sites common to a number of genes in the phenylpropanoid pathway [34]. A third conserved sequence (A/C)ACC(A/T)A(A/C)C, has been shown to be bound by the flavonoid regulator MYB305 from *Antirrhinum majus *[35].

### PAP and anthocyanin biosynthesis

*PRODUCTION OF ANTHOCYANIN PIGMENT 1 (PAP1*) is an R2–R3 MYB gene from Arabidopsis that is responsible for the co-ordinated up-regulation of genes in the anthocyanin pathway [36]. The anthocyanin biosynthetic pathway has been well characterised at both the biochemical and regulatory level. While over-expression of single enzyme components of the flavonoid pathway does not significantly alter the amount of anthocyanin in plants; over-expression of the *PAP1 *gene activates components of the biosynthetic pathway enabling increases in anthocyanin accumulation [36].

Microarray studies of transgenic Arabidopsis over-expressing *PAP1 *identified a list of 38 genes that were selected as significantly changing in expression [37]. This study examined constitutive expression effects in a mature plant so some genes with an altered expression profile may be due to indirect effects of gene over-expression, such as alteration in cell physiology or metabolite partitioning in response to the increase in anthocyanins. Or the result of other transcription factors that activate the expression of a different set of genes.

Here we describe a novel method that used transient assays to identify and validate a *cis*-regulatory motif that is necessary for transactivation by PAP1. Candidate genes were selected from microarray analysis of *PAP1 *over-expressing transgenic plants. We identified a sub-set of promoters that were directly transactivated by PAP1 and used this information to identify a sequence motif that was conserved within the promoter regions of these unrelated genes. Deletion and mutation of this candidate *cis*-regulatory element in two promoters led to significant reductions in the level of transactivation by PAP1. Taken together our results demonstrate that validation of microarray data by transactivation assays provides a powerful way of elucidating conserved motifs within co-ordinately regulated genes.

## Results and Discussion

### Selection of differentially expressed genes resulting from the over-expression of *PAP1*

**Figure 1:**
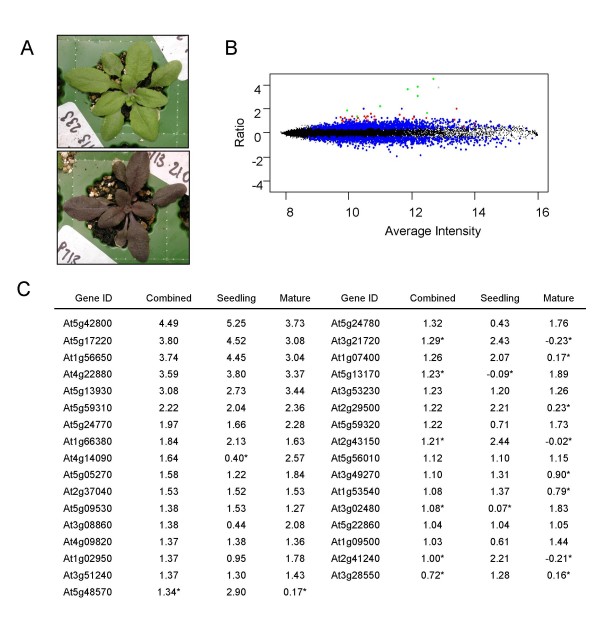
Analysis of transgenic Arabidopsis over-expressing *PAP1*. A, Plants at the 21 day stage containing vector only (top) and 35S-*PAP1 *(bottom) B, Comparison of intensity-ratio plot of gene expression in transgenic plants; Blue dot represents genes that change in expression with an adjusted *P *value <0.05, red dot represents 33 genes cloned for promoter analysis and green dot represents genes with significant transactivation when co-infiltrated with the 35S-*PAP1 *construct. The grey dot represents *PAP1 *that was over-expressed. C, Comparison of the 33 genes selected for promoter analysis. Ratios generated between wild type and *PAP1 *over-expression plants are shown. Ratios from seedlings (Seedling), mature plants (Mature) and a combined dataset of mature plants and seedlings (Combined) are given. Ratios with * are not selected.

A 35S-*PAP1 *over-expression cassette was used to generate a number of stable transgenic Arabidopsis lines with varying levels of anthocyanin accumulation. These plants showed elevated levels of anthocyanin accumulation in all plant parts when grown under standard conditions. The level of expression of the transgene correlated strongly with the levels of anthocyanin. One line homozygous for the transgene and with consistently high levels of anthocyanins was chosen for this study (Fig. 1A). Microarray analysis of labelled RNA from this *PAP1 *over-expression line was compared with transgenic lines that contained a vector-only control construct [38]. Four microarrays were hybridised with RNA from seedlings, and four microarrays were hybridised with labelled RNA from mature plants (two biological replicates repeated in a dye swap). Using a false rate discovery threshold of 0.01, 1744 genes were identified that showed significantly different expression levels, in both seedlings and mature plants, between the plants containing the 35S-*PAP1 *gene and those that contained the 35S control construct only (Additional file 1).

We do not believe that all the gene expression changes seen in both our microarray analysis and those previously published were direct targets of the *PAP1 *gene. It is likely that pleiotropic expression changes will arise from effects such as alterations in the cell physiology and downstream regulation by transcription factors. A total of 35 genes on the array list were annotated as regulatory genes, consistent with the hypothesis that many gene expression changes observed are the result of the secondary effects.

### A subset of up-regulated genes are also transactivated in leaf infiltration assays

**Figure 2:**
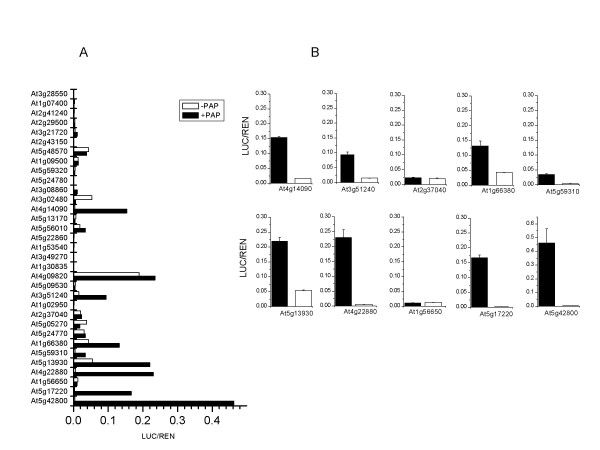
Transient assay data for promoters up-regulated in *PAP1 *over-expression transgenic plants. A, High-throughput screen of 33 promoters assayed with (black bars) or without (white bars) co-infiltration of *PAP1*. B, Re-analysis and transactivation standard error estimation for eight promoters identified in primary screen and two non-responsive control promoters (At2g37040 and At1g56650). Average transactivation values were calculated from 6 fold experimental replicates. Standard errors were calculated for error bars.

From the 1744 genes that significantly changed in transcript level due to over-expression of the *PAP1 *gene, 33 were selected for further investigation; based on those genes that showed the greatest increase in expression compared with the control vector. The gene set comprised 17 that increased only in mature plants, 7 that increased only in seedlings and 9 that changed in both tissue types. A previous study using Affymetrix Arabidopsis genome arrays, identified a subset of 39 genes that were up-regulated in response to *PAP1 *over-expression [37]. Of these 39 genes, 7 were also selected in our gene set. The 33 promoter fragments were cloned into a transactivation assay vector, pGreen 0800-LUC [38] (Fig. 1B and 1C). To simplify the cloning process and to minimise PCR induced errors we used 1 kb of upstream sequence plus the 5' UTR (where annotated) to create the promoter constructs. *Agrobacterium *containing the cloned promoter constructs were infiltrated into the leaves of *N. benthamiana *either with or without *Agrobacterium *containing the 35S-*PAP1 *cassette used to generate the transgenic plants (Fig. 2A). From this initial screening, eight promoters showed a statistically significant increase in relative luciferase (LUC) activity when co-infiltrated with the 35S-*PAP1 *cassette, compared with the promoter-only controls (Fig. 2A). While most of the promoters gave a low level of relative LUC activity in the absence of the *PAP1 *gene, the promoter for At4g09820 *TRANSPARENT TESTA 8 *(*TT8*) gave a high level of activity in the absence of *PAP1*. Interestingly, TT8 is a bHLH transcription factor that is an important co-factor in the regulation of both anthocyanins and condensed tannins [39]. The result here implies that, in our tobacco assay, the *TT8 *promoter fragment is relatively active transcriptionally.

Eight PAP1 transactivated promoters and two non-responsive promoters were re-assayed and the transactivation confirmed (Fig. 2B). Of the eight trans-activated promoters identified by our analysis, six were from genes whose encoded proteins have a role in the anthocyanin and proanthocyanin biosynthetic pathways. In addition to these, a gene corresponding to a lipid transfer protein precursor (At5g59310) and a MYB transcription factor (At1g66380) were also identified in our microarray experiment and confirmed in transactivation assays. Lipid transfer proteins are a class of small basic soluble proteins capable of binding fatty acids and acyl CoA esters [40]. As malonyl-CoA is an early precursor of the anthocyanin biosynthetic pathway [41], it is possible that lipid transfer proteins may be acting in a transport role or as co-factors for the conversion of these intermediates to anthocyanins. The transient assay data also indicate that PAP1 was able to transactivate the transcription of a second MYB like gene *MYB114 *(At1g66380). In Arabidopsis *MYB114 *belongs to a tandem repeat with two other MYB genes, *MYB113 *and *MYB90 *(*PAP2*). All these genes show significant sequence similarity to *PAP1 *(*MYB75*), although only the *PAP1 *and *PAP2 *genes have been reported to regulate anthocyanin biosynthesis. This observation supports a potential role for the *PAP1 *gene in a feed-forward regulation of at least one related MYB gene.

One promoter, for the gene encoding dihydroflavanol reductase (*DFR*; At5g42800), showed a 122-fold elevation in relative LUC activity in the transactivation assay. Three promoters from the genes encoding glutathione S-transferase (*GST*; At5g17220), leucoanthocyanidin dioxygenase (*LDOX*; At4g22880) and UDP flavonoid 5-O-glycosyltransferase (*UFGT*; At4g14090) showed a 38- to 60-fold elevation. The remaining four promoters derived from the genes encoding chalcone synthase (*CHS*; At5g13930), flavanone 3-hydroxylase (*F3H*; At3g51240), *MYB114 *(At1g66380) and a non-specific lipid transfer protein precursor (*LTP*; At5g59310), showed a PAP1-dependent elevation of 3- to 7-fold (Table 1).

**Table 1:** The PAP1 *cis*-regulatory element in eight transactivated promoters

**EST Identifier**	**Gene ** **description**	**PCE sequence**	**Fold increase ** **LUC:REN**
At5g42800	*DFR*	CCACCACGTG	121.6
		TCCCCACGTG	
At5g17220	*GST*	CCACCACATG	60
At4g22880	*LDOX*	TCTCCACGTG	37.8
At4g14090	*UFGT*	CCTCCACAAG	40.5
At5g13930	*CHS*	TCATCACATT	4.1
At5g59310	*LTPP*	CCATCACGTT	7.4
At1g66380	*MYB114*	CCGTCACGTG	3.1
At3g51240	*F3H*	CCGCCACGTG	6.3
PCE consensus sequence		YCNCCACRWK	

Conserved CCAC core is underlined. Fold increase in LUC/REN ratio over promoter only control is also shown.

### Motif analysis of transactivated promoter sequence

The naive motif search programme MEME [42], was used to search the DNA sequences for sequence motifs that were common to all eight promoters transactivated by the *PAP1 *gene. With the default settings and a maximum of five output motifs, only one 10 bp motif was present in all eight of the transactivated promoters (Table 1). This conserved motif was found in both plus and minus orientations and upstream of the 5' UTR where annotation is available. This motif was absent (*P*-value < 0.119) from the 25 promoter fragments that were initially screened and did not alter relative LUC activity in the presence of the *PAP1 *gene. From these predictions, we hypothesise that this conserved motif (C/T)CNCCAC(A/G)(A/T)(G/T) is a PAP1 *cis*-regulatory element (PCE). Searches performed on the same promoter set using the search program COSMO [43] yielded a related motif with a common core and related flanking sequences (C/T)(A/C)NCCACN(G/T)(G/T). When MEME analysis was conducted on the top 10 up-regulated genes from both this study and those previously published, neither the PCE nor any other motif was identified. This demonstrates the benefit of using only direct targets identified in the transient assay.

### Fold change and PCE frequency

The level of transactivation from the co-infiltration of 35S-*PAP1 *varied between promoter-LUC cassettes (Table 1). Notably the promoter with the highest transactivation values At5g42800 (*DFR*), which showed a 122-fold increase in luciferase activation when co-infiltrated with 35S-*PAP1*, contains two PCEs within the promoter region used in the assay. Three promoters showed between 38- to 60-fold increases in relative LUC activity (*GST*; At5g17220, *LDOX*; At4g22880 and *UFGT*; At4g14090) and contained only a single PCE. Four other promoters (*CHS*; At5g13930, *LTP*; At5g59310, *MYB114*; At1g66380 and *F3H*; At5g51240) had much smaller 3- to 7-fold increases in relative LUC activity. This lower activation may be explained by the C to T change in the first base of the highly conserved CCAC core of the PCE motif in three of the promoters with the lowest transactivation values. However, this does not explain the data from the At3g51240 promoter, which had a low transactivation value and a fully conserved PCE motif (*P*-value = 5.56e^-08^). As the levels of expression from the transiently infiltrated 35S-*PAP*1 gene were higher than under normal physiological conditions, an alternative explanation for these lower transactivation values is that high levels of *PAP1 *expression may result in non-specific binding to low affinity sites in the promoter. However, as the 35S-*PAP1 *and promoter-LUC fusion were infiltrated in a ratio of 9:1, these transient leaf infiltration assays may more closely resemble the physiological ratio of TF to promoter than the over-expression of a TF in transgenic plants.

### Validation of PCE by transient leaf infiltration

**Figure 3:**
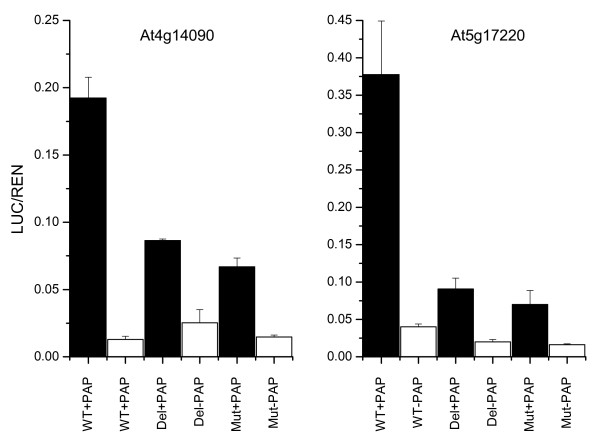
Deletion and mutation of PAP1 *cis*-regulatory elements from At5g17220 and At4g14090 promoters. Transactivation data of At5g17220 and At4g14090 promoters displaying the effect of deleting and mutating the 10 bp PCE from At5g17220 and At4g14090 promoters. Wild-type (WT) promoter, PCE deletion promoter (Del) and PCE mutation promoter (Mut) are shown and transactivation data as black bars (with PAP1), and white bars (without PAP1).

We tested the integrity of the PCE by deleting and mutating the sequence from the At5g17220 (*GST*) and At4g14090 (*UFGT*) promoter fragments. PCE promoter deletions had the 10 bp motif excised from the At5g17220 and At4g14090 promoter fragments. These were labelled At5g17220D and At4g14090D respectively. The sequence from At4g42800 (*DFR*) was not chosen for deletion or mutation analysis due to the presence of two PCEs which complicates base modification by PCR. Promoter mutations were generated by altering four bases of the PCE at positions 1 (C to T), 4 (C to T), 7 (C to T) and 10 (G to A) in the At5g17220 promoter. In promoter At4g14090, six base changes were made at positions 1 (C to T), 4 (C to T), 5 (C to A), 7 (C to T) 8 (A to T) and 10 (G to T). These modified promoters were labelled At5g17220M and At4g14090M respectively. Transactivation assays were used to compare these modified promoters with the unmodified At5g17220 and At4g14090 promoters (Fig. 3). At4g17220D had a significantly reduced LUC/REN ratio compared with the wild type sequence in the presence of 35S-*PAP1*. At4g17220M also showed a reduction in LUC/REN ratio in the presence of 35S-*PAP1*, compared with the wild type sequence. Modification of the PCE from At4g14090 showed similar reductions in relative LUC/REN ratio for At4g14090D and At4g140909M compared with the wild type. The six base changes in the PCE of At5g17220M appeared to have more of an effect than the 10 base changes introduced into the PCE region of the At4g14090 promoter. This may be due to different base substitutions at position 10, (At5g17220; G to A change, At4g14090; G to T change). A more detailed analysis of each of the conserved nucleotides within the PCE will be needed to fully interpret the significance of these results.

Interestingly, five of the PCE-containing promoters identified in this study also contained a perfect G-box site (CACGTG) adjacent to the PCE sequence. A number of plant promoters regulated by diverse signals contain G-box elements [44]. At least 2 classes of TFs are capable of binding G-boxes: the basic leucine zipper class (bZIP) and the basic helix-loop-helix proteins (bHLH) [19]. Extensive genetic and protein studies have shown a close functional relationship between MYBs regulating anthocyanin accumulation and bHLH proteins [15]. In maize, the activation of anthocyanin biosynthetic genes by ZmPl and ZmC1 requires a bHLH protein encoded by a *R/B *gene [45]. In Arabidopsis the bHLH encoding *TT8 *gene and a MYB TF encoded by the *TT2 *gene, act synergistically to direct the expression of the *DFR *and *BANYULS *(*BAN*) flavonoid pathway genes [39]. PAP1 has been demonstrated to interact with the bHLH proteins encoded by *ENHANCER OF GLABRA3 *(*EGL3*) and *GLABRA3 *(*GL3*) genes, and when co-overexpressed these combinations showed far more severe phenotypes than would be expected for additive regulation alone [46]. The presence of the PCE and the G-box may not be coincidental and may not correspond to the binding site of PAP1, but that of the bHLH gene that we assume to be necessary for transactivation. In these transient assays we presume the appropriate endogenous bHLH protein interacts with the transient expressed *PAP1 *gene product.

### Occurrence of the PCE in 300 genes from microarray results

**Figure 4:**
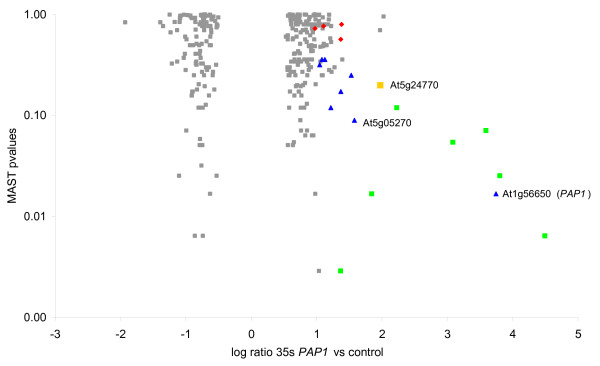
Occurrence of the PAP1 *cis*-regulatory element in the top 300 microarray selected genes. A 3 kb promoter fragment for each of the top 300 genes was analysed for the presence of PCEs using MAST. The average fold change from all microarrays was compared with the *P*-value obtained from the MAST analysis. Genes highlighted in green represent those genes that have a PCE and were transactivated by PAP1. Blue represents those that have a putative PCE but not in the first 1 kb tested. Yellow represents a gene with a PCE in the first 1 kb but was not transactivated. Red represents genes with no PCE and not transactivated. Grey represents untested genes.

We calculated probability values for putative PCEs occurring in the 2 kb upstream of the ATG of the top 300 up or down regulated genes selected from the combined mature plant and seedling microarrays, using MAST [47]. This combined analysis did not select one of the eight (At4g14090) that was transactivated in the transient assay due to it having low difference of expression in seedlings. MAST is a search program which scans input sequences for known motifs and calculates match scores for each input sequence. From the MAST output a combined *P*-value can be obtained which measures the strength of the match of the sequence to the input motif. These combined *P*-values were plotted against the log-fold change calculated from the microarray results (Fig. 4). It was found that of the 6 genes that showed the biggest up-regulation of expression in the 35S-*PAP1 *plants, all had good (<0.119) *P*-values for the PCE and were transactivated in the transient assay. When a less than log 2 fold up-regulation was observed, the occurrence of a PCE and transactivation became less easy to predict. Of the 7 tested genes that showed transactivation, the combined MAST *P*-value ranged from 2.8e^-3 ^to 0.119 suggesting that in this range, transactivation could occur. One of the 25 tested promoters (At5g24770; *VEGETATIVE STORAGE PROTEIN 2*) that was not transactivated by the *PAP1 *gene did have a putative PCE (CCATCACAAG), but only with a *P*-value of 0.199. This suggests that this level of identity to the PCE consensus was insufficient to activate the gene. One additional promoter that was not transactivated (At5g05270, *CHALCONE-FLAVANONE ISOMERASE*) contained a PCE within this *P-*value range but located outside the 1 kb region tested for transactivation. Of the top 300 genes that showed changes in expression in the presence of the *PAP1 *transgene, a further 18 untested genes were significantly up-regulated and had a PCE within the transactivation *P*-value range, suggesting that these may also be regulated by PAP1 (Fig. 4). In addition to these 18 genes, there were 13 genes (4%) that had a *P-*value within this range, but were down-regulated. This either implies a repressor function or that the presence of the PCE alone is not sufficient to cause activation of these genes.

## Conclusion

The power of bioinformatics and the availability of whole genome sequence has enabled a comprehensive description of transcription factor families in plants. There is much less known about the mechanism that these genes employ to effect co-ordinated regulation. While methods that assay the direct interaction between DNA and proteins have proved effective in the characterisation of some of these genes, this is often limited to those proteins that can be easily purified, form simple complexes or have very high affinity for the target DNA. In addition to the core binding sites that seem to be associated with transcription factor families, there may a degree of subtlety in the *cis*-regulatory elements necessary for transcription factors to facilitate their unique regulatory effects. These gene-specific *cis*-regulatory regions may function through the recruitment of TF combinations or through a DNA motif consensus that is difficult to determine using conventional methods. Here we have used transient infiltration assays to analyse several promoters from unrelated genes that have a co-ordinated up-regulation in response to the over-expression of the MYB transcription factor PAP1. Using computer-based motif searches, we were able to identify a conserved region common to all promoters that were transactivated by the *PAP1 *gene product. While it is not necessarily the PAP1 binding domain, it is a region that is necessary for PAP1 regulation and as such, this method provides an effective tool to complement DNA-protein interaction assays in the effort to elucidate *cis*-regulatory domains of transcription factors. It is also worth noting that this assay uses a heterologous system based on the expression of Arabidopsis genes in tobacco, it is therefore possible that this expression pattern may differ from the native responses in Arabidopsis.

## Materials and methods

### Plant material and growth conditions

The 35S-*PAP1 *construct was generated by inserting a genomic clone of the Arabidopsis *PAP1 *(At5g56650) gene into a nos-kanamycin containing vector pGreenII 0029-62-SK as previously described in Hellens *et al. *(2005) [38]. Constructs were electroporated into *Agrobacterium tumefaciens *GV3101 (MP90) then transformed into *Arabidopsis thaliana *col-1 plants using the floral dip method [48]. These plants, and vector-only controls, were grown together in either a greenhouse under short day conditions (8 h light/16 h dark, 21°C) or a growth room (constant light, 25°C). For the transient assays, *Nicotiana benthamiana *plants were grown, and transient leaf assays carried out as described in Hellens *et al. *(2005) [38]. The LUC/REN ratio was used to quantify promoter activity and is a measure of luciferase expression relative to the expression of 35S-*Renilla *also contained on the same reporter plasmid. Background levels of promoter activity were assessed using only the promoter-LUC-35S-REN constructs (no transcription factor) [38].

### Microarray analysis

RNA was extracted from seedling and mature Arabidopsis plants according to Chang *et al. *(1993) [49]. RNA was quantified for integrity and concentration using a 2100 BioAnalyzer (Agilent technologies). RNA was labelled with Cy 3 and Cy 5 fluorescent dyes (GE Healthcare) as previously described [50]. All analysis compared 35S-*PAP1 *plants with plants containing vector only. Each condition was repeated twice with a dye swap comparison for each repeated sample (4 arrays).

Arabidopsis full genome 27 K oligo microarrays (Operon) were spotted onto epoxy coated slides (MWG) in a 150 mM phosphate buffer, pH 8.5, using a Biorobotics MicroGrid robot and Biorobotics 100 μM pins. Microarrays were hybridised as previously described [50] except the 16-hour hybridisations were carried out at 60°C rather than 45°C. Arrays were scanned using a Genepix 4000 scanner and spots were aligned using Genepix 5 software. All data were processed in R using the Bioconductor limma package [51]. Genes were selected as significant using a False Discovery Rate (FDR) of 0.05 [52].

### Promoter cloning and plasmid constructs

Promoter sequences were defined according to TIGR 6.0 annotation of the Arabidopsis genome. A 1 kb upstream fragment and the 5'UTR, where present, was amplified by two oligonucleotide primers, one which flanked the ATG start codon and one 1 kb upstream (Additional file 2). The primers introduced *Xma *I and *Not *I restriction sites into the amplification product respectively, to facilitate directional cloning. Promoter fragments were cloned into a pGem-T easy (Promega Madison, WI) and directionally subcloned into a pGreenII-0800-LUC [38] using the *Xma*I and *Not*I restriction sites and verified by sequencing.

Motif deletions and mutations were created by designing divergent PCR primers that flanked or spanned the predicted motifs in At5g17220 and At4g14090 promoters (Additional file 3). PCR was performed on the corresponding pGem-T easy clone of the promoter fragments using Prime Star polymerase (Takara Shiga, Japan). Blunt-ended PCR products were phosphorylated with 1 mM ATP, 10U T4 Polynucleotide Kinase (New England Biolabs Ipswich MA), and 1× Polynucleotide Kinase Buffer for 1 h at 37°C then re-ligated using the Rapid DNA ligation kit (Roche Mannheim Germany) for 2 h at room temperature to recreate the vector. Modified promoters were sequence verified and directionally subcloned as above.

### Identification of PAP1 cis-regulatory elements

Conserved motifs were identified using the MEME motif search programme [42], with default variables of the following parameters: 1) Any number of repetitions of motif per sequence, 2) motif length min = 6 bp, max = 10 bp, 3) maximum of 5 motifs searched. Only motifs that were represented a least once in each promoter were considered as potential PAP1 *cis*-regulatory elements. The motif search programme COSMO [43] was also used to identify conserved motifs. Default variables were used with motif length min = 6 bp and max = 10 bp.

## Competing interests

The authors declare that they have no competing interests.

## Authors' contributions

APD for experimental design, promoter cloning, data collection, sequence analysis and manuscript preparation. RJS carried out microarray analysis and contributed to manuscript preparation. KLW for expression analysis of transgenic plants. ACA transgenic plant growth and generation. RPH contributed to experimental design and manuscript preparation.

## Supplementary Material

Additional file 1 1744 genes up-regulated on microarray of 35S-*PAP1 *Arabidopsis. Click here for file

Additional file 2 Oligonucleotide primer pairs used to PCR amplify 1 kb of Arabidopsis promoter sequence. Click here for file

Additional file 3 Oligonucleotide primer pairs used to delete or mutate the PCE in the At5g17220 and At4g14090 promoters. Click here for file
