# Supplementary material for: Identification of a cis-regulatory element by transient analysis of co-ordinately regulated genes
Source: Plant Methods. 2008 Jul 7;4:17. doi: 10.1186/1746-4811-4-17 (PMC2491621; doi:10.1186/1746-4811-4-17)
Supplement: Additional file 1 — 1744 genes up-regulated on microarray of 35S-PAP1 Arabidopsis. [file 1746-4811-4-17-S1.doc]

| Additional file 1. 1744 genes upregulated on microarray of 35S-*PAP1* Arabidopsis | | | | | | | |
| --- | --- | --- | --- | --- | --- | --- | --- |
| order | | Ratio | Average intensity | sort(p.adjusted)[1:N] | | CLONE_ID | Description |
| 1 | | **3.59** | **11.85** | **8.59E-07** | | **At4g22880** | **leucoanthocyanidin dioxygenase (anthocyanidin synthase) (LDOX/ANS), putative** |
| 2 | | **3.08** | **12.18** | **1.88E-06** | | **At5g13930** | **chalcone synthase (naringenin-chalcone synthase)** |
| 3 | | **4.49** | **12.69** | **4.22E-06** | | **At5g42800** | **dihydroflavonol 4-reductase (dihydrokaempferol 4-reductase) (DFR)** |
| 4 | | *3.74* | *12.85* | *8.85E-06* | | *At1g56650* | *Arabidopsis thaliana putative transcription factor (MYB75) mRNA, complete cds* |
| 5 | | 1.97 | 13.42 | 8.85E-06 | | At5g24770 | vegetative storage protein Vsp2 |
| 6 | | **3.80** | **12.19** | **1.14E-05** | | **At5g17220** | **glutathione transferase, putative** |
| 7 | | 2.02 | 10.48 | 4.89E-05 | | At1g03940 | transferase family |
| 8 | | 1.39 | 12.08 | 5.65E-05 | | At5g56000 | heat shock protein 81.4 (hsp81.4) |
| 9 | | -1.93 | 11.52 | 5.75E-05 | | At1g17710 | Arabidopsis thaliana chromosome I BAC F11A6 genomic sequence |
| 10 | | 1.53 | 11.62 | 6.97E-05 | | At2g37040 | phenylalanine ammonia lyase (PAL1) |
| 11 | | -1.13 | 14.97 | 7.03E-05 | | At1g23130 | Bet v I allergen family |
| 12 | | 1.37 | 10.11 | 7.05E-05 | | At4g09820 | Arabidopsis thaliana mRNA for transparent testa 8 bHLH protein, (tt8 gene) |
| 13 | | 1.12 | 12.03 | 8.66E-05 | | At5g56010 | heat shock protein, putative |
| 14 | | 1.38 | 10.61 | 8.66E-05 | | At5g09530 | proline-rich protein family |
| 15 | | 0.98 | 10.57 | 2.42E-04 | | At4g20850 | expressed protein |
| 16 | | 1.11 | 12.26 | 2.42E-04 | | At1g56070 | elongation factor -related |
| 17 | | **1.37** | **10.27** | **2.93E-04** | | **At3g51240** | **naringenin 3-dioxygenase (flavanone 3-hydroxylase) (F3H)** |
| 18 | | -0.85 | 10.56 | 3.56E-04 | | At2g43920 | thiol methyltransferase |
| 19 | | 1.21 | 9.60 | 3.56E-04 | | At5g07990 | cytochrome P450 75A5 / flavonoid 3'-monooxygenase / flavonoid 3'-hydroxylase |
| 20 | | -1.09 | 9.76 | 4.23E-04 | | At1g16960 | hypothetical protein |
| 21 | | 1.97 | 11.68 | 4.26E-04 | | At4g34710 | arginine decarboxylase SPE2 |
| 22 | | 0.97 | 10.03 | 4.30E-04 | | At1g17745 | D-3-phosphoglycerate dehydrogenase (3-PGDH) |
| 23 | | -0.75 | 10.41 | 4.59E-04 | | At1g10070 | tat-binding protein -related |
| 24 | | -0.93 | 10.82 | 5.88E-04 | | At3g60420 | expressed protein |
| 25 | | **1.84** | **9.95** | **5.88E-04** | | **At1g66380** | **Arabidopsis thaliana putative transcription factor MYB114 mRNA, complete cds** |
| 26 | | 1.17 | 9.82 | 7.43E-04 | | At1g17745 | D-3-phosphoglycerate dehydrogenase (3-PGDH) |
| 27 | | 1.10 | 12.14 | 7.43E-04 | | At5g56030 | heat shock protein 81-2 (HSP81-2) |
| 28 | | -0.86 | 10.54 | 7.43E-04 | | At3g60520 | expressed protein |
| 29 | | 1.58 | 10.71 | 7.43E-04 | | At5g05270 | chalcone-flavanone isomerase family |
| 30 | | 0.85 | 12.51 | 7.43E-04 | | At1g78920 | Arabidopsis thaliana chromosome 1 BAC F9K20 sequence, complete sequence |
| 31 | | 0.96 | 10.00 | 7.43E-04 | | At1g17745 | D-3-phosphoglycerate dehydrogenase (3-PGDH) |
| 32 | | 1.04 | 10.05 | 7.43E-04 | | At1g17745 | D-3-phosphoglycerate dehydrogenase (3-PGDH) |
| 33 | | -1.17 | 11.72 | 7.44E-04 | | At1g22690 | gibberellin-regulated protein -related |
| 34 | | 0.88 | 11.07 | 8.33E-04 | | At4g33090 | aminopeptidase- like protein |
| 35 | | -0.89 | 12.22 | 8.33E-04 | | At1g68945 | Arabidopsis thaliana unknown protein (T6L1.23) mRNA, complete cds |
| 36 | | 1.09 | 10.85 | 8.33E-04 | | At5g40450 | expressed protein |
| 37 | | -0.84 | 9.91 | 8.33E-04 | | At1g20470 | auxin-induced (indole-3-acetic acid induced) protein family |
| 38 | | 0.87 | 10.63 | 8.33E-04 | | At5g66760 | succinate dehydrogenase [ubiquinone], putative |
| 39 | | 1.08 | 10.07 | 8.33E-04 | | At1g17745 | D-3-phosphoglycerate dehydrogenase (3-PGDH) |
| 40 | | -0.74 | 12.55 | 8.33E-04 | | At5g63670 | expressed protein |
| 41 | | 1.15 | 11.49 | 8.33E-04 | | At1g53260 | hypothetical protein |
| 42 | | 0.87 | 9.95 | 8.68E-04 | | At1g17745 | D-3-phosphoglycerate dehydrogenase (3-PGDH) |
| 43 | | 1.09 | 9.93 | 8.68E-04 | | At2g27820 | prephenate dehydratase family |
| 44 | | 0.94 | 11.31 | 8.69E-04 | | At1g78900 | ATPase 70 kDa subunit -related |
| 45 | | 0.81 | 12.92 | 8.69E-04 | | At3g44150 | expressed protein |
| 46 | | -1.08 | 11.85 | 9.18E-04 | | At1g14870 | expressed protein |
| 47 | | -1.00 | 9.76 | 9.18E-04 | | At1g36640 | expressed protein |
| 48 | | 0.97 | 10.60 | 9.31E-04 | | At5g50940 | Arabidopsis thaliana genomic DNA, chromosome 5, TAC clone:K3K7 |
| 49 | | -0.77 | 13.29 | 9.43E-04 | | At2g46600 | calcium-binding protein, putative |
| 50 | | -0.94 | 11.98 | 9.62E-04 | | At3g44450 | expressed protein |
| 51 | | 0.88 | 10.00 | 9.80E-04 | | At1g17745 | D-3-phosphoglycerate dehydrogenase (3-PGDH) |
| 52 | | -0.76 | 10.87 | 9.91E-04 | | At1g50730 | expressed protein |
| 53 | | 0.87 | 10.04 | 1.02E-03 | | At1g17745 | D-3-phosphoglycerate dehydrogenase (3-PGDH) |
| 54 | | -1.03 | 10.83 | 1.08E-03 | | At1g03850 | glutaredoxin protein family |
| 55 | | -0.93 | 13.14 | 1.14E-03 | | At1g18730 | expressed protein |
| 56 | | 0.90 | 11.83 | 1.20E-03 | | At5g08690 | H+transporting ATP synthase beta chain (mitochondrial) -related |
| 57 | | -0.77 | 9.70 | 1.20E-03 | | At3g13950 | expressed protein |
| 58 | | 0.73 | 10.69 | 1.26E-03 | | At5g65110 | acyl-CoA oxidase (gb|AAC13497.1) |
| 59 | | -1.10 | 11.84 | 1.26E-03 | | At1g02450 | expressed protein |
| 60 | | -1.35 | 13.55 | 1.28E-03 | | At5g15960 | stress-induced protein KIN1 |
| 61 | | 0.82 | 11.71 | 1.31E-03 | | At1g01320 | tetratricopeptide repeat (TPR)-containing protein |
| 62 | | -0.88 | 12.44 | 1.35E-03 | | At3g51600 | nonspecific lipid transfer protein 5 (LTP 5) |
| 63 | | 1.08 | 11.91 | 1.35E-03 | | At1g21630 | calcium-binding EF-hand family protein |
| 64 | | 0.94 | 10.11 | 1.35E-03 | | At1g17745 | D-3-phosphoglycerate dehydrogenase (3-PGDH) |
| 65 | | -0.70 | 13.44 | 1.35E-03 | | At1g15120 | Arabidopsis thaliana chromosome 1 BAC F9L1 sequence |
| 66 | | -1.11 | 9.76 | 1.35E-03 | | At5g09990 | Arabidopsis thaliana genomic DNA, chromosome 5, P1 clone:MYH9 |
| 67 | | 0.86 | 10.76 | 1.35E-03 | | At3g53260 | phenylalanine ammonia-lyase (PAL2) |
| 68 | | 0.91 | 13.21 | 1.37E-03 | | At4g28300 | proline-rich protein family |
| 69 | | -0.71 | 10.25 | 1.39E-03 | | At3g11230 | expressed protein |
| 70 | | 0.75 | 10.84 | 1.43E-03 | | At3g58610 | ketol-acid reductoisomerase |
| 71 | | -0.90 | 14.84 | 1.43E-03 | | At1g31812 | Acyl CoA binding protein -related |
| 72 | | -1.39 | 13.71 | 1.48E-03 | | At4g38930 | ESTs |
| 73 | | -0.78 | 11.20 | 1.48E-03 | | At5g55620 | expressed protein |
| 74 | | -0.84 | 12.30 | 1.49E-03 | |  | ESTs |
| 75 | | 0.88 | 9.56 | 1.49E-03 | | At3g48050 | expressed protein |
| 76 | | -0.84 | 13.98 | 1.51E-03 | | At5g24165 | expressed protein |
| 77 | | 0.93 | 13.28 | 1.53E-03 | | At3g02470 | S-adenosylmethionine decarboxylase |
| 78 | | 0.91 | 11.68 | 1.55E-03 | | At5g17030 | glycosyltransferase family |
| 79 | | -0.80 | 9.65 | 1.55E-03 | | At4g27310 | CONSTANS B-box zinc finger family protein |
| 80 | | 0.88 | 10.03 | 1.60E-03 | | At1g17745 | D-3-phosphoglycerate dehydrogenase (3-PGDH) |
| 81 | | -0.86 | 11.53 | 1.60E-03 | | At2g01590 | expressed protein |
| 82 | | 0.76 | 10.25 | 1.60E-03 | | At1g48410 | EST, Moderately similar to AGO1_ARATH ARGONAUTE PROTEIN [A.thaliana] |
| 83 | | -0.92 | 9.68 | 1.70E-03 | |  | Genomic sequence for Arabidopsis thaliana BAC T6D22 from chromosome I |
| 84 | | -1.00 | 11.30 | 1.70E-03 | | At1g67920 | expressed protein |
| 85 | | 0.89 | 12.12 | 1.70E-03 | | At1g37130 | nitrate reductase 2 (NR2) |
| 86 | | -0.71 | 11.98 | 1.70E-03 | | At5g19220 | glucose-1-phosphate adenylyltransferase, large subunit 1 |
| 87 | | 1.19 | 11.74 | 1.72E-03 | | At1g16910 | hypothetical protein |
| 88 | | 1.19 | 12.92 | 1.72E-03 | | At3g29330 | hypothetical protein |
| 89 | | 0.68 | 9.90 | 1.72E-03 | | At1g17745 | D-3-phosphoglycerate dehydrogenase (3-PGDH) |
| 90 | | -1.02 | 14.30 | 1.82E-03 | | At3g47070 | expressed protein |
| 91 | | -0.84 | 11.16 | 1.82E-03 | | At5g08240 | expressed protein |
| 92 | | 1.22 | 11.49 | 1.87E-03 | | At1g30835 | predicted protein |
| 93 | | -0.81 | 10.70 | 1.87E-03 | | At5g01220 | UDP-sulfoquinovose:DAG sulfoquinovosyltransferase (sulfolipid synthase) (SQD2) |
| 94 | | 0.97 | 10.02 | 2.00E-03 | | At1g17745 | D-3-phosphoglycerate dehydrogenase (3-PGDH) |
| 95 | | -0.91 | 11.38 | 2.03E-03 | | At5g03350 | expressed protein |
| 96 | | -0.70 | 13.27 | 2.03E-03 | | At3g02730 | thioredoxin, putative |
| 97 | | -0.79 | 11.41 | 2.10E-03 | | At1g65500 | expressed protein |
| 98 | | 0.69 | 10.09 | 2.10E-03 | | At4g31480 | coatomer beta subunit (beta-coat protein) (beta-COP), putative |
| 99 | | 0.68 | 10.31 | 2.10E-03 | | At1g29350 | expressed protein |
| 100 | | 0.97 | 9.83 | 2.10E-03 | | At3g53230 | CDC48 - like protein |
| 101 | | 0.95 | 11.34 | 2.10E-03 | | At5g67330 | NRAMP metal ion transporter 4 (NRAMP4) |
| 102 | | -0.85 | 12.36 | 2.14E-03 | | At2g42220 | rhodanese-like domain protein |
| 103 | | 0.79 | 12.63 | 2.26E-03 | | At5g63100 | expressed protein |
| 104 | | -0.72 | 12.62 | 2.29E-03 | | At5g22580 | expressed protein |
| 105 | | **2.22** | **11.00** | **2.43E-03** | | **At5g59310** | **lipid transfer protein 4 (LTP 4)** |
| 106 | | -0.90 | 10.03 | 2.43E-03 | | At4g23880 | hypothetical protein |
| 107 | | -0.74 | 10.80 | 2.44E-03 | | At3g50900 | expressed protein |
| 108 | | 0.63 | 11.93 | 2.44E-03 | | At4g00360 | cytochrome p450, putative |
| 109 | | -1.09 | 12.93 | 2.44E-03 | | At3g51920 | calmodulin 9 |
| 110 | | 0.61 | 11.10 | 2.45E-03 | | At3g06860 | fatty acid multifunctional protein (AtMFP2) |
| 111 | | -0.75 | 10.95 | 2.49E-03 | | At4g23260 | expressed protein |
| 112 | | 1.02 | 12.35 | 2.49E-03 | | At4g23600 | aminotransferase family |
| 113 | | 0.97 | 13.84 | 2.52E-03 | | At1g68725 | arabinogalactan-protein, putative (AGP19) |
| 114 | | 1.04 | 10.25 | 2.52E-03 | | At5g22860 | prolylcarboxypeptidase-related protein |
| 115 | | 0.80 | 9.79 | 2.52E-03 | | At1g17745 | D-3-phosphoglycerate dehydrogenase (3-PGDH) |
| 116 | | 0.76 | 11.17 | 2.54E-03 | | At4g02710 | ESTs, Highly similar to T01078 hypothetical protein T10P11.2.2 - Arabidopsis |
| 117 | | -0.79 | 11.44 | 2.58E-03 | | At2g15050 | lipid transfer protein, putative |
| 118 | | 0.92 | 11.23 | 2.58E-03 | | At1g31814 | hypothetical protein |
| 119 | | 1.04 | 13.84 | 2.58E-03 | | At4g30370 | zinc finger (C3HC4-type RING finger) protein family |
| 120 | | 0.93 | 12.10 | 2.62E-03 | | At5g21950 | hydrolase, alpha/beta fold family |
| 121 | | 0.63 | 10.75 | 2.62E-03 | | At1g07360 | RRM-containing RNA-binding protein, putative |
| 122 | | 0.75 | 11.26 | 2.62E-03 | | At3g02910 | expressed protein |
| 123 | | 0.84 | 11.89 | 2.62E-03 | | At2g04030 | heat shock protein, putative |
| 124 | | 0.87 | 10.19 | 2.63E-03 | | At1g17745 | D-3-phosphoglycerate dehydrogenase (3-PGDH) |
| 125 | | 0.79 | 11.89 | 2.63E-03 | | At5g59740 | protein serine /threonine kinase - like protein |
| 126 | | 1.08 | 10.82 | 2.65E-03 | | At1g53540 | 17.6 kDa heat shock protein (AA 1-156) |
| 127 | | -0.80 | 11.23 | 2.66E-03 | | At5g01220 | Arabidopsis thaliana DNA chromosome 5, BAC clone F7J8 (ESSA project) |
| 128 | | -0.65 | 9.92 | 2.69E-03 | | At1g42660 | hypothetical protein |
| 129 | | 0.85 | 11.61 | 2.69E-03 | | At3g56360 | expressed protein |
| 130 | | 0.57 | 11.51 | 2.70E-03 | | At2g33340 | transducin / WD-40 repeat protein family |
| 131 | | 1.27 | 10.73 | 2.70E-03 | | At4g35800 | DNA-directed RNA polymerase (EC 2.7.7.6) II largest chain |
| 132 | | 0.63 | 12.11 | 2.76E-03 | | At5g04140 | glutamate synthase [ferredoxin] |
| 133 | | -1.14 | 12.92 | 2.76E-03 | | At5g64770 | expressed protein |
| 134 | | -0.66 | 11.35 | 2.83E-03 | | At3g09270 | glutathione transferase, putative |
| 135 | | 0.73 | 10.79 | 2.83E-03 | | At1g06670 | DEIH-box RNA/DNA helicase |
| 136 | | 1.03 | 12.87 | 2.83E-03 | | At5g52190 | expressed protein |
| 137 | | 0.95 | 11.59 | 2.83E-03 | | At3g52930 | fructose-bisphosphate aldolase, putative |
| 138 | | 0.82 | 10.86 | 2.83E-03 | | At5g49360 | glycosyl hydrolase family 3 |
| 139 | | 1.03 | 12.34 | 2.85E-03 | | At3g45140 | lipoxygenase (LOX2) |
| 140 | | 0.74 | 11.40 | 2.86E-03 | | At3g07430 | expressed protein |
| 141 | | 0.68 | 10.27 | 2.88E-03 | | At2g43820 | EST |
| 142 | | -0.76 | 11.98 | 2.88E-03 | | At2g26560 | patatin, putative |
| 143 | | 0.82 | 11.33 | 2.90E-03 | | At4g24690 | ubiquitin-associated (UBA)/PB1 domain-containing protein |
| 144 | | 0.85 | 11.52 | 2.99E-03 | | At2g36950 | heavy-metal-associated domain-containing protein |
| 145 | | -1.09 | 9.97 | 3.06E-03 | | At1g22590 | expressed protein |
| 146 | | 0.69 | 10.41 | 3.26E-03 | | At1g62660 | glycosyl hydrolase family 32 |
| 147 | | 1.06 | 13.51 | 3.26E-03 | | At4g04050 | Arabidopsis thaliana DNA chromosome 4, contig fragment No. 11 |
| 148 | | -0.79 | 13.21 | 3.27E-03 | | At4g30660 | stress responsive protein homolog |
| 149 | | 0.92 | 9.89 | 3.40E-03 | | At5g17050 | glycosyltransferase family |
| 150 | | 0.65 | 10.19 | 3.44E-03 | | At5g39050 | transferase family |
| 151 | | 0.94 | 13.31 | 3.44E-03 | | At1g05340 | expressed protein |
| 152 | | 0.60 | 11.48 | 3.46E-03 | | At1g49670 | A.thaliana mRNA for ARP protein |
| 153 | | 0.59 | 9.94 | 3.47E-03 | | At5g09520 | proline-rich protein family |
| 154 | | -0.79 | 10.80 | 3.49E-03 | | At2g28305 | expressed protein |
| 155 | | -1.24 | 10.21 | 3.49E-03 | | At2g02990 | ribonuclease, RNS1 |
| 156 | | 0.71 | 10.50 | 3.50E-03 | | At3g56200 | amino acid transporter family |
| 157 | | -0.60 | 8.77 | 3.70E-03 | | At5g08030 | glycerophosphodiester phosphodiesterase - like protein |
| 158 | | -0.83 | 11.80 | 3.70E-03 | | At2g03440 | expressed protein |
| 159 | | -0.86 | 11.81 | 3.70E-03 | | At5g11970 | expressed protein |
| 160 | | -0.64 | 13.74 | 3.70E-03 | | At3g62030 | peptidylprolyl isomerase ROC4 |
| 161 | | 0.78 | 10.32 | 3.72E-03 | | At4g02940 | oxidoreductase, 2OG-Fe(II) oxygenase family |
| 162 | | 0.70 | 10.11 | 3.76E-03 | | At5g37510 | NADH dehydrogenase (ubiquinone), mitochondrial, putative |
| 163 | | 0.73 | 11.72 | 3.84E-03 | | At5g45430 | protein kinase, putative |
| 164 | | 0.60 | 10.37 | 3.84E-03 | | At2g22720 | expressed protein |
| 165 | | 0.74 | 12.25 | 3.92E-03 | | At5g48300 | glucose-1-phosphate adenylyltransferase, small subunit, chloroplast |
| 166 | | 0.66 | 10.21 | 3.94E-03 | | At3g63180 | expressed protein |
| 167 | | 0.56 | 13.79 | 3.97E-03 | | At2g41420 | proline-rich protein family |
| 168 | | 0.74 | 10.30 | 3.97E-03 | | At4g03580 | hypothetical protein |
| 169 | | 0.93 | 13.14 | 3.97E-03 | | At5g17670 | expressed protein |
| 170 | | -0.69 | 11.13 | 3.99E-03 | | At3g45050 | expressed protein |
| 171 | | 0.89 | 11.51 | 4.07E-03 | | At4g32375 | polygalacturonase -related |
| 172 | | 0.71 | 13.36 | 4.07E-03 | | At2g43970 | VirF-interacting protein FIP1 |
| 173 | | 0.59 | 13.36 | 4.11E-03 | | At2g36460 | fructose-bisphosphate aldolase, putative |
| 174 | | -0.54 | 13.78 | 4.20E-03 | | At2g20260 | photosystem I reaction center subunit IV -related |
| 175 | | 0.84 | 12.90 | 4.33E-03 | | At1g56410 | heat shock protein hsp70t-1 |
| 176 | | 1.10 | 13.36 | 4.33E-03 | | At3g49270 | expressed protein |
| 177 | | -0.91 | 11.67 | 4.33E-03 | | At1g11530 | thioredoxin family |
| 178 | | -0.76 | 13.33 | 4.45E-03 | | At2g24040 | expressed protein |
| 179 | | -1.25 | 10.79 | 4.45E-03 | | At1g08310 | esterase/lipase/thioesterase family |
| 180 | | -0.80 | 10.03 | 4.61E-03 | | At1g52200 | expressed protein |
| 181 | | 0.84 | 9.99 | 4.67E-03 | | At1g17745 | D-3-phosphoglycerate dehydrogenase (3-PGDH) |
| 182 | | 0.76 | 10.41 | 4.74E-03 | | At4g10150 | Arabidopsis thaliana DNA chromosome 4, BAC clone F28M11 (ESSA project) |
| 183 | | -0.85 | 12.83 | 4.86E-03 | | At4g30650 | low temperature and salt responsive protein homolog |
| 184 | | 1.13 | 12.73 | 4.88E-03 | | At5g51680 | proline-rich protein family |
| 185 | | -0.98 | 13.28 | 4.88E-03 | | At4g07750 | transposon protein -related |
| 186 | | -0.84 | 14.69 | 4.90E-03 | | At4g34620 | ribosomal protein S16p family |
| 187 | | 0.66 | 10.77 | 4.90E-03 | | At2g33770 | ubiquitin-conjugating enzyme family |
| 188 | | 0.64 | 10.07 | 4.90E-03 | | At3g13300 | transducin / WD-40 repeat protein family |
| 189 | | -0.63 | 11.21 | 5.14E-03 | | At2g26560 | patatin, putative |
| 190 | | 0.67 | 12.33 | 5.16E-03 | | At1g15950 | cinnamoyl-CoA reductase, putative |
| 191 | | -0.69 | 12.86 | 5.16E-03 | | At5g45010 | expressed protein |
| 192 | | 1.02 | 13.00 | 5.16E-03 | | At1g77180 | EST, Moderately similar to T00448 hypothetical protein T14N5.5 - Arabidopsis thaliana [A.thaliana] |
| 193 | | 0.71 | 11.63 | 5.16E-03 | | At1g70090 | glycosyltransferase family 8 |
| 194 | | 1.22 | 11.76 | 5.16E-03 | | At5g59320 | lipid transfer protein 3 (LTP 3) |
| 195 | | 1.09 | 13.31 | 5.28E-03 | | At5g09750 | bHLH protein family |
| 196 | | 0.85 | 12.26 | 5.28E-03 | | At1g09070 | C2 domain-containing protein |
| 197 | | 1.22 | 11.05 | 5.28E-03 | | At1g36160 | Arabidopsis thaliana mRNA for acetyl-CoA carboxylase, complete cds |
| 198 | | -0.61 | 11.31 | 5.28E-03 | | At4g00880 | auxin-induced (indole-3-acetic acid induced) protein family |
| 199 | | 0.75 | 11.07 | 5.28E-03 | | At5g57990 | ubiquitin-specific protease 23 (UBP23), putative |
| 200 | | 0.68 | 10.90 | 5.29E-03 | | At5g50010 | expressed protein |
| 201 | | -0.72 | 10.76 | 5.29E-03 | | At3g19660 | expressed protein |
| 202 | | 1.23 | 11.22 | 5.29E-03 | | At1g44800 | nodulin MtN21 family protein |
| 203 | | 0.73 | 9.90 | 5.42E-03 | | At2g34910 | expressed protein |
| 204 | | -0.74 | 9.71 | 5.43E-03 | | At1g03020 | glutaredoxin protein family |
| 205 | | 0.70 | 11.78 | 5.43E-03 | | At5g39650 | expressed protein |
| 206 | | -0.74 | 11.65 | 5.46E-03 | | At5g12940 | leucine rich repeat protein family |
| 207 | | 0.88 | 13.72 | 5.48E-03 | | At5g07510 | glycine-rich protein GRP14 |
| 208 | | 0.98 | 10.84 | 5.59E-03 | | At1g17020 | oxidoreductase, 2OG-Fe(II) oxygenase family |
| 209 | | 0.64 | 10.71 | 5.59E-03 | | At5g19440 | cinnamyl-alcohol dehydrogenase (CAD), putative |
| 210 | | -0.62 | 13.61 | 5.59E-03 | | At5g52750 | heavy-metal-associated domain-containing protein |
| 211 | | -0.55 | 13.53 | 5.60E-03 | | At2g02130 | Arabidopsis thaliana chromosome II section 8 of 255 of the complete sequence. |
| 212 | | 0.61 | 9.97 | 5.60E-03 | | At5g64430 | octicosapeptide/Phox/Bem1p (PB1) domain-containing protein |
| 213 | | 0.73 | 12.00 | 5.71E-03 | | At5g49910 | heat shock protein cpHsc70-2 (hsc70-7) |
| 214 | | 0.58 | 12.46 | 5.71E-03 | | At4g36130 | 60S ribosomal protein L8 (RPL8C) |
| 215 | | 1.19 | 12.98 | 5.71E-03 | | At3g12580 | heat shock protein hsp70 |
| 216 | | -0.77 | 9.72 | 5.71E-03 | | At1g16730 | hypothetical protein |
| 217 | | -0.58 | 9.20 | 5.71E-03 | | At5g55570 | hypothetical protein |
| 218 | | 1.37 | 10.49 | 5.87E-03 | | At1g02950 | glutathione transferase, putative |
| 219 | | 0.83 | 11.79 | 5.89E-03 | | At2g23350 | polyadenylate-binding protein (PABP), putative |
| 220 | | 0.77 | 10.81 | 5.89E-03 | | At4g01290 | expressed protein |
| 221 | | 0.64 | 10.26 | 5.95E-03 | | At4g03320 | chloroplast protein import component -related |
| 222 | | -0.65 | 11.11 | 6.05E-03 | | At1g48330 | expressed protein |
| 223 | | 0.72 | 12.25 | 6.05E-03 | | At2g45050 | GATA zinc finger protein |
| 224 | | -0.57 | 12.44 | 6.05E-03 | | At4g28660 | photosystem II protein W - like |
| 225 | | 0.76 | 12.57 | 6.17E-03 | | At5g10430 | arabinogalactan-protein (AGP4) |
| 226 | | -0.78 | 10.46 | 6.17E-03 | | At5g43580 | hypothetical protein |
| 227 | | 0.83 | 10.25 | 6.17E-03 | | At3g57520 | glycosyl hydrolase family 36 |
| 228 | | 0.81 | 10.02 | 6.17E-03 | | At1g17745 | D-3-phosphoglycerate dehydrogenase (3-PGDH) |
| 229 | | 0.65 | 11.01 | 6.22E-03 | | At4g16280 | Flowering time control protein (FCA) |
| 230 | | 0.58 | 11.95 | 6.22E-03 | | At2g22470 | arabinogalactan-protein (AGP2) |
| 231 | | 0.98 | 11.84 | 6.22E-03 | | At1g12040 | leucine-rich repeat extensin family |
| 232 | | -0.65 | 10.49 | 6.22E-03 | | At1g13820 | hydrolase, alpha/beta fold family |
| 233 | | 0.53 | 10.54 | 6.22E-03 | | At1g07030 | ESTs, Moderately similar to T00582 hypothetical protein T27E13.10 - Arabidopsis thaliana [A.thaliana] |
| 234 | | -0.62 | 12.00 | 6.22E-03 | | At4g30620 | expressed protein |
|  | 235 | | -0.76 | 12.32 | 6.22E-03 | At5g02120 | one helix protein (OHP) |
|  | 236 | | -0.72 | 9.42 | 6.22E-03 | At5g36920 | hypothetical protein |
|  | 237 | | 0.60 | 10.29 | 6.22E-03 | At3g21020 | Arabidopsis thaliana genomic DNA, chromosome 3, P1 clone: MSA6 |
|  | 238 | | 0.61 | 11.07 | 6.22E-03 | At4g13090 | xyloglucan endotransglycosylase, putative |
|  | 239 | | 0.84 | 11.12 | 6.22E-03 | At5g01820 | CBL-interacting protein kinase 14 |
|  | 240 | | 0.74 | 10.46 | 6.23E-03 | At4g34110 | polyadenylate-binding protein 2 (PABP2) |
|  | 241 | | -0.59 | 10.07 | 6.28E-03 | At2g33810 | squamosa-promoter binding protein -related |
|  | 242 | | -0.72 | 10.95 | 6.30E-03 | At2g22080 | En/Spm-related transposon protein |
|  | 243 | | 0.69 | 10.66 | 6.30E-03 | At1g77760 | nitrate reductase 1 (NR1) |
|  | 244 | | 0.96 | 10.95 | 6.30E-03 | At4g16370 | isp4 like protein |
|  | 245 | | 0.56 | 10.51 | 6.42E-03 | At3g57610 | adenylosuccinate synthetase |
|  | 246 | | -0.63 | 10.13 | 6.42E-03 | At1g73630 | calcium-binding protein, putative |
|  | 247 | | 0.56 | 9.76 | 6.42E-03 | At5g15400 | ubiquitin-fusion degradation protein-related |
|  | 248 | | -0.99 | 12.61 | 6.43E-03 | At2g40880 | cysteine proteinase inhibitor B (cystatin B) -related |
|  | 249 | | -1.22 | 11.87 | 6.55E-03 | At1g73010 | expressed protein |
|  | 250 | | -0.73 | 9.86 | 6.55E-03 | At3g52720 | carbonic anhydrase (CAH1) |
|  | 251 | | 0.73 | 11.69 | 6.56E-03 | At5g37520 | hypothetical protein |
|  | 252 | | 0.76 | 11.70 | 6.60E-03 | At1g15060 | expressed protein |
|  | 253 | | 0.86 | 10.13 | 6.60E-03 | At3g02260 | expressed protein |
|  | 254 | | 0.75 | 10.86 | 6.60E-03 | At5g13390 | expressed protein |
|  | 255 | | -0.73 | 10.72 | 6.60E-03 | At5g18150 | expressed protein |
|  | 256 | | 0.88 | 13.07 | 6.60E-03 | At2g33520 | expressed protein |
|  | 257 | | -0.63 | 11.63 | 6.60E-03 | At2g26240 | expressed protein |
|  | 258 | | 0.63 | 13.32 | 6.60E-03 | At3g23990 | chaperonin (CPN60/HSP60) |
|  | 259 | | -0.65 | 12.38 | 6.61E-03 | At1g21190 | expressed protein |
|  | 260 | | -0.59 | 11.01 | 6.61E-03 | At4g20780 | calcium-binding protein, putative |
|  | 261 | | 0.56 | 9.88 | 6.61E-03 | At3g15530 | expressed protein |
|  | 262 | | 0.55 | 13.29 | 6.62E-03 | At2g25490 | F-box protein family, AtFBL6 |
|  | 263 | | 0.64 | 12.69 | 6.62E-03 | At3g59660 | C2 domain/GRAM domain-containing protein |
|  | 264 | | -0.63 | 10.74 | 6.63E-03 | At3g13910 | expressed protein |
|  | 265 | | -1.01 | 13.96 | 6.64E-03 | At5g15970 | cold-regulated protein COR6.6 (stress-induced protein KIN2) |
|  | 266 | | -0.75 | 11.17 | 6.67E-03 | At1g23140 | C2 domain-containing protein |
|  | 267 | | -0.52 | 10.81 | 6.67E-03 | At2g47320 | peptidyl-prolyl cis-trans isomerase -related |
|  | 268 | | -0.53 | 12.69 | 6.67E-03 | At4g13500 | expressed protein |
|  | 269 | | 0.63 | 10.06 | 6.73E-03 | At4g25340 | immunophilin / FKBP-type peptidyl-prolyl cis-trans isomerase -related |
|  | 270 | | 0.81 | 12.46 | 6.75E-03 | At4g35480 | RING-H2 finger protein RHA3b |
|  | 271 | | 0.67 | 11.91 | 6.75E-03 | At4g33610 | Genomic sequence for Arabidopsis thaliana BAC F14J16 from chromosome I |
|  | 272 | | -0.63 | 10.82 | 6.77E-03 | At2g18200 | hypothetical protein |
|  | 273 | | -1.03 | 11.38 | 6.78E-03 | At5g18030 | auxin-induced (indole-3-acetic acid induced) protein, putative |
|  | 274 | | -0.91 | 11.67 | 6.80E-03 | At3g54100 | expressed protein |
|  | 275 | | 0.75 | 11.37 | 6.80E-03 | At5g07690 | myb family transcription factor |
|  | 276 | | 0.94 | 11.14 | 6.80E-03 | At1g48090 | predicted protein |
|  | 277 | | -0.72 | 11.41 | 6.85E-03 | At3g61190 | BON1-associated protein 1 (BAP1) |
|  | 278 | | 0.85 | 9.87 | 6.85E-03 | At5g12860 | oxoglutarate/malate translocator, putative |
|  | 279 | | 0.67 | 11.93 | 6.91E-03 | At1g51680 | 4-coumarate:CoA ligase 1 (4-coumaroyl-CoA synthase 1) (4CL1), putative |
|  | 280 | | 0.71 | 13.15 | 6.94E-03 | At5g60120 | AP2 domain transcription factor, putative |
|  | 281 | | 0.55 | 9.98 | 6.95E-03 | At2g47960 | expressed protein |
|  | 282 | | -0.61 | 10.94 | 7.04E-03 | At3g48100 | response regulator 2 (ATRR2) |
|  | 283 | | -0.53 | 10.45 | 7.06E-03 | At1g28250 | expressed protein |
|  | 284 | | 0.87 | 12.00 | 7.06E-03 | At5g14800 | pyrroline-5-carboxylate reductase |
|  | 285 | | -0.53 | 10.48 | 7.06E-03 | At2g35660 | monooxygenase family |
|  | 286 | | 1.06 | 13.13 | 7.16E-03 | At4g19670 | expressed protein |
|  | 287 | | -0.69 | 11.29 | 7.24E-03 | At5g08050 | expressed protein |
|  | 288 | | -0.62 | 12.23 | 7.24E-03 | At5g54145 | expressed protein |
|  | 289 | | 0.65 | 11.51 | 7.24E-03 | At5g21105 | L-ascorbate oxidase, putative |
|  | 290 | | 0.98 | 10.36 | 7.30E-03 | At1g32450 | peptide transporter PTR2-B -related |
|  | 291 | | -0.68 | 11.68 | 7.30E-03 | At1g63900 | zinc finger (C3HC4-type RING finger) protein family |
|  | 292 | | 0.59 | 12.83 | 7.30E-03 | At2g33150 | acetyl-CoA C-acyltransferase (3-ketoacyl-CoA thiolase), putative |
|  | 293 | | 0.59 | 10.35 | 7.30E-03 | At4g02040 | hypothetical protein |
|  | 294 | | 0.58 | 11.57 | 7.30E-03 | At1g03475 | coproporphyrinogen III oxidase (coproporphyrinogenase) (coprogen oxidase), putative |
|  | 295 | | -0.71 | 12.37 | 7.37E-03 | At5g08770 | Arabidopsis thaliana DNA chromosome 5, BAC clone T2K12 (ESSA project) |
|  | 296 | | 0.59 | 9.53 | 7.40E-03 | At2g34810 | FAD-linked oxidoreductase family |
|  | 297 | | -0.51 | 9.14 | 7.41E-03 | At1g54380 | expressed protein |
|  | 298 | | 0.65 | 10.74 | 7.41E-03 | At5g23575 | expressed protein |
|  | 299 | | 0.85 | 11.12 | 7.41E-03 |  | Arabidopsis thaliana farnesylated protein ATFP1 mRNA, partial cds |
|  | 300 | | 0.53 | 10.83 | 7.41E-03 | At3g27570 | sucrose cleavage protein -related |
|  | 301 | | -0.74 | 11.56 | 7.47E-03 |  | Arabidopsis thaliana genomic DNA, chromosome 5, TAC clone:K12B20 |
|  | 302 | | -0.76 | 12.15 | 7.47E-03 | At1g21065 | expressed protein |
|  | 303 | | 0.64 | 11.65 | 7.52E-03 | At2g14600 | hypothetical protein |
|  | 304 | | 0.71 | 11.70 | 7.58E-03 | At3g56150 | PROBABLE EUKARYOTIC TRANSLATION INITIATION FACTOR 3 SUBUNIT 8 |
|  | 305 | | -0.77 | 12.35 | 7.61E-03 | At3g27690 | light harvesting chlorophyll A/B binding protein, putative |
|  | 306 | | -0.51 | 12.95 | 7.66E-03 | At1g21770 | Arabidopsis thaliana unknown protein (F8K7.21) mRNA, complete cds |
|  | 307 | | 0.67 | 12.05 | 7.70E-03 | At5g52570 | beta-carotene hydroxylase, putative |
|  | 308 | | 0.51 | 13.31 | 7.73E-03 | At4g09000 | 14-3-3 protein GF14 chi (grf1) |
|  | 309 | | 0.70 | 11.45 | 7.83E-03 | At5g57660 | CONSTANS B-box zinc finger family protein |
|  | 310 | | 0.85 | 9.88 | 7.83E-03 | At1g54570 | esterase/lipase/thioesterase family |
|  | 311 | | 0.45 | 11.01 | 8.00E-03 | At3g02360 | 6-phosphogluconate dehydrogenase -related |
|  | 312 | | 0.61 | 11.24 | 8.12E-03 | At2g21900 | Arabidopsis thaliana chromosome II section 124 of 255 of the complete sequence. |
|  | 313 | | -0.82 | 11.74 | 8.25E-03 | At1g24145 | expressed protein |
|  | 314 | | -0.70 | 11.56 | 8.25E-03 | At2g27290 | expressed protein |
|  | 315 | | -0.52 | 12.36 | 8.30E-03 | At2g16710 | HesB protein -related |
|  | 316 | | -0.57 | 12.34 | 8.30E-03 | At3g21640 | peptidylprolyl isomerase, putative |
|  | 317 | | 0.64 | 9.27 | 8.30E-03 | At3g08850 | transducin / WD-40 repeat protein family |
|  | 318 | | -0.57 | 10.74 | 8.30E-03 | At4g24930 | thylakoid lumenal 17.9 kDa protein, chloroplast precursor |
|  | 319 | | -0.88 | 13.22 | 8.30E-03 | At4g10300 | expressed protein |
|  | 320 | | 0.69 | 11.60 | 8.30E-03 | At5g49910 | heat shock protein cpHsc70-2 (hsc70-7) |
|  | 321 | | 0.62 | 11.26 | 8.30E-03 | At3g06350 | dehydroquinate dehydratase/shikimate dehydrogenase, putative |
|  | 322 | | -0.58 | 12.42 | 8.30E-03 | At4g24990 | ubiquitin family |
|  | 323 | | 0.51 | 10.63 | 8.30E-03 | At2g35860 | expressed protein |
|  | 324 | | 0.47 | 9.83 | 8.38E-03 | At1g65620 | lateral organ boundaries (LOB) domain protein 6 (LBD6) |
|  | 325 | | -0.69 | 11.03 | 8.38E-03 | At1g26470 | expressed protein |
|  | 326 | | 0.77 | 12.38 | 8.38E-03 | At1g62700 | hypothetical protein |
|  | 327 | | 0.68 | 10.48 | 8.39E-03 | At4g36760 | aminopeptidase-related protein |
|  | 328 | | 0.77 | 11.08 | 8.39E-03 | At2g31380 | salt tolerance-like protein |
|  | 329 | | 0.80 | 11.94 | 8.40E-03 | At5g26760 | expressed protein |
|  | 330 | | 0.58 | 11.35 | 8.52E-03 | At5g20280 | sucrose-phosphate synthase, putative |
|  | 331 | | -1.14 | 10.66 | 8.52E-03 | At1g55010 | plant defensin protein, putative (PDF1.5) |
|  | 332 | | 0.85 | 10.68 | 8.59E-03 | At3g13672 | Arabidopsis thaliana genomic DNA, chromosome 3, P1 clone:MMM17 |
|  | 333 | | 1.32 | 12.06 | 8.59E-03 | At5g24780 | vegetative storage protein Vsp1 |
|  | 334 | | 1.15 | 11.56 | 8.59E-03 | At5g48880 | acetyl-CoA C-acyltransferase 1 (3-ketoacyl-CoA thiolase 1) |
|  | 335 | | -0.55 | 9.97 | 8.67E-03 | At2g21120 | expressed protein |
|  | 336 | | 0.59 | 10.27 | 8.67E-03 | At2g47440 | DnaJ domain-containing protein |
|  | 337 | | -1.11 | 10.22 | 8.67E-03 | At3g47480 | calcium-binding EF-hand family protein |
|  | 338 | | 0.52 | 10.42 | 8.67E-03 | At2g42890 | Meiosis protein mei2, putative |
|  | 339 | | -0.91 | 15.41 | 8.69E-03 | At3g15353 | expressed protein |
|  | 340 | | -0.53 | 11.91 | 8.83E-03 | At1g75690 | expressed protein |
|  | 341 | | -0.62 | 11.86 | 8.87E-03 | At1g61780 | postsynaptic protein CRIPT -related |
|  | 342 | | 0.67 | 10.83 | 8.91E-03 | At1g04780 | expressed protein |
|  | 343 | | 0.65 | 9.51 | 8.93E-03 | At5g17860 | cation exchanger, putative (CAX7) |
|  | 344 | | -1.02 | 11.34 | 9.03E-03 | At5g18020 | auxin-induced (indole-3-acetic acid induced) protein, putative |
|  | 345 | | 1.03 | 10.62 | 9.21E-03 | At1g09500 | cinnamyl-alcohol dehydrogenase (CAD) family |
|  | 346 | | -0.68 | 11.21 | 9.31E-03 | At3g20510 | expressed protein |
|  | 347 | | 0.59 | 12.45 | 9.35E-03 | At4g14960 | tubulin alpha-6 chain (TUA6) |
|  | 348 | | 0.71 | 9.31 | 9.36E-03 | At3g17900 | expressed protein |
|  | 349 | | 0.66 | 9.96 | 9.36E-03 | At5g19820 | expressed protein |
|  | 350 | | 0.47 | 13.20 | 9.36E-03 | At4g34670 | 40S ribosomal protein S3A (RPS3aB) |
|  | 351 | | -0.59 | 10.60 | 9.36E-03 | At2g46650 | cytochrome b5, putative |
|  | 352 | | -0.52 | 9.72 | 9.36E-03 | At1g78170 | expressed protein |
|  | 353 | | 0.54 | 10.02 | 9.36E-03 | At5g60580 | zinc finger (C3HC4-type RING finger) protein family |
|  | 354 | | -0.76 | 10.53 | 9.41E-03 | At2g43520 | trypsin inhibitor -related |
|  | 355 | | 0.86 | 13.47 | 9.46E-03 | At5g05090 | myb family transcription factor |
|  | 356 | | -0.64 | 10.55 | 9.47E-03 | At5g36925 | expressed protein |
|  | 357 | | -0.75 | 9.26 | 9.59E-03 | At2g46880 | calcineurin-like phosphoesterase family |
|  | 358 | | 1.03 | 11.56 | 9.62E-03 | At3g22380 | expressed protein |
|  | 359 | | 0.64 | 9.91 | 9.73E-03 | At1g45020 | Genomic sequence for Arabidopsis thaliana BAC F27F5 from chromosome I |
|  | 360 | | 0.76 | 11.74 | 9.73E-03 | At5g49910 | heat shock protein cpHsc70-2 (hsc70-7) |
|  | 361 | | -0.53 | 11.09 | 9.73E-03 | At1g05070 | expressed protein |
|  | 362 | | -0.59 | 10.43 | 9.73E-03 | At1g66890 | expressed protein |
|  | 363 | | -0.72 | 13.74 | 9.73E-03 | At4g16410 | expressed protein |
|  | 364 | | -0.77 | 14.09 | 9.73E-03 | At2g14560 | expressed protein |
|  | 365 | | -0.46 | 10.86 | 9.73E-03 | At3g56050 | putative protein kinase |
|  | 366 | | -0.83 | 12.00 | 9.73E-03 |  | ESTs |
|  | 367 | | 0.61 | 10.99 | 9.73E-03 | At4g21880 | pentatricopeptide (PPR) repeat-containing protein |
|  | 368 | | 0.47 | 12.63 | 9.76E-03 | At1g50480 | formate-tetrahydrofolate ligase (10-formyltetrahydrofolate synthetase) |
|  | 369 | | -0.95 | 12.02 | 9.76E-03 | At5g52760 | heavy-metal-associated domain-containing protein |
|  | 370 | | -0.73 | 11.33 | 9.78E-03 | At5g45630 | hypothetical protein |
|  | 371 | | 0.59 | 9.00 | 9.79E-03 | At4g04750 | sugar transporter family |
|  | 372 | | 0.91 | 10.64 | 9.79E-03 | At1g70320 | ubiquitin-protein ligase 2 (UPL2), putative |
|  | 373 | | 0.51 | 10.88 | 9.79E-03 | At5g19780 | tubulin alpha-3/alpha-5 chain (TUA5) |
|  | 374 | | -0.74 | 9.85 | 9.79E-03 | At3g29240 | chloroplast lumen common protein family |
|  | 375 | | -0.63 | 11.72 | 9.79E-03 | At1g69750 | hypothetical protein |
|  | 376 | | -0.55 | 12.66 | 9.79E-03 | At5g04750 | F1F0-ATPase inhibitor - like protein |
|  | 377 | | -0.56 | 9.23 | 9.79E-03 | At3g06920 | pentatricopeptide (PPR) repeat-containing protein |
|  | 378 | | -0.66 | 13.52 | 9.79E-03 | At4g14320 | 60S ribosomal protein L36a/L44 (RPL36aB) |
|  | 379 | | 1.12 | 11.82 | 9.79E-03 | At5g09330 | expressed protein |
|  | 380 | | 0.55 | 9.86 | 9.79E-03 | At1g05660 | polygalacturonase, putative |
|  | 381 | | 0.55 | 10.46 | 9.79E-03 | At4g16690 | esterase/lipase/thioesterase family |
|  | 382 | | 0.53 | 9.26 | 9.79E-03 | At5g50940 | expressed gene |
|  | 383 | | 0.57 | 14.19 | 9.79E-03 | At4g37450 | arabinogalactan-protein (AGP18) |
|  | 384 | | -0.67 | 11.48 | 9.79E-03 | At1g22270 | expressed protein |
|  | 385 | | 0.53 | 10.81 | 9.84E-03 | At1g23190 | Arabidopsis thaliana mRNA for cytosolic phosphoglucomutase (PGMc gene) |
|  | 386 | | -0.50 | 11.60 | 9.84E-03 | At4g26860 | Proline synthetase associated protein -related |
|  | 387 | | 0.57 | 9.64 | 9.84E-03 | At3g56600 | phosphatidylinositol 3- and 4-kinase family |
|  | 388 | | -0.55 | 10.90 | 9.87E-03 | At2g42190 | expressed protein |
|  | 389 | | 0.58 | 9.99 | 9.91E-03 | At5g55920 | nucleolar protein-related |
|  | 390 | | -0.64 | 10.57 | 9.91E-03 | At1g47813 | hypothetical protein |
|  | 391 | | -0.55 | 10.35 | 9.94E-03 | At2g30170 | expressed protein |
|  | 392 | | -0.69 | 11.16 | 9.95E-03 | At4g16000 | expressed protein |
|  | 393 | | -0.64 | 10.81 | 9.95E-03 | At4g13220 | expressed protein |
|  | 394 | | -0.70 | 13.88 | 9.95E-03 | At1g32470 | glycine cleavage system H protein precursor -related |
|  | 395 | | -0.60 | 13.12 | 1.02E-02 | At5g01750 | expressed protein |
|  | 396 | | 0.53 | 10.04 | 1.02E-02 | At3g01010 | UDP-glucose 6-dehydrogenase -related |
|  | 397 | | 0.77 | 10.99 | 1.02E-02 | At1g23205 | pectinesterase - related |
|  | 398 | | 0.86 | 12.92 | 1.02E-02 | At4g21550 | transcriptional factor B3 family |
|  | 399 | | -0.56 | 11.09 | 1.03E-02 | At3g23570 | expressed protein |
|  | 400 | | -1.23 | 12.01 | 1.03E-02 | At1g14880 | expressed protein |
|  | 401 | | 0.53 | 10.61 | 1.03E-02 | At3g54000 | ESTs, Weakly similar to T45940 hypothetical protein F5K20.300 - Arabidopsis thaliana [A.thaliana] |
|  | 402 | | -0.52 | 11.61 | 1.03E-02 | At1g28150 | expressed protein |
|  | 403 | | -0.68 | 14.08 | 1.03E-02 | At1g64370 | expressed protein |
|  | 404 | | 0.54 | 9.62 | 1.03E-02 | At1g12200 | flavin-containing monooxygenase (FMO) family |
|  | 405 | | -0.49 | 10.17 | 1.03E-02 | At1g55940 | cytochrome P450, putative |
|  | 406 | | 0.50 | 9.55 | 1.04E-02 | At3g02210 | predicted GPI-anchored protein |
|  | 407 | | 0.64 | 10.01 | 1.04E-02 | At5g64600 | auxin-independent growth promoter-related protein |
|  | 408 | | -0.57 | 9.92 | 1.04E-02 | At3g59880 | hypothetical protein |
|  | 409 | | 0.94 | 10.47 | 1.04E-02 | At1g12000 | pyrophosphate-fructose-6-phosphate 1-phosphotransferase -related |
|  | 410 | | 0.60 | 10.35 | 1.04E-02 | At1g75300 | isoflavone reductase, putative |
|  | 411 | | -0.66 | 10.66 | 1.04E-02 | At2g29460 | glutathione transferase, putative |
|  | 412 | | 0.77 | 12.17 | 1.05E-02 | At3g04510 | hypothetical protein |
|  | 413 | | 0.65 | 9.84 | 1.05E-02 | At1g35880 | hypothetical protein |
|  | 414 | | 0.54 | 10.35 | 1.05E-02 | At1g20980 | SPL1-related protein -related |
|  | 415 | | 0.59 | 10.42 | 1.06E-02 | At5g39980 | pentatricopeptide (PPR) repeat-containing protein |
|  | 416 | | 0.45 | 10.74 | 1.07E-02 | At5g42860 | expressed protein |
|  | 417 | | 0.51 | 9.51 | 1.07E-02 | At1g56430 | nicotianamine synthase, putative |
|  | 418 | | -0.48 | 11.21 | 1.07E-02 | At1g22750 | expressed protein |
|  | 419 | | 0.59 | 9.79 | 1.07E-02 | At5g10770 | nucleoid DNA-binding protein cnd41 - like protein |
|  | 420 | | 0.55 | 9.35 | 1.07E-02 | At3g59020 | importin beta family |
|  | 421 | | 0.78 | 11.03 | 1.07E-02 | At5g08460 | GDSL-motif lipase/hydrolase protein |
|  | 422 | | -0.51 | 11.99 | 1.08E-02 | At5g26667 | predicted protein |
|  | 423 | | -0.56 | 10.04 | 1.08E-02 |  | Arabidopsis thaliana DNA chromosome 4, BAC clone F24J7 (ESSA project) |
|  | 424 | | 0.55 | 10.12 | 1.08E-02 | At3g19170 | metalloprotease -related |
|  | 425 | | -0.72 | 11.17 | 1.08E-02 | At1g03430 | AHP2 -related |
|  | 426 | | 0.69 | 11.14 | 1.08E-02 | At4g25630 | fibrillarin 2 (AtFib2) |
|  | 427 | | 0.98 | 11.48 | 1.08E-02 | At5g14380 | arabinogalactan-protein (AGP6) |
|  | 428 | | -0.70 | 11.25 | 1.08E-02 | At1g50900 | expressed protein |
|  | 429 | | -0.54 | 9.89 | 1.08E-02 | At3g09020 | glycosyltransferase-related |
|  | 430 | | -0.69 | 12.44 | 1.08E-02 | At3g48180 | hypothetical protein |
|  | 431 | | -0.59 | 12.55 | 1.08E-02 | At3g19010 | flavonol synthase family |
|  | 432 | | -0.55 | 10.83 | 1.09E-02 | At1g69460 | emp24/gp25L/p24 family |
|  | 433 | | 0.51 | 10.72 | 1.09E-02 | At1g34220 | expressed protein |
|  | 434 | | 0.93 | 13.52 | 1.09E-02 | At5g12460 | fringe-related protein |
|  | 435 | | 0.63 | 11.08 | 1.09E-02 | At5g66120 | 3-dehydroquinate synthase, putative |
|  | 436 | | 0.57 | 11.08 | 1.09E-02 | At3g25910 | expressed protein |
|  | 437 | | -0.63 | 9.24 | 1.09E-02 | At3g15115 | expressed protein |
|  | 438 | | -0.78 | 11.03 | 1.09E-02 | At4g21840 | expressed protein |
|  | 439 | | -0.64 | 9.70 | 1.09E-02 | At4g36850 | expressed protein |
|  | 440 | | -1.44 | 10.57 | 1.09E-02 | At1g22990 | copper chaperone (CCH)-related |
|  | 441 | | 0.50 | 12.67 | 1.10E-02 | At5g42650 | allene oxide synthase / cytochrome P450 74A |
|  | 442 | | -0.87 | 13.78 | 1.10E-02 | At2g43510 | trypsin inhibitor -related |
|  | 443 | | 0.46 | 9.92 | 1.10E-02 | At5g43960 | NTF2-containing RNA-binding protein, putative |
|  | 444 | | -0.78 | 13.68 | 1.10E-02 | At5g64040 | photosystem I reaction center subunit PSI-N precursor (PSI-N) |
|  | 445 | | -0.75 | 11.69 | 1.11E-02 | At5g09830 | expressed protein |
|  | 446 | | -0.77 | 9.68 | 1.11E-02 | At1g22180 | expressed protein |
|  | 447 | | 0.73 | 11.59 | 1.11E-02 | At5g40850 | urophorphyrin III methylase (gb|AAB92676.1) |
|  | 448 | | -0.42 | 9.80 | 1.11E-02 | At3g10180 | kinesin-related protein |
|  | 449 | | -0.51 | 11.94 | 1.11E-02 | At2g17560 | HMG protein -related |
|  | 450 | | 0.78 | 11.71 | 1.11E-02 | At4g24280 | heat shock protein cpHsc70-1 |
|  | 451 | | 0.82 | 13.97 | 1.12E-02 | At5g57070 | proline-rich protein family |
|  | 452 | | -1.26 | 13.41 | 1.12E-02 | At5g44580 | expressed protein |
|  | 453 | | -0.51 | 10.65 | 1.13E-02 | At1g80940 | expressed protein |
|  | 454 | | 1.15 | 12.65 | 1.13E-02 | At1g15850 | transducin / WD-40 repeat protein family |
|  | 455 | | -0.60 | 11.96 | 1.13E-02 | At3g48570 | expressed protein |
|  | 456 | | 0.60 | 10.61 | 1.13E-02 | At1g70620 | expressed protein |
|  | 457 | | 0.55 | 11.81 | 1.14E-02 | At5g24360 | protein kinase family |
|  | 458 | | -0.62 | 15.06 | 1.14E-02 | At4g03280 | Rieske FeS protein (component of cytochrome B6-F complex) |
|  | 459 | | 0.62 | 11.69 | 1.14E-02 | At3g09710 | SF16 protein -related |
|  | 460 | | 0.47 | 12.28 | 1.15E-02 | At1g10760 | SEX1 protein; nuclear gene for chloroplast product |
|  | 461 | | 0.62 | 10.75 | 1.15E-02 | At2g30490 | cytochrome P450 73 / trans-cinnamate 4-monooxygenase / cinnamate-4-hydroxylase (CYP73) (C4H) |
|  | 462 | | 0.86 | 11.30 | 1.16E-02 | At5g63720 | hypothetical protein |
|  | 463 | | -0.52 | 10.19 | 1.17E-02 | At2g38130 | N-acetyltransferase, putative |
|  | 464 | | -0.67 | 11.25 | 1.17E-02 | At1g65845 | expressed protein |
|  | 465 | | -0.75 | 13.42 | 1.17E-02 | At5g13190 | expressed protein |
|  | 466 | | 0.54 | 11.31 | 1.17E-02 | At3g53510 | ABC transporter family protein |
|  | 467 | | -1.01 | 14.14 | 1.17E-02 | At3g04720 | hevein-related protein precursor (PR-4) |
|  | 468 | | -0.62 | 14.36 | 1.17E-02 | At1g76960 | expressed protein |
|  | 469 | | -0.53 | 11.09 | 1.18E-02 | At3g42150 | expressed protein |
|  | 470 | | -0.49 | 11.80 | 1.18E-02 | At1g56570 | pentatricopeptide (PPR) repeat-containing protein |
|  | 471 | | 0.70 | 10.72 | 1.18E-02 | At1g54000 | myrosinase-associated protein, putative |
|  | 472 | | 0.51 | 9.37 | 1.18E-02 | At1g76460 | RNA recognition motif (RRM)-containing protein |
|  | 473 | | 0.54 | 10.81 | 1.18E-02 | At5g64270 | splicing factor, putative |
|  | 474 | | 0.55 | 10.16 | 1.19E-02 | At5g47560 | Arabidopsis thaliana mRNA for sodium sulfate or dicarboxylate transporter, complete cds |
|  | 475 | | 0.96 | 14.04 | 1.19E-02 | At5g48920 | proline-rich protein family |
|  | 476 | | 0.50 | 12.52 | 1.19E-02 | At5g67420 | lateral organ boundaries (LOB) domain protein 37 (LBD37) |
|  | 477 | | 0.59 | 10.32 | 1.19E-02 | At1g53570 | mitogen-activated protein kinase kinase kinase (MAPKKK), putative (MAP3Ka) |
|  | 478 | | 0.57 | 11.44 | 1.19E-02 | At5g65110 | EST |
|  | 479 | | -0.70 | 12.94 | 1.19E-02 | At3g28930 | AIG2-related protein |
|  | 480 | | 1.38 | 10.60 | 1.19E-02 | At3g08860 | alanine--glyoxylate aminotransferase (beta-alanine-pyruvate aminotransferase/AGT), putative |
|  | 481 | | -0.61 | 10.75 | 1.20E-02 | At5g03690 | fructose-bisphosphate aldolase, putative |
|  | 482 | | -0.90 | 10.74 | 1.20E-02 | At2g38450 | Arabidopsis thaliana chromosome II section 207 of 255 of the complete sequence. |
|  | 483 | | 0.78 | 10.75 | 1.21E-02 | At2g44550 | glycosyl hydrolase family 9 |
|  | 484 | | -0.60 | 12.33 | 1.22E-02 | At2g02930 | glutathione transferase, putative |
|  | 485 | | -0.50 | 10.10 | 1.22E-02 | At1g70800 | hypothetical protein |
|  | 486 | | 0.88 | 12.09 | 1.22E-02 | At3g53530 | heavy-metal-associated domain-containing protein |
|  | 487 | | 0.60 | 10.56 | 1.23E-02 | At1g54490 | exonuclease -related |
|  | 488 | | -0.83 | 11.05 | 1.23E-02 | At5g10810 | enhancer of rudimentary |
|  | 489 | | -0.55 | 10.21 | 1.23E-02 | At1g77080 | MADS affecting flowering 1(MAF1) |
|  | 490 | | -0.86 | 13.55 | 1.23E-02 | At2g26020 | plant defensin protein, putative (PDF1.2b) |
|  | 491 | | 0.70 | 11.10 | 1.23E-02 | At1g69870 | peptide transporter -related |
|  | 492 | | 0.66 | 12.00 | 1.23E-02 | At5g03120 | expressed protein |
|  | 493 | | 0.67 | 10.89 | 1.23E-02 | At2g26690 | nitrate transporter -related |
|  | 494 | | -1.16 | 14.69 | 1.23E-02 | At5g20150 | ids4-related protein |
|  | 495 | | -0.57 | 12.26 | 1.23E-02 | At3g23325 | expressed protein |
|  | 496 | | -0.96 | 12.67 | 1.23E-02 | At1g22630 | auxin-regulated protein |
|  | 497 | | 0.56 | 11.96 | 1.23E-02 | At5g65660 | proline-rich protein-related |
|  | 498 | | 0.89 | 12.10 | 1.23E-02 | At4g01720 | WRKY family transcription factor |
|  | 499 | | 0.51 | 13.17 | 1.23E-02 | At3g48490 | Arabidopsis thaliana DNA chromosome 3, BAC clone T29H11 |
|  | 500 | | -1.31 | 10.46 | 1.23E-02 | At5g61980 | ARF GTPase-activating domain-containing protein |
|  | 501 | | -0.45 | 12.12 | 1.23E-02 | At3g57930 | expressed protein |
|  | 502 | | -0.69 | 12.54 | 1.23E-02 | At5g67490 | Arabidopsis thaliana genomic DNA, chromosome 5, TAC clone:K9I9 |
|  | 503 | | -0.48 | 10.21 | 1.23E-02 | At1g76405 | auxin-regulated protein |
|  | 504 | | -0.46 | 12.55 | 1.23E-02 | At4g32060 | calcium-binding EF-hand family protein |
|  | 505 | | -0.52 | 9.93 | 1.23E-02 | At4g18400 | expressed protein |
|  | 506 | | 0.84 | 11.21 | 1.24E-02 | At3g11130 | clathrin heavy chain -related |
|  | 507 | | -1.22 | 13.03 | 1.24E-02 | At5g44580 | EST |
|  | 508 | | -0.43 | 11.17 | 1.24E-02 | At1g05385 | expressed protein |
|  | 509 | | -0.61 | 10.04 | 1.24E-02 | At4g33985 | expressed protein |
|  | 510 | | 0.40 | 13.57 | 1.24E-02 | At5g50920 | ATP-dependent Clp protease ATP-binding subunit (ClpC1) |
|  | 511 | | 0.40 | 9.67 | 1.24E-02 | At4g23980 | auxin response transcription factor (ARF9) |
|  | 512 | | -0.46 | 9.32 | 1.27E-02 | At4g23610 | expressed protein |
|  | 513 | | 0.77 | 10.84 | 1.27E-02 | At4g02480 | expressed protein |
|  | 514 | | 0.62 | 10.85 | 1.27E-02 | At1g34420 | leucine rich repeat protein kinase family |
|  | 515 | | -0.43 | 9.70 | 1.27E-02 | At3g22060 | receptor protein kinase-related |
|  | 516 | | 0.58 | 12.39 | 1.27E-02 | At5g13510 | ribosomal protein L10p family |
|  | 517 | | 0.92 | 10.15 | 1.27E-02 | At4g17770 | trehalose phosphatase family |
|  | 518 | | -0.52 | 11.77 | 1.28E-02 | At3g18140 | transducin / WD-40 repeat protein family |
|  | 519 | | 0.45 | 13.75 | 1.29E-02 | At2g15970 | cold acclimation protein WCOR413 [Triticum aestivum] -related |
|  | 520 | | -0.53 | 10.26 | 1.29E-02 | At1g27385 | expressed protein |
|  | 521 | | 0.62 | 12.08 | 1.29E-02 | At4g36240 | GATA zinc finger protein |
|  | 522 | | 0.47 | 10.86 | 1.30E-02 | At5g03650 | 1,4-alpha-glucan branching enzyme (starch branching enzyme class II/sbe2-2) |
|  | 523 | | 0.63 | 10.24 | 1.32E-02 | At3g13930 | acetyltransferase -related |
|  | 524 | | -0.56 | 9.74 | 1.32E-02 | At3g05920 | heavy-metal-associated domain-containing protein |
|  | 525 | | 0.81 | 11.64 | 1.32E-02 | At3g15000 | expressed protein |
|  | 526 | | 0.47 | 9.96 | 1.32E-02 | At2g28520 | vacuolar proton-ATPase subunit -related |
|  | 527 | | 0.62 | 9.87 | 1.32E-02 | At4g00040 | chalcone and stilbene synthase family |
|  | 528 | | -0.90 | 12.06 | 1.33E-02 | At1g79520 | Arabidopsis thaliana chromosome 1 BAC T8K14 sequence |
|  | 529 | | 0.97 | 12.69 | 1.34E-02 | At2g30740 | serine/threonine protein kinase, putative |
|  | 530 | | 0.53 | 10.54 | 1.35E-02 | At2g20520 | fasciclin-like arabinogalactan-protein (FLA6) |
|  | 531 | | -0.98 | 12.69 | 1.35E-02 | At5g54490 | calcium-binding protein, putative |
|  | 532 | | 0.54 | 8.89 | 1.35E-02 | At5g17270 | tetratricopeptide repeat (TPR)-containing protein |
|  | 533 | | -0.86 | 12.36 | 1.35E-02 | At5g03210 | expressed protein |
|  | 534 | | -0.89 | 12.16 | 1.35E-02 | At2g42530 | cold-regulated protein (cor15b) |
|  | 535 | | 0.86 | 11.72 | 1.35E-02 | At4g18020 | pseudo-response regulator APRR1(TOC1) |
|  | 536 | | 0.58 | 11.38 | 1.36E-02 | At3g47700 | expressed protein |
|  | 537 | | -0.63 | 11.88 | 1.36E-02 | At1g74670 | GAST1-related protein |
|  | 538 | | 0.84 | 12.99 | 1.36E-02 | At3g09440 | heat shock protein hsc70-3 (hsc70.3) |
|  | 539 | | -0.49 | 15.82 | 1.36E-02 | At3g09390 | metallothionein-related protein |
|  | 540 | | 0.54 | 13.63 | 1.38E-02 | At1g75000 | hypothetical protein |
|  | 541 | | 0.95 | 10.11 | 1.38E-02 | At1g72520 | lipoxygenase (LOX), putative |
|  | 542 | | -0.51 | 11.58 | 1.38E-02 | At2g21290 | expressed protein |
|  | 543 | | -0.73 | 11.61 | 1.38E-02 | At5g06690 | thioredoxin family |
|  | 544 | | 0.90 | 11.89 | 1.38E-02 | At5g53950 | No apical meristem (NAM) protein CUC2 |
|  | 545 | | -0.41 | 11.58 | 1.40E-02 | At5g57860 | ubiquitin family |
|  | 546 | | -0.52 | 10.44 | 1.40E-02 | At2g24390 | Arabidopsis thaliana chromosome II section 137 of 255 of the complete sequence. |
|  | 547 | | -0.54 | 11.27 | 1.40E-02 | At2g29310 | short-chain dehydrogenase/reductase family protein (tropinone reductase, putative) |
|  | 548 | | -0.73 | 10.13 | 1.40E-02 | At1g47820 | expressed protein |
|  | 549 | | 0.45 | 9.72 | 1.40E-02 | At5g41110 | hypothetical protein |
|  | 550 | | -0.51 | 10.08 | 1.40E-02 | At3g62820 | pectinesterase - related |
|  | 551 | | 0.50 | 9.79 | 1.40E-02 | At4g38160 | expressed protein |
|  | 552 | | 0.51 | 10.62 | 1.40E-02 | At1g53510 | mitogen-activated protein kinase (MAPK), putative (MPK18) |
|  | 553 | | -0.46 | 9.88 | 1.40E-02 | At3g15420 | hypothetical protein |
|  | 554 | | -0.64 | 11.65 | 1.40E-02 | At3g59810 | U6 snRNA-associated Sm-related protein |
|  | 555 | | 0.46 | 12.14 | 1.41E-02 | At3g14100 | oligouridylate binding protein (UBP1), putative |
|  | 556 | | 0.69 | 10.24 | 1.41E-02 | At3g06380 | F-box containing tubby family protein |
|  | 557 | | -0.69 | 14.86 | 1.41E-02 | At1g32920 | expressed protein |
|  | 558 | | 0.67 | 10.57 | 1.41E-02 | At3g20470 | glycine-rich protein |
|  | 559 | | -0.96 | 10.98 | 1.41E-02 | At3g03820 | auxin-induced (indole-3-acetic acid induced) protein, putative |
|  | 560 | | 0.54 | 9.53 | 1.41E-02 | At4g24680 | expressed protein |
|  | 561 | | 0.43 | 9.26 | 1.41E-02 | At2g18790 | phytochrome B (PHYB) |
|  | 562 | | -0.56 | 13.44 | 1.41E-02 | At4g24920 | PROTEIN TRANSPORT PROTEIN SEC61 GAMMA SUBUNIT -related |
|  | 563 | | 0.45 | 10.06 | 1.42E-02 | At1g17580 | myosin -related |
|  | 564 | | -0.53 | 11.90 | 1.42E-02 | At4g12490 | protease inhibitor/seed storage/lipid transfer protein (LTP) family |
|  | 565 | | 0.43 | 9.84 | 1.42E-02 | At5g24760 | alcohol dehydrogenase (ADH), putative |
|  | 566 | | 0.59 | 10.66 | 1.42E-02 | At5g04660 | cytochrome P450, putative |
|  | 567 | | 0.62 | 10.17 | 1.42E-02 | At4g32410 | cellulose synthase, catalytic subunit, putative |
|  | 568 | | -0.47 | 14.55 | 1.43E-02 | At2g41110 | calmodulin |
|  | 569 | | -0.76 | 10.13 | 1.43E-02 | At1g57630 | disease resistance protein (TIR class), putative |
|  | 570 | | -0.58 | 9.21 | 1.43E-02 | At1g11920 | polysaccharide lyase family 1 (pectate lyase) |
|  | 571 | | 0.43 | 10.61 | 1.43E-02 | At1g75780 | Arabidopsis thaliana beta-1 tubulin gene, complete cds |
|  | 572 | | -1.12 | 12.51 | 1.43E-02 | At1g13245 | expressed protein |
|  | 573 | | -0.66 | 11.54 | 1.44E-02 | At3g27050 | expressed protein |
|  | 574 | | -0.73 | 13.75 | 1.45E-02 | At2g24940 | cytochrome b5 domain-containing protein |
|  | 575 | | 1.09 | 11.55 | 1.45E-02 | At5g16960 | NADP-dependent oxidoreductase, putative |
|  | 576 | | -0.39 | 11.02 | 1.45E-02 | At3g47860 | expressed protein |
|  | 577 | | 0.50 | 10.69 | 1.45E-02 | At5g02260 | expansin, putative (EXP9) |
|  | 578 | | 0.50 | 11.04 | 1.45E-02 | At5g16730 | expressed protein |
|  | 579 | | -0.54 | 11.35 | 1.45E-02 | At2g39470 | oxygen-evolving complex 25.6 kD protein, chloroplast precursor, putative |
|  | 580 | | -1.61 | 11.32 | 1.45E-02 | At1g75040 | pathogenesis-related protein 5 (PR-5) |
|  | 581 | | 0.48 | 10.10 | 1.46E-02 | At3g49490 | expressed protein |
|  | 582 | | 0.55 | 11.19 | 1.46E-02 | At1g09140 | arginine/serine-rich splicing factor, atSRp30 |
|  | 583 | | 0.52 | 11.35 | 1.46E-02 | At1g69120 | floral homeotic gene APETALA1 |
|  | 584 | | 0.55 | 12.23 | 1.47E-02 | At1g52720 | expressed protein |
|  | 585 | | 0.75 | 13.89 | 1.47E-02 | At3g44300 | nitrilase 2 |
|  | 586 | | 0.46 | 11.14 | 1.47E-02 | At4g02340 | epoxide hydrolase, putative |
|  | 587 | | 0.54 | 9.28 | 1.47E-02 | At1g15290 | tetratricopeptide repeat (TPR)-containing protein |
|  | 588 | | 0.56 | 9.54 | 1.47E-02 | At3g12520 | sulphate transporter -related |
|  | 589 | | 0.61 | 9.34 | 1.47E-02 | At3g03480 | transferase family |
|  | 590 | | 0.72 | 11.54 | 1.47E-02 | At1g78300 | 14-3-3 protein GF14 omega (grf2) |
|  | 591 | | -0.75 | 11.23 | 1.49E-02 | At1g70810 | C2 domain-containing protein |
|  | 592 | | 0.72 | 11.69 | 1.49E-02 | At5g49910 | heat shock protein cpHsc70-2 (hsc70-7) |
|  | 593 | | 0.48 | 13.33 | 1.49E-02 | At3g06130 | heavy-metal-associated domain-containing protein |
|  | 594 | | -0.87 | 10.42 | 1.49E-02 | At2g43380 | expressed protein |
|  | 595 | | -0.51 | 12.87 | 1.49E-02 | At3g09735 | expressed protein |
|  | 596 | | -0.52 | 11.64 | 1.49E-02 | At2g43810 | small nuclear ribonucleo protein polypeptide F -related |
|  | 597 | | -0.63 | 11.60 | 1.49E-02 | At5g48790 | expressed protein |
|  | 598 | | 0.78 | 9.94 | 1.49E-02 | At3g61270 | expressed protein |
|  | 599 | | -0.42 | 12.58 | 1.49E-02 | At2g28740 | histone H4 |
|  | 600 | | 0.53 | 10.74 | 1.49E-02 | At3g14010 | expressed protein |
|  | 601 | | 0.50 | 9.73 | 1.49E-02 | At1g04430 | Sequence of BAC F19P19 from Arabidopsis thaliana chromosome 1 |
|  | 602 | | 0.48 | 9.25 | 1.50E-02 | At3g13860 | chaperonin, putative |
|  | 603 | | -0.53 | 11.39 | 1.50E-02 | At5g24210 | lipase (class 3) family |
|  | 604 | | -0.49 | 11.05 | 1.50E-02 | At5g65860 | hypothetical protein |
|  | 605 | | 0.50 | 10.06 | 1.52E-02 | At3g11330 | leucine rich repeat protein-related |
|  | 606 | | 0.41 | 12.08 | 1.52E-02 | At5g02870 | 60S ribosomal protein L4/L1 (RPL4D) |
|  | 607 | | 0.78 | 12.29 | 1.52E-02 | At2g43160 | clathrin binding protein (epsin) -related |
|  | 608 | | -0.46 | 11.48 | 1.52E-02 | At1g71950 | expressed protein |
|  | 609 | | -0.42 | 13.99 | 1.53E-02 | At3g58680 | transcriptional coactivator - like protein |
|  | 610 | | -0.49 | 12.21 | 1.53E-02 | At2g02320 | F-box protein (SKP1 interacting partner 3-related) |
|  | 611 | | 0.64 | 10.57 | 1.53E-02 | At2g29210 | proline-rich protein -related |
|  | 612 | | -0.56 | 9.78 | 1.53E-02 | At5g48600 | EST |
|  | 613 | | -0.37 | 10.30 | 1.53E-02 | At3g12650 | expressed protein |
|  | 614 | | -0.58 | 10.17 | 1.53E-02 | At1g44000 | expressed protein |
|  | 615 | | -0.40 | 9.32 | 1.53E-02 | At3g46580 | expressed protein |
|  | 616 | | 0.52 | 10.68 | 1.53E-02 | At4g37910 | heat shock protein mtHsc70-1 |
|  | 617 | | 0.73 | 10.66 | 1.53E-02 | At1g29900 | carbamoylphosphate synthetase -related |
|  | 618 | | -0.49 | 9.48 | 1.53E-02 | At3g25240 | hypothetical protein |
|  | 619 | | 1.03 | 12.81 | 1.53E-02 | At1g79110 | expressed protein |
|  | 620 | | -0.70 | 9.82 | 1.53E-02 | At2g38700 | Arabidopsis thaliana mRNA for mevalonate diphosphate decarboxylase |
|  | 621 | | 0.65 | 11.81 | 1.53E-02 | At3g08590 | 2,3-bisphosphoglycerate-independent phosphoglycerate mutase -related |
|  | 622 | | -0.55 | 10.09 | 1.54E-02 | At4g00680 | actin-depolymerizing factor -related |
|  | 623 | | 0.55 | 10.64 | 1.56E-02 | At4g29010 | abnormal inflorescence meristem 1 (fatty acid multifunctional protein) (AIM1) |
|  | 624 | | -0.51 | 9.61 | 1.56E-02 | At1g19330 | expressed protein |
|  | 625 | | 0.51 | 9.45 | 1.56E-02 | At5g65500 | protein kinase family |
|  | 626 | | 0.89 | 11.30 | 1.57E-02 | At2g21390 | coatomer alpha subunit |
|  | 627 | | 0.76 | 10.08 | 1.57E-02 | At1g18040 | cell division protein kinase, putative |
|  | 628 | | -0.52 | 12.13 | 1.57E-02 | At2g45860 | expressed protein |
|  | 629 | | -0.51 | 14.74 | 1.57E-02 | At1g51400 | photosystem II 5 KD protein |
|  | 630 | | 0.71 | 11.64 | 1.57E-02 | At5g49910 | heat shock protein cpHsc70-2 (hsc70-7) |
|  | 631 | | 0.60 | 10.76 | 1.57E-02 | At1g03080 | expressed protein |
|  | 632 | | -0.57 | 9.76 | 1.57E-02 | At5g24420 | 6-phosphogluconolactonase-related protein |
|  | 633 | | -0.40 | 11.32 | 1.58E-02 | At3g48030 | zinc finger (C3HC4-type RING finger) protein family |
|  | 634 | | 0.54 | 11.02 | 1.58E-02 | At3g60100 | citrate synthase-related protein |
|  | 635 | | -0.58 | 14.01 | 1.58E-02 | At2g25210 | 60S ribosomal protein L39 (RPL39A) |
|  | 636 | | -0.48 | 10.88 | 1.58E-02 | At1g52600 | signal peptidase subunit -related |
|  | 637 | | -0.65 | 9.79 | 1.58E-02 | At5g47710 | C2 domain-containing protein |
|  | 638 | | 0.42 | 10.82 | 1.59E-02 | At3g15880 | WD-repeat protein -related |
|  | 639 | | -0.62 | 10.64 | 1.60E-02 | At4g05620 | F-box protein family |
|  | 640 | | 0.52 | 13.28 | 1.60E-02 | At5g09590 | heat shock protein mtHsc70-2 (Hsc70-5) |
|  | 641 | | -0.46 | 10.08 | 1.60E-02 | At5g38020 | S-adenosyl-L-methionine:carboxyl methyltransferase family |
|  | 642 | | 0.42 | 10.75 | 1.60E-02 | At3g47520 | malate dehydrogenase [NAD], chloroplast, putative |
|  | 643 | | 0.73 | 10.32 | 1.60E-02 | At2g32560 | F-box protein family |
|  | 644 | | 0.46 | 11.83 | 1.60E-02 | At5g61020 | expressed protein |
|  | 645 | | -0.56 | 11.27 | 1.60E-02 | At4g15930 | dynein light chain like protein |
|  | 646 | | -0.72 | 10.55 | 1.60E-02 | At1g04240 | auxin-responsive protein IAA3 (Indoleacetic acid-induced protein 3) |
|  | 647 | | -0.65 | 10.58 | 1.60E-02 | At1g34570 | EST |
|  | 648 | | 0.88 | 11.43 | 1.60E-02 | At3g15690 | acetyl-CoA carboxylase biotin-containing subunit -related |
|  | 649 | | -0.58 | 10.20 | 1.60E-02 | At1g33960 | AIG1 |
|  | 650 | | 0.59 | 12.43 | 1.60E-02 | At4g08455 | Arabidopsis thaliana BAC T15F16 |
|  | 651 | | 0.66 | 11.47 | 1.60E-02 | At3g29590 | transferase family |
|  | 652 | | -0.50 | 10.79 | 1.60E-02 | At3g49100 | signal recognition particle subunit 9 - like |
|  | 653 | | -0.70 | 10.92 | 1.60E-02 | At4g22570 | adenine phosphoribosyltransferase (EC 2.4.2.7) - like protein |
|  | 654 | | 0.72 | 10.99 | 1.62E-02 | At4g28470 | hypothetical protein |
|  | 655 | | 0.74 | 11.71 | 1.62E-02 | At3g44060 | F-box protein family |
|  | 656 | | -0.54 | 10.84 | 1.62E-02 | At3g57090 | expressed protein |
|  | 657 | | -0.61 | 11.29 | 1.62E-02 | At5g59140 | SKP1 family |
|  | 658 | | -0.77 | 12.22 | 1.62E-02 | At4g11890 | protein kinase family |
|  | 659 | | -0.62 | 11.30 | 1.62E-02 | At3g15480 | expressed protein |
|  | 660 | | 0.58 | 11.43 | 1.63E-02 | At2g01600 | expressed protein |
|  | 661 | | 0.47 | 9.76 | 1.63E-02 | At5g50380 | exocyst subunit EXO70 family |
|  | 662 | | -0.44 | 10.37 | 1.64E-02 | At2g18250 | cytidylyltransferase domain-containing protein |
|  | 663 | | 0.53 | 12.91 | 1.64E-02 | At4g32020 | Arabidopsis thaliana AT4g32020/F10N7_170 mRNA, complete cds |
|  | 664 | | 0.50 | 10.77 | 1.64E-02 | At3g15020 | malate dehydrogenase [NAD], mitochondrial, putative |
|  | 665 | | 0.49 | 11.80 | 1.65E-02 | At4g39080 | proton pump -related |
|  | 666 | | -0.38 | 10.31 | 1.65E-02 | At2g42870 | expressed protein |
|  | 667 | | 0.54 | 10.63 | 1.65E-02 | At3g47960 | peptide transporter -related |
|  | 668 | | -0.69 | 11.80 | 1.66E-02 | At5g67620 | expressed protein |
|  | 669 | | 0.45 | 9.50 | 1.66E-02 | At5g03020 | Kelch repeat containing F-box protein family |
|  | 670 | | 0.59 | 10.00 | 1.66E-02 | At2g13370 | chromodomain-helicase-DNA-binding (CHD) protein family |
|  | 671 | | 0.52 | 11.57 | 1.66E-02 | At3g01470 | homeobox-leucine zipper protein HAT5 (HD-ZIP protein 5) (HD-ZIP protein ATHB-1) |
|  | 672 | | -0.76 | 10.30 | 1.66E-02 | At2g34720 | CCAAT-binding transcription factor subunit -related |
|  | 673 | | 0.55 | 11.19 | 1.66E-02 | At1g28400 | expressed protein |
|  | 674 | | -0.53 | 11.20 | 1.66E-02 | At2g26340 | expressed protein |
|  | 675 | | 0.92 | 13.83 | 1.66E-02 | At3g03230 | esterase/lipase/thioesterase family |
|  | 676 | | -0.51 | 11.30 | 1.67E-02 | At2g28430 | expressed protein |
|  | 677 | | 0.41 | 12.01 | 1.67E-02 | At1g02500 | s-adenosylmethionine synthetase |
|  | 678 | | 0.60 | 10.64 | 1.67E-02 | At1g29150 | 26S proteasome regulatory subunit (RPN6), putative |
|  | 679 | | -0.52 | 11.59 | 1.67E-02 | At5g05360 | expressed protein |
|  | 680 | | 0.79 | 10.36 | 1.68E-02 | At2g41220 | glutamate synthase [ferredoxin], chloroplast |
|  | 681 | | 0.80 | 12.14 | 1.68E-02 | At1g69780 | homeobox-leucine zipper protein ATHB-13 (HD-Zip transcription factor Athb-13) |
|  | 682 | | 0.64 | 11.51 | 1.68E-02 | At4g28080 | expressed protein |
|  | 683 | | -0.43 | 11.25 | 1.69E-02 | At4g18580 | expressed protein |
|  | 684 | | -0.60 | 11.89 | 1.69E-02 | At2g34585 | expressed protein |
|  | 685 | | -0.53 | 12.07 | 1.69E-02 | At2g31490 | expressed protein |
|  | 686 | | 0.64 | 9.49 | 1.70E-02 | At1g20760 | calcium-binding EF-hand family protein |
|  | 687 | | -0.49 | 11.98 | 1.70E-02 | At5g51020 | expressed protein |
|  | 688 | | -0.43 | 11.11 | 1.70E-02 | At5g48630 | cyclin family |
|  | 689 | | 0.85 | 10.67 | 1.70E-02 | At2g25200 | expressed protein |
|  | 690 | | 0.61 | 11.73 | 1.70E-02 | At3g04240 | glycosyltransferase - related |
|  | 691 | | 0.71 | 10.19 | 1.70E-02 | At4g22670 | tetratricopeptide repeat (TPR)-containing protein |
|  | 692 | | -0.64 | 14.09 | 1.71E-02 | At1g15270 | expressed protein |
|  | 693 | | 0.48 | 9.37 | 1.71E-02 | At5g46610 | hypothetical protein |
|  | 694 | | 0.55 | 10.26 | 1.71E-02 | At3g18010 | hypothetical protein |
|  | 695 | | -0.48 | 9.77 | 1.72E-02 | At1g15520 | ABC transporter family protein |
|  | 696 | | 0.61 | 11.64 | 1.73E-02 | At4g36080 | FAT domain-containing phosphatidylinositol 3- and 4-kinase family |
|  | 697 | | -0.62 | 9.80 | 1.73E-02 | At1g05810 | RAS-related GTP-binding protein (ARA-1) |
|  | 698 | | -1.32 | 10.28 | 1.73E-02 | At5g55450 | protease inhibitor/seed storage/lipid transfer protein (LTP) family |
|  | 699 | | 0.50 | 11.45 | 1.74E-02 | At5g42080 | dynamin-related protein (pir||S59558) |
|  | 700 | | -0.72 | 9.74 | 1.74E-02 | At1g65310 | xyloglucan endotransglycosylase, putative |
|  | 701 | | 0.42 | 9.35 | 1.75E-02 | At1g79730 | proline-rich protein family |
|  | 702 | | 0.72 | 9.79 | 1.76E-02 | At3g07770 | heat shock protein -related |
|  | 703 | | 0.58 | 10.10 | 1.76E-02 | At5g61640 | peptide methionine sulfoxide reductase - like protein |
|  | 704 | | -0.55 | 13.59 | 1.77E-02 | At2g23120 | expressed protein |
|  | 705 | | -0.58 | 12.05 | 1.77E-02 | At1g32460 | expressed protein |
|  | 706 | | 0.67 | 14.10 | 1.77E-02 | At3g60750 | transketolase - like protein |
|  | 707 | | 0.50 | 9.27 | 1.77E-02 | At1g14710 | proline-rich protein family |
|  | 708 | | -0.58 | 11.11 | 1.77E-02 | At1g53030 | cytochrome C oxidase assembly protein -related |
|  | 709 | | 0.47 | 9.11 | 1.77E-02 | At4g36570 | myb family transcription factor |
|  | 710 | | -0.62 | 10.85 | 1.77E-02 | At5g37070 | hypothetical protein |
|  | 711 | | -0.68 | 11.35 | 1.77E-02 | At1g53640 | hypothetical protein |
|  | 712 | | 0.43 | 13.06 | 1.77E-02 | At5g53750 | expressed protein |
|  | 713 | | 0.44 | 12.42 | 1.78E-02 | At3g04840 | 40S ribosomal protein S3A (RPS3aA) |
|  | 714 | | -0.65 | 10.90 | 1.78E-02 | At3g60640 | (AtAPG8g) autophagy 8g protein |
|  | 715 | | 0.51 | 10.70 | 1.78E-02 | At1g26110 | expressed protein |
|  | 716 | | 0.69 | 9.49 | 1.78E-02 | At2g22510 | proline-rich protein family |
|  | 717 | | -0.54 | 10.02 | 1.78E-02 | At5g60220 | senescence-associated protein family |
|  | 718 | | 0.87 | 10.66 | 1.78E-02 | At5g43770 | proline-rich protein family |
|  | 719 | | 0.46 | 10.63 | 1.78E-02 | At4g00720 | Shaggy related protein kinase tetha |
|  | 720 | | -0.63 | 11.77 | 1.79E-02 | At1g76650 | calcium-binding EF-hand family protein |
|  | 721 | | -0.70 | 15.13 | 1.79E-02 | At2g35370 | glycine decarboxylase complex H-protein |
|  | 722 | | -0.51 | 9.61 | 1.80E-02 | At5g36970 | hypothetical protein |
|  | 723 | | -0.68 | 12.23 | 1.80E-02 | At2g32650 | expressed protein |
|  | 724 | | -0.77 | 11.19 | 1.80E-02 | At1g66820 | glycine-rich protein |
|  | 725 | | 0.39 | 10.29 | 1.80E-02 | At2g39670 | expressed protein |
|  | 726 | | 0.63 | 11.30 | 1.80E-02 | At4g22540 | expressed protein |
|  | 727 | | 0.99 | 10.49 | 1.80E-02 | At1g80070 | splicing factor Prp8 -related |
|  | 728 | | -1.84 | 12.29 | 1.80E-02 | At2g14610 | pathogenesis-related protein 1 (PR-1) |
|  | 729 | | 0.49 | 10.21 | 1.80E-02 | At3g55210 | NAM (no apical meristem) -related protein |
|  | 730 | | -0.74 | 11.69 | 1.80E-02 | At5g03545 | induced by Pi starvation (At4) |
|  | 731 | | -0.54 | 12.96 | 1.80E-02 | At1g05010 | 1-aminocyclopropane-1-carboxylate oxidase (ACC oxidase) (ethylene-forming enzyme) (EFE) |
|  | 732 | | 0.49 | 9.54 | 1.80E-02 | At4g01290 | Arabidopsis thaliana DNA chromosome 4, contig fragment No. 3 |
|  | 733 | | -0.46 | 10.87 | 1.80E-02 | At3g07080 | membrane protein |
|  | 734 | | -0.57 | 10.23 | 1.80E-02 | At3g49940 | lateral organ boundaries (LOB) domain protein, putative |
|  | 735 | | 0.58 | 13.99 | 1.80E-02 | At4g09480 | hypothetical protein |
|  | 736 | | 0.54 | 9.81 | 1.80E-02 | At5g15780 | proline-rich protein |
|  | 737 | | -0.63 | 10.24 | 1.80E-02 |  | Arabidopsis thaliana genomic DNA, chromosome 5, BAC clone:F16F17 |
|  | 738 | | 0.42 | 11.22 | 1.80E-02 | At5g64200 | arginine/serine-rich splicing factor SC35 |
|  | 739 | | -0.56 | 10.33 | 1.80E-02 | At2g01870 | expressed protein |
|  | 740 | | -0.51 | 10.13 | 1.80E-02 | At5g58030 | SPP30 - like protein |
|  | 741 | | -0.73 | 12.04 | 1.81E-02 | At1g74730 | expressed protein |
|  | 742 | | 0.50 | 10.84 | 1.81E-02 | At4g17520 | nuclear RNA binding protein, putative |
|  | 743 | | -0.48 | 12.20 | 1.82E-02 | At1g51650 | epsilon subunit of mitochondrial F1-ATPase |
|  | 744 | | -0.46 | 11.38 | 1.82E-02 | At5g53330 | proline-rich cell wall protein-related |
|  | 745 | | -0.51 | 12.80 | 1.82E-02 | At5g17460 | expressed protein |
|  | 746 | | -0.46 | 13.58 | 1.83E-02 | At5g65430 | 14-3-3 protein GF14 kappa (grf8) |
|  | 747 | | -0.59 | 13.40 | 1.83E-02 | At4g26840 | ubiquitin-like protein (SMT3) |
|  | 748 | | -0.47 | 10.77 | 1.83E-02 | At5g13080 | WRKY family transcription factor |
|  | 749 | | -0.48 | 10.96 | 1.85E-02 | At4g37230 | photosystem II oxygen-evolving complex like protein |
|  | 750 | | 0.68 | 9.76 | 1.85E-02 | At2g45290 | transketolase precursor -related |
|  | 751 | | -0.42 | 11.49 | 1.85E-02 | At5g39340 | His-Asp Phosphotransfer Signal Transducer AHP3 |
|  | 752 | | 0.72 | 11.40 | 1.85E-02 | At1g15500 | adenine nucleotide translocase -related |
|  | 753 | | 0.39 | 12.34 | 1.85E-02 | At5g56380 | expressed protein |
|  | 754 | | 0.97 | 11.74 | 1.85E-02 | At2g24320 | hypothetical protein |
|  | 755 | | -0.66 | 10.87 | 1.85E-02 | At3g56040 | expressed protein |
|  | 756 | | 0.59 | 11.30 | 1.85E-02 | At1g80300 | adenine nucleotide translocase |
|  | 757 | | 0.91 | 10.51 | 1.85E-02 | At5g01240 | amino acid permease, putative |
|  | 758 | | 0.50 | 10.38 | 1.86E-02 | At5g58470 | RNA/ssDNA-binding protein - like |
|  | 759 | | -0.43 | 12.47 | 1.87E-02 | At5g56600 | profilin 5 |
|  | 760 | | 0.79 | 12.64 | 1.87E-02 | At1g05870 | expressed protein |
|  | 761 | | 0.52 | 10.96 | 1.87E-02 | At3g13460 | expressed protein |
|  | 762 | | 0.51 | 11.20 | 1.87E-02 | At5g61780 | 100 kDa coactivator - like protein |
|  | 763 | | -0.53 | 10.28 | 1.87E-02 | At1g12560 | expansin, putative (EXP7) |
|  | 764 | | 0.41 | 11.28 | 1.87E-02 | At3g22220 | expressed protein |
|  | 765 | | -0.44 | 10.91 | 1.87E-02 | At5g64180 | expressed protein |
|  | 766 | | -1.00 | 11.65 | 1.87E-02 | At5g35480 | expressed protein |
|  | 767 | | -0.72 | 10.11 | 1.87E-02 | At5g35860 | hypothetical protein |
|  | 768 | | 0.41 | 11.92 | 1.87E-02 | At3g16050 | ethylene-inducible protein -related |
|  | 769 | | 0.48 | 10.16 | 1.87E-02 | At3g18990 | transcriptional factor B3 family |
|  | 770 | | 0.53 | 10.37 | 1.87E-02 | At1g76810 | translation initiation factor eIF-2, putative |
|  | 771 | | -0.79 | 10.85 | 1.87E-02 | At5g43750 | expressed protein |
|  | 772 | | -0.51 | 14.60 | 1.87E-02 | At3g44010 | 40S ribosomal protein S29 (RPS29B) |
|  | 773 | | 0.48 | 10.79 | 1.87E-02 | At1g61255 | hypothetical protein |
|  | 774 | | 0.50 | 10.10 | 1.87E-02 | At1g80480 | expressed protein |
|  | 775 | | -0.74 | 12.88 | 1.88E-02 | At1g01170 | expressed protein |
|  | 776 | | 0.56 | 9.35 | 1.90E-02 | At1g13980 | pattern formation protein EMB30 |
|  | 777 | | -0.45 | 11.57 | 1.90E-02 | At5g27560 | expressed protein |
|  | 778 | | -0.50 | 9.60 | 1.91E-02 | At5g50530 | CBS domain containing protein |
|  | 779 | | 0.78 | 11.54 | 1.91E-02 | At5g05170 | cellulose synthase, catalytic subunit (Ath-B) |
|  | 780 | | 0.41 | 10.52 | 1.91E-02 | At5g42390 | pitrilysin |
|  | 781 | | -0.79 | 9.82 | 1.91E-02 | At3g49110 | peroxidase |
|  | 782 | | 0.76 | 11.69 | 1.92E-02 | At5g60480 | hypothetical protein |
|  | 783 | | 0.89 | 11.72 | 1.92E-02 | At1g30795 | proline-rich protein family |
|  | 784 | | -0.55 | 12.10 | 1.92E-02 | At4g14020 | expressed protein |
|  | 785 | | 0.39 | 13.79 | 1.92E-02 | At5g04740 | EST |
|  | 786 | | **1.64** | **12.47** | **1.92E-02** | **At4g14090** | **UDP-glycosyltransferase family** |
|  | 787 | | 0.37 | 10.57 | 1.92E-02 | At2g29080 | AAA-type ATPase -related |
|  | 788 | | 0.41 | 10.02 | 1.92E-02 | At1g04990 | zinc finger protein 2 -related |
|  | 789 | | -0.43 | 11.19 | 1.92E-02 | At3g25220 | immunophilin / FKBP-type peptidyl-prolyl cis-trans isomerase (FKBP15-1) |
|  | 790 | | 0.52 | 11.46 | 1.92E-02 | At3g21690 | MATE efflux protein family |
|  | 791 | | 0.60 | 9.71 | 1.92E-02 | At5g51300 | splicing factor 1 (SF1), putative |
|  | 792 | | 0.43 | 12.32 | 1.93E-02 | At1g62170 | serpin family |
|  | 793 | | 0.74 | 10.06 | 1.93E-02 | At3g09350 | expressed protein |
|  | 794 | | -0.83 | 10.64 | 1.94E-02 | At1g12010 | 1-aminocyclopropane-1-carboxylate oxidase (ACC oxidase), putative |
|  | 795 | | 0.63 | 12.66 | 1.94E-02 | At3g09840 | transitional endoplasmic reticulum ATPase -related |
|  | 796 | | -0.43 | 11.03 | 1.94E-02 | At5g50580 | SUMO activating enzyme 1b (SAE1b) |
|  | 797 | | 0.44 | 14.93 | 1.94E-02 | At5g01560 | receptor lectin kinase, putative |
|  | 798 | | -1.08 | 10.31 | 1.94E-02 | At2g37750 | expressed protein |
|  | 799 | | 0.48 | 10.66 | 1.96E-02 | At4g38510 | probable H+transporting ATPase |
|  | 800 | | 0.48 | 10.02 | 1.96E-02 | At5g49945 | expressed protein |
|  | 801 | | -0.40 | 13.12 | 1.96E-02 | At5g67590 | expressed protein |
|  | 802 | | -0.53 | 9.39 | 1.96E-02 | At2g26180 | SF16 protein {Helianthus annuus} -related |
|  | 803 | | 0.65 | 10.19 | 1.96E-02 | At4g26430 | COP9 signalosome subunit 6 (CSN6B), CSN complex subunit 6B |
|  | 804 | | -0.54 | 10.78 | 1.96E-02 | At3g51500 | expressed protein |
|  | 805 | | 0.53 | 9.74 | 1.97E-02 | At4g01530 | reverse transcriptase protein -related |
|  | 806 | | -0.80 | 14.80 | 1.97E-02 | At2g25510 | expressed protein |
|  | 807 | | 0.87 | 10.18 | 1.97E-02 | At1g62440 | leucine-rich repeat extensin family |
|  | 808 | | -0.52 | 12.68 | 1.97E-02 | At3g11500 | small nuclear ribonucleo protein polypeptide G -related |
|  | 809 | | 0.54 | 10.41 | 1.99E-02 | At5g57710 | Arabidopsis thaliana genomic DNA, chromosome 5, P1 clone:MRI1 |
|  | 810 | | 1.02 | 11.90 | 1.99E-02 | At1g26250 | proline-rich extensin, putative |
|  | 811 | | -0.77 | 13.42 | 1.99E-02 | At5g40370 | glutaredoxin, putative |
|  | 812 | | -0.37 | 10.39 | 2.00E-02 | At1g19650 | sec14 cytosolic factor -related |
|  | 813 | | -0.66 | 11.40 | 2.00E-02 | At2g40110 | expressed protein |
|  | 814 | | 0.71 | 11.80 | 2.00E-02 | At5g56330 | proline-rich protein family |
|  | 815 | | -0.54 | 9.34 | 2.01E-02 | At5g25350 | ESTs |
|  | 816 | | 0.54 | 9.88 | 2.01E-02 | At3g62980 | transport inhibitor response 1 (TIR1), AtFBL1 |
|  | 817 | | 0.91 | 14.49 | 2.01E-02 | At5g02500 | heat shock protein hsc70-1 (hsp70-1) (hsc70.1) |
|  | 818 | | 0.49 | 9.53 | 2.01E-02 | At5g24710 | expressed protein |
|  | 819 | | 0.48 | 12.38 | 2.01E-02 | At5g16110 | expressed protein |
|  | 820 | | 0.69 | 14.76 | 2.02E-02 | At1g05130 | Arabidopsis thaliana chromosome 1 YAC yUP8H12 complete sequence |
|  | 821 | | 0.79 | 10.46 | 2.02E-02 | At5g12350 | expressed protein |
|  | 822 | | -0.64 | 14.33 | 2.02E-02 | At2g41100 | calmodulin-related protein 3, touch-induced (TCH3) |
|  | 823 | | 0.45 | 11.18 | 2.02E-02 | At5g62000 | auxin response factor - like protein |
|  | 824 | | 0.64 | 10.52 | 2.02E-02 | At1g58220 | Myb family protein |
|  | 825 | | -0.48 | 9.91 | 2.03E-02 | At2g27180 | expressed protein |
|  | 826 | | 0.65 | 13.86 | 2.03E-02 | At2g48080 | oxidoreductase, 2OG-Fe(II) oxygenase family |
|  | 827 | | -0.53 | 10.78 | 2.03E-02 | At3g46560 | small zinc finger-related protein TIM9 |
|  | 828 | | 0.43 | 9.62 | 2.04E-02 | At5g02310 | eceriferum3 (CER3) |
|  | 829 | | 0.77 | 10.05 | 2.04E-02 | At5g21170 | AMPKBI (5'-AMP-activated protein kinase, beta subunit, complex-interacting region) domain family |
|  | 830 | | -0.58 | 12.30 | 2.04E-02 | At4g30810 | serine carboxypeptidase -related |
|  | 831 | | 0.50 | 14.52 | 2.04E-02 | At1g22480 | plastocyanin-like domain containing protein |
|  | 832 | | 0.58 | 9.61 | 2.05E-02 | At1g68790 | nuclear matrix constituent protein 1 (NMCP1) -related |
|  | 833 | | -0.45 | 11.92 | 2.06E-02 | At3g02770 | dimethylmenaquinone methyltransferase family |
|  | 834 | | 0.72 | 9.28 | 2.06E-02 | At4g09820 | bHLH protein |
|  | 835 | | -0.65 | 11.70 | 2.06E-02 | At1g76450 | oxygen-evolving complex-23 related protein |
|  | 836 | | -0.58 | 12.78 | 2.06E-02 | At5g59613 | Arabidopsis thaliana F18L15.150 mRNA, complete cds |
|  | 837 | | 0.88 | 12.42 | 2.06E-02 | At4g30260 | expressed protein |
|  | 838 | | -0.46 | 12.21 | 2.06E-02 | At4g12880 | plastocyanin-like domain containing protein |
|  | 839 | | 0.54 | 11.21 | 2.06E-02 | At3g29320 | glucan phosphorylase, putative |
|  | 840 | | -0.48 | 12.28 | 2.06E-02 | At4g29670 | thioredoxin family |
|  | 841 | | 0.67 | 9.13 | 2.07E-02 | At4g02510 | chloroplast outer envelope 86 protein -related |
|  | 842 | | -0.44 | 8.87 | 2.07E-02 | At1g71015 | hypothetical protein |
|  | 843 | | -0.53 | 9.99 | 2.07E-02 | At3g50770 | calmodulin-related protein, putative |
|  | 844 | | -0.79 | 12.35 | 2.08E-02 | At2g05620 | expressed protein |
|  | 845 | | 0.54 | 9.93 | 2.08E-02 | At4g39740 | expressed protein |
|  | 846 | | -0.44 | 11.43 | 2.08E-02 | At1g56600 | galactinol synthase, putative |
|  | 847 | | -0.53 | 9.96 | 2.08E-02 | At3g43720 | protease inhibitor/seed storage/lipid transfer protein (LTP) family |
|  | 848 | | 0.68 | 11.73 | 2.08E-02 | At5g49910 | heat shock protein cpHsc70-2 (hsc70-7) |
|  | 849 | | 0.47 | 10.94 | 2.08E-02 | At1g76070 | expressed protein |
|  | 850 | | -0.52 | 11.30 | 2.09E-02 | At1g34560 | hypothetical protein |
|  | 851 | | -0.70 | 13.06 | 2.09E-02 | At1g64750 | expressed protein |
|  | 852 | | 0.51 | 12.49 | 2.09E-02 | At2g18020 | A.thaliana mRNA for ribosomal protein L2 |
|  | 853 | | -0.50 | 9.85 | 2.09E-02 | At2g29720 | monooxygenase family |
|  | 854 | | 0.73 | 11.27 | 2.09E-02 | At3g12410 | hypothetical protein |
|  | 855 | | 0.44 | 11.55 | 2.09E-02 | At3g11710 | lysyl-tRNA synthetase, putative |
|  | 856 | | -0.65 | 10.46 | 2.10E-02 | At3g01440 | PsbQ domain protein family, putative |
|  | 857 | | -0.55 | 9.36 | 2.10E-02 | At3g08890 | expressed protein |
|  | 858 | | -0.53 | 14.65 | 2.10E-02 | At1g52230 | photosystem I subunit VI precursor |
|  | 859 | | -0.52 | 12.80 | 2.10E-02 | At4g26850 | expressed protein |
|  | 860 | | 0.39 | 11.27 | 2.11E-02 | At3g59380 | farnesyltransferase alpha subunit (FTA/protein farnesyltransferase), putative |
|  | 861 | | -0.71 | 13.51 | 2.11E-02 | At5g04980 | endonuclease/exonuclease/phosphatase family |
|  | 862 | | 0.54 | 11.82 | 2.11E-02 | At5g06010 | hypothetical protein |
|  | 863 | | 0.56 | 10.68 | 2.11E-02 | At5g25100 | endomembrane protein 70, putative |
|  | 864 | | -0.67 | 10.54 | 2.11E-02 | At3g12920 | expressed protein |
|  | 865 | | -0.83 | 10.99 | 2.11E-02 | At5g48490 | protease inhibitor/seed storage/lipid transfer protein (LTP) family |
|  | 866 | | -0.76 | 9.26 | 2.11E-02 | At3g12710 | expressed protein |
|  | 867 | | 0.50 | 12.63 | 2.11E-02 | At1g49240 | actin 8 |
|  | 868 | | 0.92 | 11.72 | 2.11E-02 | At5g26330 | plastocyanin-like domain containing protein (mavicyanin, putative) |
|  | 869 | | 0.54 | 10.48 | 2.11E-02 | At3g57650 | acyl-CoA:1-acylglycerol-3-phosphate acyltransferase, putative |
|  | 870 | | 0.52 | 9.60 | 2.11E-02 | At5g60160 | aspartyl aminopeptidase - like protein |
|  | 871 | | -0.45 | 11.51 | 2.11E-02 | At1g03330 | expressed protein |
|  | 872 | | 0.45 | 9.46 | 2.11E-02 | At4g09020 | glycoside hydrolase family 13 |
|  | 873 | | 0.46 | 10.77 | 2.11E-02 | At5g35170 | adenylate kinase -related protein |
|  | 874 | | 0.80 | 12.31 | 2.11E-02 | At1g33240 | DNA-binding factor -related |
|  | 875 | | -0.48 | 9.53 | 2.13E-02 | At3g45290 | seven transmembrane MLO protein family (MLO3) |
|  | 876 | | -0.42 | 9.54 | 2.13E-02 | At1g55910 | metal transporter, putative (ZIP11) |
|  | 877 | | -0.58 | 11.60 | 2.13E-02 | At4g27230 | histone H2A, putative |
|  | 878 | | -0.82 | 11.73 | 2.13E-02 | At5g66490 | expressed protein |
|  | 879 | | -0.62 | 10.24 | 2.13E-02 | At1g22430 | alcohol dehydrogenase (ADH), putative |
|  | 880 | | -0.81 | 11.08 | 2.13E-02 | At2g34860 | expressed protein |
|  | 881 | | -0.41 | 10.05 | 2.13E-02 | At4g01060 | Arabidopsis thaliana DNA chromosome 4, contig fragment No. 3 |
|  | 882 | | -0.97 | 13.80 | 2.13E-02 | At2g41090 | calmodulin-like calcium binding protein (CaBP-22) |
|  | 883 | | -0.52 | 11.83 | 2.13E-02 | At2g01090 | ubiquinol-cytochrome c reductase -related |
|  | 884 | | 0.45 | 10.83 | 2.13E-02 | At2g03820 | nonsense-mediated mRNA decay protein -related |
|  | 885 | | -0.61 | 14.06 | 2.13E-02 | At5g12140 | cystatin (emb|CAA03929.1) |
|  | 886 | | -0.63 | 10.00 | 2.13E-02 | At1g34640 | expressed protein |
|  | 887 | | 0.76 | 11.26 | 2.13E-02 | At5g66220 | chalcone-flavanone isomerase (chalcone isomerase) (CHI), putative |
|  | 888 | | -0.55 | 12.09 | 2.13E-02 | At5g43970 | expressed protein |
|  | 889 | | 0.68 | 11.22 | 2.13E-02 | At2g01480 | axi 1 protein from Nicotiana tabacum -related |
|  | 890 | | -0.58 | 9.54 | 2.13E-02 | At1g66490 | F-box protein family |
|  | 891 | | -0.41 | 11.36 | 2.13E-02 | At1g63460 | glutathione peroxidase, putative |
|  | 892 | | 0.60 | 10.78 | 2.14E-02 | At3g15880 | WD-repeat protein -related |
|  | 893 | | -0.51 | 10.10 | 2.14E-02 | At5g51510 | expressed protein |
|  | 894 | | 0.71 | 10.41 | 2.15E-02 | At3g03710 | polyribonucleotide nucleotidyltransferase, putative |
|  | 895 | | 0.44 | 12.40 | 2.16E-02 | At1g52040 | jacalin lectin family |
|  | 896 | | 0.67 | 11.52 | 2.16E-02 | At3g46230 | class I heat shock protein(HSP 17.4) |
|  | 897 | | 0.49 | 9.93 | 2.16E-02 | At5g16150 | hexose transporter, putative |
|  | 898 | | -0.47 | 15.43 | 2.16E-02 | At1g54410 | dehydrin protein family |
|  | 899 | | -0.87 | 10.85 | 2.17E-02 | At1g66400 | calmodulin-related protein, putative |
|  | 900 | | -0.44 | 10.06 | 2.17E-02 | At5g02280 | expressed protein |
|  | 901 | | -0.67 | 10.84 | 2.18E-02 | At2g29180 | expressed protein |
|  | 902 | | -0.43 | 10.45 | 2.18E-02 | At5g11650 | hydrolase, alpha/beta fold family |
|  | 903 | | -0.51 | 11.21 | 2.19E-02 | At1g25550 | expressed protein |
|  | 904 | | 0.53 | 10.88 | 2.20E-02 | At1g52030 | myrosinase binding protein, putative |
|  | 905 | | -0.43 | 12.76 | 2.20E-02 | At5g17870 | plastid-specific ribosomal protein 6 precursor (Psrp-6) - like |
|  | 906 | | -0.43 | 10.06 | 2.20E-02 | At3g05070 | expressed protein |
|  | 907 | | -0.36 | 12.05 | 2.20E-02 | At4g30220 | snRNP Sm protein F - like |
|  | 908 | | -0.54 | 10.21 | 2.20E-02 | At5g49670 | hypothetical protein |
|  | 909 | | -0.64 | 13.08 | 2.20E-02 | At1g21550 | calcium-binding protein, putative |
|  | 910 | | 0.65 | 12.15 | 2.20E-02 | At5g54160 | O-methyltransferase 1 |
|  | 911 | | -0.41 | 12.55 | 2.20E-02 | At3g18300 | hypothetical protein |
|  | 912 | | -0.53 | 8.82 | 2.20E-02 | At3g28280 | hypothetical protein |
|  | 913 | | -0.76 | 10.36 | 2.20E-02 | At2g11810 | 1,2-diacylglycerol 3-beta-galactosyltransferase (UDP-galactose:diacylglycerol galactosyltransferase) |
|  | 914 | | 0.44 | 11.93 | 2.20E-02 | At5g67500 | porin-related protein |
|  | 915 | | 0.46 | 11.63 | 2.20E-02 | At4g32210 | expressed protein |
|  | 916 | | 0.43 | 10.61 | 2.21E-02 | At5g26742 | DEAD box RNA helicase (RH3) |
|  | 917 | | 0.66 | 10.51 | 2.21E-02 | At5g03610 | GDSL-motif lipase/hydrolase protein |
|  | 918 | | 0.42 | 9.14 | 2.21E-02 | At1g27340 | F-box protein family |
|  | 919 | | 0.54 | 10.19 | 2.22E-02 | At5g26360 | Arabidopsis thaliana unknown protein (F9D12.18/AT5g26360) mRNA, complete cds |
|  | 920 | | 0.49 | 12.04 | 2.22E-02 | At4g25130 | protein-methionine-S-oxide reductase |
|  | 921 | | -0.42 | 10.96 | 2.23E-02 | At1g63800 | ubiquitin-conjugating enzyme 5 (UBC5) |
|  | 922 | | -0.69 | 11.45 | 2.23E-02 | At4g22080 | polysaccharide lyase family 1 (pectate lyase) |
|  | 923 | | 0.37 | 9.58 | 2.23E-02 | At1g09570 | phytochrome A (PHYA) |
|  | 924 | | 0.79 | 10.02 | 2.23E-02 | At5g41370 | TFIIH basal transcription factor complex helicase XPB subunit, putative |
|  | 925 | | 0.88 | 10.87 | 2.24E-02 | At2g35110 | expressed protein |
|  | 926 | | -0.42 | 11.23 | 2.24E-02 | At4g11370 | RING-H2 finger protein, putative |
|  | 927 | | -0.54 | 11.74 | 2.24E-02 | At5g51010 | expressed protein |
|  | 928 | | 0.63 | 12.32 | 2.24E-02 | At3g01700 | arabinogalactan-protein (AGP11) |
|  | 929 | | 0.52 | 9.87 | 2.26E-02 | At5g44800 | chromodomain-helicase-DNA-binding (CHD) protein family |
|  | 930 | | 0.63 | 9.19 | 2.26E-02 | At1g65060 | 4-coumarate:CoA ligase 3 (4-coumaroyl-CoA synthase 3) (4CL3), putative |
|  | 931 | | 0.53 | 9.76 | 2.26E-02 | At4g39840 | expressed protein |
|  | 932 | | -0.52 | 10.51 | 2.26E-02 | At3g07170 | expressed protein |
|  | 933 | | 0.74 | 11.00 | 2.26E-02 | At3g62640 | expressed protein |
|  | 934 | | 0.40 | 12.67 | 2.26E-02 | At4g10540 | Arabidopsis thaliana BAC F3H7 |
|  | 935 | | -0.44 | 10.66 | 2.27E-02 | At5g62610 | bHLH protein family |
|  | 936 | | 1.18 | 10.18 | 2.30E-02 | At1g74310 | heat shock protein 101 (HSP101) |
|  | 937 | | -0.54 | 11.71 | 2.31E-02 | At4g18040 | translation initiation factor eIF4E |
|  | 938 | | -0.52 | 9.92 | 2.31E-02 | At5g26130 | pathogenesis-related protein, putative |
|  | 939 | | -0.51 | 10.80 | 2.31E-02 | At3g11730 | GTP-binding protein, putative |
|  | 940 | | 0.82 | 10.00 | 2.31E-02 | At1g23020 | ferric-chelate reductase, putative |
|  | 941 | | -0.44 | 9.57 | 2.32E-02 | At1g08180 | hypothetical protein |
|  | 942 | | -0.52 | 10.50 | 2.32E-02 | At3g25600 | calmodulin, putative |
|  | 943 | | -0.42 | 9.75 | 2.32E-02 | At2g17280 | expressed protein |
|  | 944 | | -0.42 | 13.83 | 2.33E-02 | At5g42300 | ubiquitin family |
|  | 945 | | 0.60 | 10.42 | 2.33E-02 | At5g64310 | arabinogalactan-protein (AGP1) |
|  | 946 | | 0.73 | 11.75 | 2.33E-02 | At5g49910 | heat shock protein cpHsc70-2 (hsc70-7) |
|  | 947 | | 0.35 | 11.71 | 2.33E-02 | At1g68000 | CDP-diacylglycerol--inositol 3-phosphatidyltransferase (phosphatidylinositol synthase) (PIS1) |
|  | 948 | | -0.50 | 11.21 | 2.33E-02 | At5g13410 | immunophilin / FKBP-type peptidyl-prolyl cis-trans isomerase |
|  | 949 | | -0.66 | 11.08 | 2.33E-02 | At5g09960 | expressed protein |
|  | 950 | | 0.47 | 9.92 | 2.35E-02 | At5g18200 | galactose-1-phosphate uridyl transferase-related protein |
|  | 951 | | -0.75 | 9.71 | 2.35E-02 | At1g29500 | auxin-induced (indole-3-acetic acid induced) protein, putative |
|  | 952 | | -0.52 | 10.61 | 2.36E-02 | At5g12390 | expressed protein |
|  | 953 | | -0.51 | 9.58 | 2.36E-02 | At3g48550 | expressed protein |
|  | 954 | | 0.54 | 13.15 | 2.37E-02 | At3g08760 | protein kinase family |
|  | 955 | | 0.85 | 11.73 | 2.37E-02 | At1g71890 | sucrose transporter (sucrose-proton symporter) |
|  | 956 | | -0.98 | 10.44 | 2.37E-02 | At5g52730 | heavy-metal-associated domain-containing protein |
|  | 957 | | 0.48 | 11.86 | 2.37E-02 | At3g51440 | strictosidine synthase-related |
|  | 958 | | -0.51 | 9.57 | 2.37E-02 | At5g16200 | hypothetical protein |
|  | 959 | | 0.45 | 11.68 | 2.37E-02 | At3g52960 | peroxiredoxin - like protein |
|  | 960 | | -0.47 | 11.55 | 2.37E-02 | At5g13320 | auxin-responsive - like protein |
|  | 961 | | -0.60 | 14.14 | 2.37E-02 | At3g56800 | calmodulin |
|  | 962 | | -0.57 | 11.14 | 2.37E-02 | At4g14410 | bHLH protein |
|  | 963 | | -0.47 | 12.30 | 2.38E-02 | At3g53990 | EST, Highly similar to T45939 hypothetical protein F5K20.290 - Arabidopsis thaliana [A.thaliana] |
|  | 964 | | -0.44 | 10.51 | 2.38E-02 | At2g43640 | signal recognition particle protein 14kD, ATSRP14 -related |
|  | 965 | | 0.48 | 10.42 | 2.40E-02 | At3g01560 | proline-rich protein family |
|  | 966 | | 0.56 | 11.55 | 2.40E-02 | At2g20580 | 26S proteasome regulatory subunit S2 (RPN1) |
|  | 967 | | 0.55 | 9.67 | 2.40E-02 | At4g39210 | glucose-1-phosphate adenylyltransferase, large subunit 3 (ADP-glucose pyrophosphorylase) (APL3) |
|  | 968 | | -0.44 | 12.64 | 2.40E-02 | At1g26550 | expressed protein |
|  | 969 | | -0.57 | 10.33 | 2.40E-02 | At5g48540 | 33 kDa secretory protein-related |
|  | 970 | | -0.53 | 10.85 | 2.40E-02 | At1g77870 | expressed protein |
|  | 971 | | -0.38 | 11.59 | 2.41E-02 | At4g10100 | auxin-regulated protein |
|  | 972 | | 0.70 | 11.99 | 2.41E-02 | At1g80160 | glyoxalase family protein (lactoylglutathione lyase family protein) |
|  | 973 | | -0.46 | 12.89 | 2.41E-02 | At3g46320 | histone H4 |
|  | 974 | | -0.53 | 9.23 | 2.41E-02 | At1g35513 | Genomic sequence for Arabidopsis thaliana BAC F15O4 from chromosome I |
|  | 975 | | 0.49 | 11.10 | 2.41E-02 | At3g25800 | serine/threonine protein phosphatase 2A, 65 KDa regulatory subunit A |
|  | 976 | | -0.54 | 9.21 | 2.41E-02 | At3g09890 | expressed protein |
|  | 977 | | -0.62 | 12.83 | 2.42E-02 | At1g76080 | thioredoxin family |
|  | 978 | | 0.49 | 9.70 | 2.42E-02 | At1g17440 | expressed protein |
|  | 979 | | -0.58 | 9.17 | 2.43E-02 | At1g13540 | expressed protein |
|  | 980 | | -0.35 | 11.48 | 2.43E-02 | At5g65310 | homeobox-leucine zipper protein ATHB-5 (HD-Zip protein ATHB-5) |
|  | 981 | | -0.48 | 10.33 | 2.43E-02 | At4g15680 | glutaredoxin protein family |
|  | 982 | | 0.47 | 9.55 | 2.45E-02 | At1g78880 | expressed protein |
|  | 983 | | -0.54 | 10.12 | 2.46E-02 | At3g02800 | expressed protein |
|  | 984 | | 0.44 | 11.43 | 2.46E-02 | At5g23580 | calcium-dependent protein kinase (CDPK) |
|  | 985 | | -0.65 | 12.82 | 2.48E-02 | At2g29700 | expressed protein |
|  | 986 | | -0.56 | 10.27 | 2.49E-02 | At4g05340 | hypothetical protein |
|  | 987 | | 0.37 | 9.21 | 2.50E-02 | At1g34360 | Arabidopsis thaliana chromosome 1 BAC F7P12 genomic sequence |
|  | 988 | | 0.74 | 10.54 | 2.50E-02 | At5g67130 | ESTs |
|  | 989 | | -0.48 | 12.42 | 2.51E-02 | At4g30010 | expressed protein |
|  | 990 | | -0.52 | 10.19 | 2.51E-02 | At3g43740 | leucine rich repeat protein family |
|  | 991 | | 0.87 | 11.45 | 2.51E-02 |  | Genomic sequence for Arabidopsis thaliana BAC F5O11 from chromosome I |
|  | 992 | | -0.74 | 10.55 | 2.51E-02 | At5g39220 | hydrolase, alpha/beta fold family |
|  | 993 | | -0.36 | 13.40 | 2.52E-02 | At4g28060 | hypothetical protein |
|  | 994 | | 0.58 | 9.83 | 2.52E-02 | At4g12290 | copper amine oxidase -related protein |
|  | 995 | | -0.48 | 14.70 | 2.53E-02 | At2g43530 | trypsin inhibitor -related |
|  | 996 | | 0.49 | 10.65 | 2.53E-02 | At2g30110 | ubiquitin activating enzyme 1 (UBA1) |
|  | 997 | | -0.40 | 11.84 | 2.53E-02 | At2g32720 | cytochrome b5, putative |
|  | 998 | | 0.57 | 11.75 | 2.54E-02 | At4g25860 | hypothetical protein |
|  | 999 | | 0.43 | 10.20 | 2.55E-02 | At5g24760 | alcohol dehydrogenase (ADH), putative |
|  | 1000 | | -0.44 | 10.10 | 2.56E-02 | At2g44360 | expressed protein |
|  | 1001 | | 0.45 | 9.47 | 2.57E-02 | At5g01850 | protein kinase, putative |
|  | 1002 | | -0.59 | 9.49 | 2.58E-02 | At5g23460 | expressed protein |
|  | 1003 | | 0.61 | 11.60 | 2.58E-02 | At5g41300 | hypothetical protein |
|  | 1004 | | 0.71 | 11.05 | 2.58E-02 | At1g68560 | glycosyl hydrolase family 31 (alpha-xylosidase) |
|  | 1005 | | 0.62 | 12.24 | 2.58E-02 | At2g44350 | citrate synthase |
|  | 1006 | | -0.56 | 9.62 | 2.59E-02 | At3g05000 | expressed protein |
|  | 1007 | | -0.37 | 13.62 | 2.59E-02 | At4g02770 | photosystem I reaction center subunit II precursor -related |
|  | 1008 | | 0.65 | 10.18 | 2.59E-02 | At3g07660 | expressed protein |
|  | 1009 | | -0.62 | 10.15 | 2.59E-02 | At4g20030 | RNA recognition motif (RRM)-containing protein |
|  | 1010 | | 0.73 | 11.84 | 2.61E-02 | At5g58250 | unknown protein (sp|P72777) -related |
|  | 1011 | | -0.51 | 10.32 | 2.62E-02 | At1g47830 | clathrin coat assembly protein AP17 -related |
|  | 1012 | | 0.50 | 10.15 | 2.62E-02 | At1g10270 | pentatricopeptide (PPR) repeat-containing protein |
|  | 1013 | | -0.40 | 9.05 | 2.62E-02 | At1g23470 | Arabidopsis thaliana chromosome 1 BAC F5O8 sequence |
|  | 1014 | | 0.68 | 11.63 | 2.62E-02 | At5g49910 | heat shock protein cpHsc70-2 (hsc70-7) |
|  | 1015 | | 0.60 | 11.92 | 2.62E-02 | At1g28660 | lipase, putative |
|  | 1016 | | 0.43 | 9.82 | 2.63E-02 | At1g70940 | auxin transport protein, putative (PIN3) |
|  | 1017 | | -0.46 | 12.65 | 2.63E-02 | At4g25740 | 40S ribosomal protein S10 (RPS10A) |
|  | 1018 | | -0.48 | 12.94 | 2.63E-02 | At5g62750 | expressed protein |
|  | 1019 | | 0.51 | 12.51 | 2.63E-02 | At2g20090 | hypothetical protein |
|  | 1020 | | 0.48 | 11.77 | 2.64E-02 | At4g24660 | expressed protein |
|  | 1021 | | 0.50 | 10.74 | 2.64E-02 | At4g26190 | expressed protein |
|  | 1022 | | -0.41 | 10.45 | 2.64E-02 | At4g04490 | receptor-related protein kinase |
|  | 1023 | | -0.66 | 12.09 | 2.64E-02 | At5g01350 | expressed protein |
|  | 1024 | | 0.38 | 8.91 | 2.64E-02 | At4g17110 | hypothetical protein |
|  | 1025 | | 0.52 | 12.28 | 2.64E-02 | At5g16050 | 14-3-3 protein GF14 upsilon (grf5) |
|  | 1026 | | 0.53 | 15.08 | 2.64E-02 | At3g44310 | nitrilase 1 |
|  | 1027 | | 0.61 | 10.31 | 2.64E-02 | At1g30360 | ERD4 protein |
|  | 1028 | | 0.63 | 9.62 | 2.65E-02 | At1g15780 | expressed protein |
|  | 1029 | | -0.45 | 10.87 | 2.65E-02 | At4g04330 | expressed protein |
|  | 1030 | | -0.43 | 9.23 | 2.65E-02 | At5g51370 | F-box protein family |
|  | 1031 | | -0.52 | 11.95 | 2.65E-02 | At5g40780 | lysine and histidine specific transporter, putative |
|  | 1032 | | -0.46 | 10.04 | 2.65E-02 |  | Genomic sequence for Arabidopsis thaliana BAC F15O4 from chromosome I |
|  | 1033 | | -0.58 | 9.95 | 2.65E-02 | At2g02500 | Arabidopsis thaliana chromosome II section 10 of 255 of the complete sequence. |
|  | 1034 | | -0.91 | 14.44 | 2.67E-02 | At2g15960 | expressed protein |
|  | 1035 | | 0.61 | 8.83 | 2.67E-02 | At3g45950 | hypothetical protein |
|  | 1036 | | 0.50 | 8.99 | 2.67E-02 | At1g79280 | expressed protein |
|  | 1037 | | 0.39 | 11.15 | 2.68E-02 | At5g58130 | RNA recognition motif (RRM)-containing protein |
|  | 1038 | | 0.54 | 9.22 | 2.69E-02 | At3g44080 | F-box protein family |
|  | 1039 | | -0.38 | 11.98 | 2.69E-02 | At3g44100 | expressed protein |
|  | 1040 | | 0.66 | 10.41 | 2.69E-02 | At1g30680 | expressed protein |
|  | 1041 | | -0.41 | 11.61 | 2.71E-02 | At1g53400 | expressed protein |
|  | 1042 | | 0.52 | 10.01 | 2.71E-02 | At5g62640 | proline-rich protein family |
|  | 1043 | | 0.93 | 11.55 | 2.76E-02 | At5g14920 | expressed protein |
|  | 1044 | | 0.66 | 10.86 | 2.76E-02 | At5g27120 | SAR DNA-binding protein, putative |
|  | 1045 | | 0.59 | 11.11 | 2.77E-02 | At3g22330 | DEAD box RNA helicase, putative |
|  | 1046 | | 0.59 | 10.70 | 2.77E-02 | At5g11520 | aspartate aminotransferase, chloroplast (transaminase A/Asp3) |
|  | 1047 | | -0.38 | 11.23 | 2.78E-02 | At5g40930 | protein import receptor TOM20, mitochondrial-related |
|  | 1048 | | -0.83 | 9.65 | 2.79E-02 | At1g78510 | geranyl diphosphate synthase (GPPS)(dimethylallyltransferase), putative |
|  | 1049 | | 0.40 | 10.13 | 2.79E-02 | At2g39260 | middle domain of eukaryotic initiation factor 4G (MIF4G) domain-containing protein |
|  | 1050 | | 0.74 | 11.47 | 2.79E-02 | At3g17510 | CBL-interacting protein kinase 1 |
|  | 1051 | | 0.60 | 11.97 | 2.79E-02 | At4g09030 | arabinogalactan-protein (AGP10) |
|  | 1052 | | -0.46 | 9.48 | 2.79E-02 | At1g69160 | expressed protein |
|  | 1053 | | -0.66 | 10.24 | 2.79E-02 | At3g29170 | expressed protein |
|  | 1054 | | -0.72 | 10.96 | 2.79E-02 | At5g43700 | auxin-responsive protein IAA4 (Indoleacetic acid-induced protein 4) (Auxin-induced protein AUX2-11) |
|  | 1055 | | 0.48 | 10.60 | 2.79E-02 | At2g13560 | malate oxidoreductase (malic enzyme), putative |
|  | 1056 | | 0.97 | 11.44 | 2.79E-02 | At1g17810 | major intrinsic protein (MIP) family |
|  | 1057 | | 0.36 | 12.52 | 2.79E-02 | At1g79950 | expressed protein |
|  | 1058 | | -0.58 | 10.03 | 2.80E-02 | At3g61210 | expressed protein |
|  | 1059 | | 0.46 | 11.37 | 2.82E-02 | At3g06720 | importin alpha subunit |
|  | 1060 | | 0.37 | 9.58 | 2.82E-02 | At1g51140 | bHLH protein family |
|  | 1061 | | 0.42 | 9.14 | 2.83E-02 | At4g32720 | RNA recognition motif (RRM)-containing protein |
|  | 1062 | | -0.54 | 8.92 | 2.85E-02 | At1g69940 | Arabidopsis thaliana chromosome 1 BAC T17F3 genomic sequence |
|  | 1063 | | 0.63 | 15.10 | 2.85E-02 | At3g22070 | proline-rich protein family |
|  | 1064 | | -0.42 | 13.81 | 2.86E-02 | At5g42300 | ubiquitin family |
|  | 1065 | | 0.71 | 9.65 | 2.86E-02 | At1g62020 | coatomer alpha subunit -related |
|  | 1066 | | 0.43 | 10.27 | 2.86E-02 | At2g02160 | expressed protein |
|  | 1067 | | 0.73 | 10.60 | 2.86E-02 | At4g19620 | hypothetical protein |
|  | 1068 | | 0.54 | 12.03 | 2.86E-02 | At1g49420 | heavy-metal-associated domain-containing protein |
|  | 1069 | | -0.50 | 9.18 | 2.86E-02 | At2g21650 | myb family transcription factor |
|  | 1070 | | 0.43 | 9.14 | 2.86E-02 | At5g64570 | glycosyl hydrolase family 3 |
|  | 1071 | | 0.54 | 9.28 | 2.86E-02 | At2g25140 | HSP100/ClpB, putative |
|  | 1072 | | 0.52 | 10.17 | 2.87E-02 | At4g38550 | Arabidopsis thaliana DNA chromosome 4, BAC clone F20M13 (ESSA project) |
|  | 1073 | | 0.59 | 10.95 | 2.87E-02 | At5g57010 | expressed protein |
|  | 1074 | | 0.38 | 10.14 | 2.87E-02 | At3g43300 | guanine nucleotide exchange protein family |
|  | 1075 | | -0.77 | 11.46 | 2.88E-02 | At4g05590 | expressed protein |
|  | 1076 | | -0.54 | 9.85 | 2.88E-02 | At5g66550 | Arabidopsis thaliana genomic DNA, chromosome 5, TAC clone:K1F13 |
|  | 1077 | | 0.57 | 11.77 | 2.88E-02 | At1g20260 | Sequence of BAC F14O10 from Arabidopsis thaliana chromosome 1 |
|  | 1078 | | 0.53 | 9.18 | 2.88E-02 | At4g00900 | calcium-transporting ATPase 2, endoplasmic reticulum-type (calcium pump) |
|  | 1079 | | 0.69 | 10.53 | 2.88E-02 | At3g18440 | EST |
|  | 1080 | | 0.69 | 10.81 | 2.89E-02 | At1g79560 | FtsH protease, putative |
|  | 1081 | | -0.43 | 13.15 | 2.89E-02 | At3g62790 | expressed protein |
|  | 1082 | | -0.51 | 9.58 | 2.89E-02 | At4g17670 | senescence-associated protein -related |
|  | 1083 | | 0.63 | 14.38 | 2.89E-02 | At1g19570 | dehydroascorbate reductase, putative |
|  | 1084 | | -0.41 | 10.97 | 2.89E-02 | At5g22280 | expressed protein |
|  | 1085 | | -0.38 | 9.09 | 2.89E-02 | At5g23320 | farnesyl cysteine carboxyl methyltransferase-related protein |
|  | 1086 | | 0.65 | 10.60 | 2.89E-02 | At3g63010 | expressed protein |
|  | 1087 | | -0.99 | 11.80 | 2.90E-02 | At1g77930 | DnaJ protein family |
|  | 1088 | | -0.38 | 13.36 | 2.90E-02 | At1g16880 | expressed protein |
|  | 1089 | | -0.65 | 9.07 | 2.90E-02 | At2g27420 | cysteine proteinase |
|  | 1090 | | 0.97 | 10.56 | 2.91E-02 |  | Sequence of BAC F14L17 from Arabidopsis thaliana chromosome 1 |
|  | 1091 | | 0.48 | 10.74 | 2.92E-02 | At3g58750 | citrate synthase -related protein |
|  | 1092 | | 0.37 | 10.11 | 2.92E-02 | At5g40240 | nodulin MtN21 family protein |
|  | 1093 | | -0.75 | 9.25 | 2.94E-02 | At1g19200 | senescence-associated protein -related |
|  | 1094 | | -0.49 | 12.05 | 2.95E-02 | At3g03100 | expressed protein |
|  | 1095 | | -0.50 | 11.15 | 2.95E-02 | At5g57810 | senescence-associated protein- related |
|  | 1096 | | -0.44 | 11.45 | 2.96E-02 | At2g18540 | cupin domain-containing protein |
|  | 1097 | | 0.43 | 8.91 | 2.96E-02 | At3g32040 | geranylgeranyl pyrophosphate synthase (GGPS1)(farnesyltranstransferase), putative |
|  | 1098 | | 0.60 | 10.17 | 2.96E-02 | At4g36360 | glycosyl hydrolase family 35 (beta-galactosidase) |
|  | 1099 | | 0.39 | 11.14 | 2.96E-02 | At5g17380 | 2-hydroxyphytanoyl-CoA lyase-related protein |
|  | 1100 | | 0.49 | 10.99 | 2.96E-02 | At4g18440 | Arabidopsis thaliana DNA chromosome 4, BAC clone F28J12 (ESSAII project) |
|  | 1101 | | 0.60 | 11.67 | 2.96E-02 | At3g49680 | branched-chain-amino-acid transaminase -related protein |
|  | 1102 | | 0.37 | 9.89 | 2.96E-02 | At2g44530 | phosphoribosyl pyrophosphate synthetase -related |
|  | 1103 | | -0.51 | 11.21 | 2.96E-02 | At5g38510 | hypothetical protein |
|  | 1104 | | -0.43 | 10.43 | 2.96E-02 | At5g65300 | expressed protein |
|  | 1105 | | 0.42 | 9.65 | 2.96E-02 | At1g26270 | phosphatidylinositol 3- and 4-kinase family |
|  | 1106 | | 0.43 | 12.15 | 2.96E-02 | At1g56400 | F-box protein family |
|  | 1107 | | 0.42 | 12.22 | 2.97E-02 | At3g59060 | bHLH protein family |
|  | 1108 | | 0.42 | 10.08 | 2.98E-02 | At3g62010 | expressed protein |
|  | 1109 | | -0.43 | 9.45 | 2.98E-02 | At5g42670 | expressed protein |
|  | 1110 | | -0.41 | 9.87 | 2.98E-02 | At5g57170 | Macrophage migration inhibitory factor (MIF) family |
|  | 1111 | | 0.48 | 10.22 | 2.98E-02 | At1g22360 | UDP-glycosyltransferase family |
|  | 1112 | | 0.59 | 9.67 | 2.98E-02 | At3g20580 | predicted GPI-anchored protein |
|  | 1113 | | 0.62 | 12.85 | 2.98E-02 | At1g79550 | phosphoglycerate kinase -related |
|  | 1114 | | -0.47 | 9.98 | 2.98E-02 | At2g18210 | expressed protein |
|  | 1115 | | 0.57 | 14.31 | 2.98E-02 | At4g35390 | expressed protein |
|  | 1116 | | 0.61 | 12.56 | 2.98E-02 | At1g32540 | zinc-finger protein -related |
|  | 1117 | | -0.52 | 10.43 | 2.99E-02 | At2g35850 | hypothetical protein |
|  | 1118 | | 0.85 | 13.73 | 2.99E-02 | At4g38770 | proline-rich protein family |
|  | 1119 | | 0.54 | 9.47 | 2.99E-02 | At1g04220 | beta-ketoacyl-CoA synthase, putative |
|  | 1120 | | -0.51 | 10.57 | 3.01E-02 |  | Arabidopsis thaliana genomic DNA, chromosome 3, BAC clone:F5N5 |
|  | 1121 | | -0.43 | 11.59 | 3.02E-02 | At2g45990 | expressed protein |
|  | 1122 | | 0.38 | 9.36 | 3.02E-02 | At5g20250 | glycosyl hydrolase family 36 |
|  | 1123 | | 0.58 | 9.98 | 3.02E-02 | At5g65950 | expressed protein |
|  | 1124 | | 0.52 | 14.14 | 3.02E-02 | At3g11940 | 40S ribosomal protein S5 (RPS5B) |
|  | 1125 | | -0.40 | 9.14 | 3.02E-02 | At1g67070 | phosphomannose isomerase -related |
|  | 1126 | | 0.49 | 9.57 | 3.02E-02 | At4g30790 | expressed protein |
|  | 1127 | | -0.49 | 9.93 | 3.02E-02 | At3g52740 | expressed protein |
|  | 1128 | | 0.45 | 11.70 | 3.02E-02 | At4g27500 | proton pump interactor |
|  | 1129 | | -0.38 | 10.60 | 3.02E-02 | At1g05720 | expressed protein |
|  | 1130 | | -0.37 | 12.81 | 3.03E-02 | At5g14970 | seed maturation -related protein |
|  | 1131 | | -0.55 | 11.10 | 3.03E-02 | At2g47640 | small nuclear ribonucleo protein D2 -related |
|  | 1132 | | 0.77 | 12.28 | 3.03E-02 |  | Arabidopsis thaliana chromosome I BAC F28N24 genomic sequence |
|  | 1133 | | -0.43 | 14.25 | 3.03E-02 | At1g66240 | copper homeostasis factor (copper chaperone) (CCH), putative |
|  | 1134 | | 0.54 | 14.31 | 3.04E-02 | At3g58570 | DEAD box RNA helicase, putative |
|  | 1135 | | 0.74 | 11.51 | 3.04E-02 | At4g22520 | protease inhibitor/seed storage/lipid transfer protein (LTP) family |
|  | 1136 | | -0.72 | 9.96 | 3.05E-02 | At4g34790 | auxin-induced (indole-3-acetic acid induced) protein family |
|  | 1137 | | -0.78 | 11.59 | 3.06E-02 |  | Arabidopsis thaliana AT3g47961 gene, complete cds |
|  | 1138 | | 0.40 | 9.58 | 3.06E-02 | At5g11110 | sucrose-phosphate synthase, putative |
|  | 1139 | | 0.62 | 9.67 | 3.06E-02 | At3g62720 | transferase - related |
|  | 1140 | | 0.58 | 10.28 | 3.06E-02 | At4g00890 | proline-rich protein family |
|  | 1141 | | 0.50 | 10.20 | 3.06E-02 | At3g48860 | expressed protein |
|  | 1142 | | -0.44 | 11.15 | 3.08E-02 | At2g22860 | (AtPSK) phytosulfokine precursor 2 |
|  | 1143 | | -0.43 | 10.55 | 3.08E-02 | At2g38740 | haloacid dehalogenase-like hydrolase family |
|  | 1144 | | 0.46 | 9.27 | 3.08E-02 | At5g06490 | zinc finger (C3HC4-type RING finger) protein family |
|  | 1145 | | -0.71 | 12.08 | 3.09E-02 | At2g04700 | ferredoxin-thioredoxin reductase -related |
|  | 1146 | | 0.75 | 10.82 | 3.10E-02 | At3g14067 | subtilisin-like serine protease, putative |
|  | 1147 | | 0.94 | 12.76 | 3.10E-02 | At1g71880 | sucrose transporter SUC1 (sucrose-proton symporter) |
|  | 1148 | | -0.45 | 12.53 | 3.10E-02 | At1g56580 | expressed protein |
|  | 1149 | | -0.49 | 12.76 | 3.10E-02 | At3g25260 | nitrate transporter -related |
|  | 1150 | | 0.48 | 9.58 | 3.11E-02 | At3g03380 | DegP protease, putative |
|  | 1151 | | 0.91 | 13.17 | 3.11E-02 | At3g23295 | Arabidopsis thaliana genomic DNA, chromosome 3, P1 clone: MLM24 |
|  | 1152 | | 0.33 | 12.17 | 3.11E-02 | At4g17870 | expressed protein |
|  | 1153 | | 0.58 | 10.33 | 3.11E-02 | At5g36880 | acetyl-CoA synthetase (acetate-CoA ligase), putative |
|  | 1154 | | 0.44 | 11.02 | 3.11E-02 | At5g49690 | UDP-glycosyltransferase family |
|  | 1155 | | 0.77 | 10.28 | 3.11E-02 | At1g22770 | gigantea protein -related |
|  | 1156 | | -0.45 | 14.35 | 3.11E-02 | At3g06700 | Arabidopsis thaliana ribosomal protein L29, putative; 3222-3503 (T8E24.8) mRNA, complete cds |
|  | 1157 | | -0.76 | 9.91 | 3.11E-02 | At4g15670 | glutaredoxin protein family |
|  | 1158 | | -0.44 | 9.92 | 3.11E-02 | At2g31710 | expressed protein |
|  | 1159 | | 0.83 | 10.10 | 3.12E-02 | At1g05530 | glycosyltransferase family |
|  | 1160 | | 0.84 | 10.32 | 3.12E-02 | At3g52500 | expressed protein |
|  | 1161 | | -0.59 | 9.45 | 3.12E-02 | At1g26400 | FAD-linked oxidoreductase family |
|  | 1162 | | 0.76 | 14.02 | 3.12E-02 | At1g18830 | transducin / WD-40 repeat protein family |
|  | 1163 | | -0.44 | 11.51 | 3.13E-02 | At1g45474 | light-harvesting chlorophyll a/b binding protein |
|  | 1164 | | 0.52 | 10.21 | 3.13E-02 | At5g62680 | peptide transporter |
|  | 1165 | | 0.38 | 10.47 | 3.13E-02 | At1g08315 | armadillo repeat containing protein |
|  | 1166 | | -0.53 | 10.72 | 3.13E-02 | At3g24010 | PHD-finger protein -related |
|  | 1167 | | 0.56 | 11.91 | 3.13E-02 | At3g51780 | expressed protein |
|  | 1168 | | -0.50 | 10.56 | 3.14E-02 | At1g01380 | myb family transcription factor |
|  | 1169 | | 0.55 | 11.12 | 3.14E-02 | At1g02170 | expressed protein |
|  | 1170 | | -0.68 | 12.10 | 3.14E-02 | At3g50480 | expressed protein |
|  | 1171 | | 0.53 | 11.80 | 3.14E-02 | At1g03860 | prohibitin 2 -related |
|  | 1172 | | 0.34 | 10.33 | 3.15E-02 | At5g40340 | PWWP domain protein |
|  | 1173 | | 0.62 | 11.64 | 3.16E-02 | At5g49910 | heat shock protein cpHsc70-2 (hsc70-7) |
|  | 1174 | | -0.45 | 12.40 | 3.16E-02 | At1g42960 | expressed protein |
|  | 1175 | | -0.93 | 10.13 | 3.16E-02 | At2g20670 | expressed protein |
|  | 1176 | | -0.48 | 10.29 | 3.16E-02 | At3g25070 | expressed protein |
|  | 1177 | | 0.49 | 10.93 | 3.16E-02 | At3g11450 | cell division protein - related |
|  | 1178 | | 0.57 | 9.60 | 3.16E-02 | At1g24350 | Genomic sequence for Arabidopsis thaliana BAC F21J9 from chromosome I |
|  | 1179 | | 0.49 | 9.29 | 3.17E-02 | At1g71410 | protein kinase family |
|  | 1180 | | 0.55 | 12.91 | 3.17E-02 | At3g15780 | expressed protein |
|  | 1181 | | -0.53 | 11.93 | 3.17E-02 | At1g16240 | syntaxin of plants 51 (SYP51) |
|  | 1182 | | -0.50 | 12.24 | 3.18E-02 |  | Genomic sequence for Arabidopsis thaliana BAC F2E2 from chromosome I |
|  | 1183 | | 0.57 | 10.37 | 3.19E-02 | At2g40070 | En/Spm-related transposon protein |
|  | 1184 | | 0.47 | 9.88 | 3.19E-02 | At5g65750 | 2-oxoglutarate dehydrogenase, E1 component |
|  | 1185 | | 0.43 | 11.78 | 3.20E-02 | At5g58140 | non phototropic hypocotyl 1-related |
|  | 1186 | | -0.35 | 12.24 | 3.20E-02 | At2g43910 | thiol methyltransferase |
|  | 1187 | | 0.39 | 10.83 | 3.20E-02 | At1g42550 | expressed protein |
|  | 1188 | | -0.58 | 10.10 | 3.20E-02 | At1g17455 | Genomic sequence for Arabidopsis thaliana BAC F1L3 from chromosome I |
|  | 1189 | | 0.54 | 10.09 | 3.20E-02 | At5g40770 | prohibitin (gb|AAC49691.1) |
|  | 1190 | | -0.46 | 9.09 | 3.20E-02 | At1g09720 | hypothetical protein |
|  | 1191 | | 0.49 | 12.23 | 3.21E-02 | At5g21960 | AP2 domain transcription factor, putative |
|  | 1192 | | -0.76 | 10.19 | 3.21E-02 | At2g41250 | haloacid dehalogenase-like hydrolase family |
|  | 1193 | | -0.42 | 13.94 | 3.22E-02 | At5g44430 | plant defensin protein, putative (PDF1.2c) |
|  | 1194 | | -0.44 | 9.61 | 3.22E-02 |  | Sequence of BAC F7G19 from Arabidopsis thaliana chromosome 1 |
|  | 1195 | | 1.26 | 10.54 | 3.22E-02 | At1g07400 | heat shock protein, putative |
|  | 1196 | | 0.76 | 10.41 | 3.23E-02 | At3g07100 | protein transport protein Sec24, putative |
|  | 1197 | | 0.54 | 9.72 | 3.23E-02 | At4g30100 | expressed protein |
|  | 1198 | | -0.48 | 10.93 | 3.23E-02 | At3g02870 | myo-inositol monophosphatase -related |
|  | 1199 | | 0.40 | 11.44 | 3.23E-02 | At1g25141 | F-box protein-related |
|  | 1200 | | 0.41 | 10.17 | 3.24E-02 | At3g18560 | expressed protein |
|  | 1201 | | 0.44 | 11.86 | 3.25E-02 | At2g33860 | auxin response transcription factor 3 (ETTIN/ARF3) |
|  | 1202 | | -0.51 | 9.66 | 3.25E-02 | At1g50980 | F-box protein family |
|  | 1203 | | -0.43 | 8.60 | 3.25E-02 | At2g21480 | protein kinase family |
|  | 1204 | | -0.36 | 10.21 | 3.25E-02 | At2g24290 | expressed protein |
|  | 1205 | | 0.39 | 11.81 | 3.25E-02 | At3g30390 | amino acid transporter family |
|  | 1206 | | -0.51 | 9.83 | 3.25E-02 | At5g14710 | expressed protein |
|  | 1207 | | 0.48 | 11.80 | 3.25E-02 | At5g03970 | F-box protein family |
|  | 1208 | | -0.45 | 9.70 | 3.25E-02 | At5g48660 | hypothetical protein |
|  | 1209 | | 0.46 | 10.36 | 3.25E-02 | At3g10070 | conserved hypothetical protein |
|  | 1210 | | 0.49 | 12.52 | 3.25E-02 | At3g17390 | s-adenosylmethionine synthetase -related |
|  | 1211 | | -0.73 | 9.51 | 3.25E-02 | At2g17660 | expressed protein |
|  | 1212 | | 0.70 | 9.73 | 3.25E-02 | At3g13990 | expressed protein |
|  | 1213 | | -0.52 | 9.24 | 3.25E-02 | At5g38900 | frnE protein - like |
|  | 1214 | | 0.79 | 10.18 | 3.25E-02 | At1g10950 | endomembrane protein 70, putative |
|  | 1215 | | -0.68 | 10.29 | 3.26E-02 | At1g35230 | arabinogalactan-protein (AGP5) |
|  | 1216 | | 0.47 | 12.48 | 3.27E-02 | At4g23100 | gamma-glutamylcysteine synthetase |
|  | 1217 | | -0.52 | 9.43 | 3.28E-02 | At4g01900 | P II nitrogen sensing protein GLB I |
|  | 1218 | | 0.56 | 10.48 | 3.29E-02 | At3g24240 | leucine-rich repeat transmembrane protein kinase, putative |
|  | 1219 | | -1.01 | 11.39 | 3.30E-02 | At5g18080 | auxin-induced (indole-3-acetic acid induced) protein, putative |
|  | 1220 | | 0.47 | 10.44 | 3.30E-02 | At5g58620 | zinc finger transcription factor-related protein |
|  | 1221 | | 0.69 | 13.86 | 3.30E-02 | At5g35935 | ESTs, Weakly similar to T45948 hypothetical protein F7J8.60 - Arabidopsis thaliana [A.thaliana] |
|  | 1222 | | -0.61 | 11.43 | 3.31E-02 | At4g12720 | MutT/nudix family protein |
|  | 1223 | | -0.51 | 10.76 | 3.31E-02 | At4g32590 | ferredoxin - related |
|  | 1224 | | -0.47 | 12.32 | 3.31E-02 | At1g52220 | expressed protein |
|  | 1225 | | 0.39 | 10.31 | 3.32E-02 | At4g02630 | protein kinase family |
|  | 1226 | | 0.37 | 9.30 | 3.32E-02 | At1g32240 | myb family transcription factor |
|  | 1227 | | 0.62 | 9.13 | 3.32E-02 | At4g16340 | expressed protein |
|  | 1228 | | -0.82 | 12.38 | 3.32E-02 | At2g28720 | histone H2B, putative |
|  | 1229 | | 0.52 | 10.51 | 3.32E-02 | At4g38550 | Phospholipase like protein |
|  | 1230 | | 0.57 | 12.17 | 3.32E-02 | At3g48690 | expressed protein |
|  | 1231 | | -0.55 | 9.39 | 3.32E-02 | At1g49200 | Genomic sequence for Arabidopsis thaliana BAC F27J15 from chromosome I |
|  | 1232 | | 0.42 | 12.14 | 3.32E-02 | At1g04410 | malate dehydrogenase, cytosolic, putative |
|  | 1233 | | -0.66 | 11.09 | 3.33E-02 | At5g55460 | protease inhibitor/seed storage/lipid transfer protein (LTP) family |
|  | 1234 | | -1.15 | 11.24 | 3.33E-02 | At3g56290 | expressed protein |
|  | 1235 | | -0.44 | 8.86 | 3.33E-02 | At1g11540 | expressed protein |
|  | 1236 | | 0.43 | 8.77 | 3.33E-02 | At5g23050 | AMP-dependent synthetase and ligase family |
|  | 1237 | | 0.39 | 14.60 | 3.33E-02 | At1g17640 | RNA recognition motif (RRM)-containing protein |
|  | 1238 | | 0.35 | 10.27 | 3.33E-02 | At1g27630 | cyclin family |
|  | 1239 | | 0.78 | 9.66 | 3.33E-02 | At1g70300 | potassium transporter, putative (KUP6/HAK6/KT6/POT6) |
|  | 1240 | | -0.40 | 10.24 | 3.33E-02 | At1g48140 | expressed protein |
|  | 1241 | | 0.79 | 11.36 | 3.33E-02 |  | Arabidopsis thaliana chromosome 1 YAC YUP8H12R sequence, complete sequence |
|  | 1242 | | -0.39 | 14.24 | 3.33E-02 | At1g03130 | photosystem I reaction center subunit II precursor -related |
|  | 1243 | | -0.55 | 12.93 | 3.33E-02 | At3g56910 | ribosomal protein, chloroplast |
|  | 1244 | | 0.48 | 9.42 | 3.34E-02 | At1g15440 | transducin / WD-40 repeat protein family |
|  | 1245 | | 0.37 | 10.56 | 3.34E-02 | At1g30970 | zinc finger protein -related |
|  | 1246 | | -0.64 | 11.82 | 3.34E-02 | At5g09920 | 15.9 kDa subunit of RNA polymerase II (gb|AAB95261.1) |
|  | 1247 | | 0.36 | 12.73 | 3.36E-02 | At2g19310 | small heat shock protein -related |
|  | 1248 | | 0.55 | 9.63 | 3.36E-02 | At3g26240 | CHP-rich zinc finger protein, putative |
|  | 1249 | | 0.39 | 9.81 | 3.37E-02 | At5g61140 | DEAD box RNA helicase, putative |
|  | 1250 | | 0.47 | 9.69 | 3.38E-02 | At3g29595 | Arabidopsis thaliana genomic DNA, chromosome 3, P1 clone: MTO24 |
|  | 1251 | | -0.32 | 12.44 | 3.38E-02 | At4g27700 | rhodanese-like domain protein |
|  | 1252 | | -0.41 | 12.34 | 3.38E-02 | At2g46820 | expressed protein |
|  | 1253 | | -0.49 | 9.48 | 3.38E-02 | At5g62100 | BAG domain containing protein |
|  | 1254 | | -0.50 | 10.13 | 3.38E-02 | At1g27610 | hypothetical protein |
|  | 1255 | | -0.64 | 12.16 | 3.38E-02 | At1g16810 | expressed protein |
|  | 1256 | | -0.39 | 9.25 | 3.38E-02 | At1g62840 | hypothetical protein |
|  | 1257 | | 0.36 | 10.29 | 3.38E-02 | At5g59950 | RNA and export factor binding protein, putative |
|  | 1258 | | 0.38 | 9.85 | 3.38E-02 | At1g19680 | expressed protein |
|  | 1259 | | 0.32 | 9.25 | 3.39E-02 | At4g31920 | two-component response regulator protein family |
|  | 1260 | | 0.56 | 10.17 | 3.39E-02 | At3g18490 | chloroplast nucleoid DNA-binding protein -related |
|  | 1261 | | -0.50 | 12.96 | 3.39E-02 | At1g24735 | caffeoyl-CoA 3-O-methyltransferase, putative |
|  | 1262 | | 0.43 | 10.45 | 3.39E-02 | At1g16190 | EST |
|  | 1263 | | 0.56 | 11.31 | 3.39E-02 | At5g47480 | expressed protein |
|  | 1264 | | -0.50 | 9.28 | 3.39E-02 | At5g62480 | glutathione transferase, putative |
|  | 1265 | | 0.50 | 10.94 | 3.40E-02 | At1g28380 | expressed protein |
|  | 1266 | | 0.38 | 9.42 | 3.40E-02 | At4g23840 | Arabidopsis thaliana DNA chromosome 4, BAC clone T32A16 (ESSA project) |
|  | 1267 | | -0.36 | 9.00 | 3.41E-02 | At3g24150 | expressed protein |
|  | 1268 | | -0.55 | 10.01 | 3.41E-02 | At1g56260 | hypothetical protein |
|  | 1269 | | -0.71 | 9.96 | 3.42E-02 | At3g51750 | expressed protein |
|  | 1270 | | -1.15 | 9.74 | 3.42E-02 | At2g26400 | hypothetical protein |
|  | 1271 | | -0.39 | 10.59 | 3.42E-02 | At2g38780 | expressed protein |
|  | 1272 | | 0.38 | 10.44 | 3.42E-02 | At3g50270 | hydroxycinnamoyl/benzoyltransferase-related |
|  | 1273 | | 0.60 | 11.04 | 3.44E-02 | At1g80780 | CCR4-associated factor -related\0CCR4-associated factor, putative |
|  | 1274 | | -0.32 | 10.24 | 3.44E-02 | At4g19270 | hypothetical protein |
|  | 1275 | | 0.94 | 12.39 | 3.44E-02 | At5g48180 | Kelch repeats protein family |
|  | 1276 | | 0.59 | 10.08 | 3.45E-02 | At1g07680 | hypothetical protein |
|  | 1277 | | 0.38 | 14.67 | 3.45E-02 | At5g52650 | 40S ribosomal protein S10 (RPS10C) |
|  | 1278 | | 0.56 | 10.92 | 3.45E-02 | At1g70600 | EST |
|  | 1279 | | 0.43 | 10.45 | 3.45E-02 | At1g79900 | mitochondrial carrier protein family |
|  | 1280 | | 0.62 | 9.86 | 3.46E-02 | At5g56040 | leucine rich repeat protein kinase, putative |
|  | 1281 | | 0.46 | 9.10 | 3.46E-02 | At1g79130 | auxin-induced (indole-3-acetic acid induced) protein family |
|  | 1282 | | -0.39 | 9.12 | 3.46E-02 | At3g47020 | F-box protein family |
|  | 1283 | | -0.42 | 11.10 | 3.46E-02 | At1g09415 | expressed protein |
|  | 1284 | | -0.47 | 10.49 | 3.46E-02 | At3g19800 | expressed protein |
|  | 1285 | | 0.42 | 9.29 | 3.48E-02 | At3g11890 | expressed protein |
|  | 1286 | | 0.42 | 9.63 | 3.49E-02 | At3g16060 | kinesin-like protein TBK5, putative |
|  | 1287 | | -0.41 | 9.22 | 3.49E-02 | At3g62220 | serine/threonine protein kinase, putative |
|  | 1288 | | -0.52 | 9.06 | 3.49E-02 | At4g30180 | expressed protein |
|  | 1289 | | 0.53 | 13.28 | 3.49E-02 | At4g23470 | proline-rich protein family |
|  | 1290 | | 0.50 | 9.90 | 3.49E-02 | At1g20960 | U5 small nuclear ribonucleoprotein helicase, putative |
|  | 1291 | | 0.40 | 10.56 | 3.49E-02 | At5g04410 | Arabidopsis thaliana NAC2 (NAC2) mRNA, complete cds |
|  | 1292 | | 0.88 | 11.92 | 3.50E-02 | At1g35617 | Genomic sequence for Arabidopsis thaliana BAC F15O4 from chromosome I |
|  | 1293 | | 0.62 | 9.85 | 3.50E-02 | At5g65650 | expressed protein |
|  | 1294 | | 0.40 | 11.32 | 3.50E-02 | At3g63150 | GTP-binding protein - related |
|  | 1295 | | -0.42 | 10.65 | 3.51E-02 | At1g10350 | heat shock protein, putative |
|  | 1296 | | -0.43 | 11.96 | 3.51E-02 | At2g33220 | expressed protein |
|  | 1297 | | 0.80 | 11.44 | 3.51E-02 | At3g08530 | clathrin heavy chain -related |
|  | 1298 | | 0.54 | 9.75 | 3.52E-02 | At5g57560 | xyloglucan endotransglycosylase (TCH4) |
|  | 1299 | | 0.78 | 9.59 | 3.53E-02 | At2g22125 | C2 domain-containing protein |
|  | 1300 | | -0.45 | 10.64 | 3.55E-02 | At3g03160 | expressed protein |
|  | 1301 | | 0.58 | 11.78 | 3.55E-02 | At5g05290 | expansin, putative (EXP2) |
|  | 1302 | | 0.33 | 13.23 | 3.56E-02 | At1g43170 | 60S ribosomal protein L3 (RPL3A) |
|  | 1303 | | -0.47 | 9.36 | 3.56E-02 | At2g40260 | myb family transcription factor |
|  | 1304 | | -0.41 | 12.85 | 3.57E-02 | At2g21530 | expressed protein |
|  | 1305 | | -0.58 | 10.59 | 3.57E-02 | At2g43760 | molybdopterin synthase large subunit -related |
|  | 1306 | | 0.43 | 10.08 | 3.57E-02 | At1g56170 | transcription factor Hap5a, putative |
|  | 1307 | | -0.49 | 10.33 | 3.58E-02 | At2g23590 | hydrolase, alpha/beta fold family |
|  | 1308 | | -0.49 | 12.89 | 3.58E-02 | At2g02510 | expressed protein |
|  | 1309 | | 0.58 | 11.47 | 3.59E-02 | At5g49910 | heat shock protein cpHsc70-2 (hsc70-7) |
|  | 1310 | | 0.58 | 10.76 | 3.59E-02 | At1g08000 | GATA zinc finger protein |
|  | 1311 | | 0.47 | 10.58 | 3.59E-02 | At3g57150 | pseudouridine synthase (NAP57) -related |
|  | 1312 | | 0.41 | 9.62 | 3.59E-02 | At3g24740 | expressed protein |
|  | 1313 | | 0.61 | 11.00 | 3.60E-02 | At1g25280 | F-box containing tubby family protein |
|  | 1314 | | -0.80 | 10.62 | 3.60E-02 | At1g29510 | auxin-induced (indole-3-acetic acid induced) protein, putative |
|  | 1315 | | 0.54 | 14.03 | 3.61E-02 | At3g24860 | proline-rich protein family |
|  | 1316 | | 0.56 | 11.56 | 3.61E-02 | At1g18080 | WD-40 repeat auxin-dependent protein ARCA |
|  | 1317 | | 0.46 | 9.41 | 3.61E-02 | At4g32620 | expressed protein |
|  | 1318 | | -0.52 | 11.53 | 3.61E-02 | At4g04840 | expressed protein |
|  | 1319 | | 0.71 | 11.13 | 3.61E-02 | At5g05780 | 26S proteasome regulatory subunit S12 (RPN8), putative |
|  | 1320 | | 0.47 | 10.16 | 3.61E-02 | At4g14900 | proline-rich protein family |
|  | 1321 | | -0.41 | 9.69 | 3.61E-02 | At3g09160 | RNA recognition motif (RRM)-containing protein |
|  | 1322 | | -0.47 | 10.26 | 3.61E-02 | At1g47610 | transducin / WD-40 repeat protein family |
|  | 1323 | | 0.59 | 9.58 | 3.61E-02 | At4g14160 | transport protein |
|  | 1324 | | -0.66 | 10.66 | 3.61E-02 | At5g39210 | expressed protein |
|  | 1325 | | 0.53 | 12.44 | 3.61E-02 | At2g15550 | hypothetical protein |
|  | 1326 | | -0.43 | 9.68 | 3.61E-02 | At3g46280 | expressed protein |
|  | 1327 | | 0.37 | 10.29 | 3.62E-02 | At4g19090 | hypothetical protein |
|  | 1328 | | -0.44 | 10.74 | 3.62E-02 | At1g29040 | expressed protein |
|  | 1329 | | -0.33 | 9.55 | 3.63E-02 | At2g32730 | EST, Weakly similar to T00795 hypothetical protein F24L7.13 - Arabidopsis thaliana [A.thaliana] |
|  | 1330 | | -0.54 | 13.54 | 3.64E-02 | At2g35810 | expressed protein |
|  | 1331 | | 0.63 | 11.62 | 3.64E-02 | At5g49910 | heat shock protein cpHsc70-2 (hsc70-7) |
|  | 1332 | | -0.39 | 11.84 | 3.64E-02 | At4g26400 | expressed protein |
|  | 1333 | | 0.51 | 10.76 | 3.64E-02 | At5g57790 | expressed protein |
|  | 1334 | | 0.43 | 9.45 | 3.64E-02 | At5g57260 | cytochrome P450 71B10 |
|  | 1335 | | 0.74 | 11.76 | 3.64E-02 | At3g06490 | myb family transcription factor (MYB108) |
|  | 1336 | | -0.58 | 8.97 | 3.64E-02 | At5g17830 | hypothetical protein |
|  | 1337 | | 0.33 | 9.24 | 3.64E-02 | At1g50660 | expressed protein |
|  | 1338 | | -0.42 | 11.41 | 3.64E-02 | At5g58787 | zinc finger (C3HC4-type RING finger) protein family |
|  | 1339 | | -0.56 | 10.95 | 3.65E-02 | At5g13830 | FtsJ (dbj|BAA83750.1) |
|  | 1340 | | -0.61 | 11.55 | 3.65E-02 | At4g30330 | small nuclear ribonucleo protein homolog |
|  | 1341 | | -0.61 | 11.36 | 3.65E-02 | At3g45860 | EST, Weakly similar to T47526 protein kinase-like - Arabidopsis thaliana [A.thaliana] |
|  | 1342 | | -0.46 | 9.69 | 3.65E-02 | At3g10210 | expressed protein |
|  | 1343 | | -0.55 | 13.53 | 3.65E-02 | At5g02570 | histone H2B, putative |
|  | 1344 | | -0.65 | 10.15 | 3.66E-02 | At4g12090 | hypothetical protein |
|  | 1345 | | 0.40 | 8.99 | 3.66E-02 | At3g59460 | hypothetical protein |
|  | 1346 | | 0.42 | 10.08 | 3.67E-02 | At2g44500 | axi 1 protein from Nicotiana tabacum -related |
|  | 1347 | | 0.53 | 12.19 | 3.67E-02 | At4g19420 | pectinacetylesterase family |
|  | 1348 | | 0.55 | 10.65 | 3.70E-02 | At2g19270 | expressed protein |
|  | 1349 | | -0.49 | 10.90 | 3.70E-02 | At2g24765 | ADP-ribosylation factor 3 (ARF3) |
|  | 1350 | | -0.54 | 10.67 | 3.70E-02 | At3g55630 | folylpolyglutamate-dihydrofolate synthase (dhfs/fpgs4) |
|  | 1351 | | -0.44 | 13.87 | 3.70E-02 | At1g69410 | Eukaryotic initiation factor 5A -related |
|  | 1352 | | 0.59 | 11.73 | 3.70E-02 | At5g49910 | heat shock protein cpHsc70-2 (hsc70-7) |
|  | 1353 | | 0.47 | 11.28 | 3.72E-02 | At2g47520 | AP2 domain transcription factor, putative |
|  | 1354 | | 0.59 | 11.86 | 3.72E-02 | At5g49910 | heat shock protein cpHsc70-2 (hsc70-7) |
|  | 1355 | | 0.47 | 9.85 | 3.73E-02 | At1g02660 | lipase (class 3) family |
|  | 1356 | | 0.54 | 9.91 | 3.73E-02 | At2g46660 | cytochrome P450, putative |
|  | 1357 | | 0.68 | 11.51 | 3.74E-02 | At5g61960 | Meiosis protein mei2-related |
|  | 1358 | | 0.45 | 13.21 | 3.74E-02 | At1g04610 | flavin-containing monooxygenase (FMO/YUCCA3) |
|  | 1359 | | 0.40 | 9.92 | 3.74E-02 | At4g08350 | expressed protein |
|  | 1360 | | -0.34 | 9.94 | 3.74E-02 | At5g52710 | heavy-metal-associated domain-containing protein |
|  | 1361 | | 0.46 | 9.67 | 3.74E-02 | At3g26560 | ATP-dependent RNA helicase, putative |
|  | 1362 | | -0.48 | 11.18 | 3.74E-02 | At5g35170 | adenylate kinase -related protein |
|  | 1363 | | 0.42 | 9.47 | 3.74E-02 | At4g34830 | pentatricopeptide (PPR) repeat-containing protein |
|  | 1364 | | 0.38 | 9.73 | 3.74E-02 | At2g25760 | protein kinase family |
|  | 1365 | | 0.46 | 10.04 | 3.74E-02 | At4g12650 | endomembrane protein 70, putative |
|  | 1366 | | -0.49 | 13.13 | 3.74E-02 | At3g04730 | auxin-responsive protein IAA16 (Indoleacetic acid-induced protein 16) |
|  | 1367 | | -0.52 | 9.83 | 3.75E-02 | At5g41080 | expressed protein |
|  | 1368 | | 0.81 | 9.25 | 3.75E-02 |  | EST |
|  | 1369 | | -0.53 | 9.25 | 3.75E-02 | At2g19190 | light repressible receptor protein kinase, putative |
|  | 1370 | | -0.49 | 10.39 | 3.75E-02 | At5g22380 | No apical meristem (NAM) protein family |
|  | 1371 | | 0.67 | 10.90 | 3.75E-02 | At3g49750 | leucine rich repeat protein family |
|  | 1372 | | 0.48 | 9.82 | 3.75E-02 | At4g36360 | Arabidopsis thaliana DNA chromosome 4, BAC clone F23E13 (ESSAII project) |
|  | 1373 | | -0.43 | 9.53 | 3.75E-02 | At4g36110 | auxin-induced (indole-3-acetic acid induced) protein, putative |
|  | 1374 | | 0.59 | 9.35 | 3.75E-02 | At4g01870 | expressed protein |
|  | 1375 | | 0.58 | 9.80 | 3.76E-02 | At3g25290 | auxin-induced protein family |
|  | 1376 | | -0.47 | 9.26 | 3.78E-02 | At5g49040 | disease resistance response protein-related/ dirigent protein-related |
|  | 1377 | | -0.49 | 9.45 | 3.78E-02 | At3g15270 | squamosa promoter binding protein-related 5 |
|  | 1378 | | -0.61 | 12.74 | 3.78E-02 | At3g09860 | hypothetical protein |
|  | 1379 | | -0.44 | 13.51 | 3.78E-02 | At5g42300 | ubiquitin family |
|  | 1380 | | 0.47 | 13.28 | 3.78E-02 | At4g17230 | scarecrow-like transcription factor 13 (SCL13) |
|  | 1381 | | -0.37 | 12.54 | 3.78E-02 | At5g17470 | calmodulin-related protein, putative |
|  | 1382 | | -0.39 | 8.76 | 3.80E-02 | At5g17750 | AAA-type ATPase family |
|  | 1383 | | 0.41 | 9.32 | 3.80E-02 | At5g04240 | zinc finger protein - like |
|  | 1384 | | 0.54 | 14.58 | 3.80E-02 | At5g50120 | transducin / WD-40 repeat protein family |
|  | 1385 | | 0.38 | 11.52 | 3.80E-02 | At4g22920 | expressed protein |
|  | 1386 | | -0.46 | 12.68 | 3.80E-02 | At2g22670 | auxin-responsive protein IAA8 (Indoleacetic acid-induced protein 8) |
|  | 1387 | | 0.42 | 10.87 | 3.80E-02 | At4g31450 | expressed protein |
|  | 1388 | | 0.45 | 10.01 | 3.80E-02 | At3g27740 | carbamoyl-phosphate synthase [glutamine-hydrolyzing] |
|  | 1389 | | 0.41 | 9.34 | 3.81E-02 | At3g14120 | expressed protein |
|  | 1390 | | -0.72 | 11.69 | 3.81E-02 | At5g16660 | protein; similar to unknown protein (gb|AAF26969.1) -related |
|  | 1391 | | -0.49 | 10.29 | 3.81E-02 | At4g30250 | AAA-type ATPase family |
|  | 1392 | | -0.36 | 13.69 | 3.81E-02 | At1g72020 | expressed protein |
|  | 1393 | | -0.52 | 13.68 | 3.81E-02 | At3g48930 | 40S ribosomal protein S11 (RPS11A) |
|  | 1394 | | 0.60 | 9.93 | 3.82E-02 | At3g11830 | chaperonin, putative |
|  | 1395 | | 0.37 | 9.71 | 3.82E-02 | At1g65580 | hypothetical protein |
|  | 1396 | | 0.52 | 10.80 | 3.82E-02 | At4g15710 | hypothetical protein |
|  | 1397 | | 0.45 | 10.49 | 3.82E-02 | At4g33510 | 2-dehydro-3-deoxyphosphoheptonate aldolase (DHS2) |
|  | 1398 | | 0.47 | 9.06 | 3.82E-02 | At1g61500 | S-locus protein kinase, putative |
|  | 1399 | | -0.35 | 9.95 | 3.82E-02 | At3g17365 | expressed protein |
|  | 1400 | | 0.59 | 10.14 | 3.82E-02 | At3g14660 | EST |
|  | 1401 | | -0.36 | 9.28 | 3.82E-02 | At3g51080 | GATA zinc finger protein |
|  | 1402 | | 0.35 | 10.11 | 3.82E-02 | At4g29120 | expressed protein |
|  | 1403 | | 0.37 | 10.85 | 3.83E-02 | At1g52360 | coatomer protein complex, subunit beta 2 (beta prime), putative |
|  | 1404 | | 0.55 | 10.16 | 3.83E-02 | At5g04970 | pectinesterase, putative |
|  | 1405 | | -0.51 | 10.06 | 3.83E-02 | At5g37480 | expressed protein |
|  | 1406 | | -0.77 | 14.34 | 3.83E-02 | At3g24370 | hypothetical protein |
|  | 1407 | | -0.55 | 11.57 | 3.83E-02 | At4g28025 | expressed protein |
|  | 1408 | | -0.37 | 10.71 | 3.84E-02 | At3g14880 | hypothetical protein |
|  | 1409 | | 0.32 | 11.08 | 3.84E-02 | At1g17720 | type 2A protein serine/threonine phosphatase 55 kDa B regulatory subunit |
|  | 1410 | | 0.60 | 14.72 | 3.84E-02 | At3g08580 | mitochondrial ADP,ATP carrier protein 1 |
|  | 1411 | | 0.59 | 9.82 | 3.84E-02 | At1g04445 | zinc finger (C2H2 type) protein family |
|  | 1412 | | 0.56 | 9.97 | 3.84E-02 | At1g14670 | endomembrane protein 70, putative |
|  | 1413 | | 0.69 | 10.33 | 3.84E-02 | At3g10650 | expressed protein |
|  | 1414 | | 0.44 | 10.98 | 3.85E-02 | At4g28910 | expressed protein |
|  | 1415 | | 0.49 | 10.51 | 3.85E-02 | At2g32490 | hypothetical protein |
|  | 1416 | | 0.60 | 11.01 | 3.86E-02 | At2g31390 | fructokinase, putative |
|  | 1417 | | -0.35 | 9.03 | 3.86E-02 | At1g08500 | plastocyanin-like domain containing protein |
|  | 1418 | | -0.82 | 12.64 | 3.87E-02 | At2g25080 | glutathione peroxidase, putative |
|  | 1419 | | 0.45 | 11.35 | 3.87E-02 | At1g64740 | tubulin alpha-1 chain (TUA1) |
|  | 1420 | | 0.57 | 9.06 | 3.87E-02 | At2g34680 | leucine rich repeat protein family |
|  | 1421 | | -0.68 | 10.86 | 3.87E-02 | At1g07010 | calcineurin-like phosphoesterase family |
|  | 1422 | | 0.89 | 11.84 | 3.88E-02 | At1g79750 | malate oxidoreductase -related |
|  | 1423 | | -0.43 | 9.47 | 3.88E-02 | At2g29230 | Mutator-related transposase |
|  | 1424 | | 0.49 | 10.07 | 3.89E-02 | At2g03260 | expressed protein |
|  | 1425 | | 0.46 | 11.40 | 3.89E-02 | At4g33070 | pyruvate decarboxylase-1 (Pdc1) |
|  | 1426 | | 0.46 | 10.26 | 3.89E-02 | At1g50200 | alanine--tRNA ligase -related |
|  | 1427 | | 0.41 | 11.82 | 3.89E-02 | At1g11840 | glyoxalase I, putative (lactoylglutathione lyase) |
|  | 1428 | | -0.37 | 9.79 | 3.89E-02 | At1g16060 | ovule development protein, putative |
|  | 1429 | | -0.41 | 9.02 | 3.90E-02 | At4g11070 | WRKY family transcription factor |
|  | 1430 | | 0.39 | 9.98 | 3.91E-02 | At5g60170 | RNA recognition motif (RRM)-containing protein |
|  | 1431 | | 0.44 | 9.68 | 3.91E-02 | At5g47690 | hypothetical protein |
|  | 1432 | | -0.54 | 12.28 | 3.91E-02 | At1g25400 | expressed protein |
|  | 1433 | | -0.62 | 9.72 | 3.91E-02 | At3g05830 | expressed protein |
|  | 1434 | | 0.52 | 10.76 | 3.92E-02 | At3g14350 | leucine-rich repeat transmembrane protein kinase, putative |
|  | 1435 | | -0.44 | 11.64 | 3.92E-02 | At2g44650 | chloroplast chaperonin 10 |
|  | 1436 | | -0.85 | 12.64 | 3.94E-02 | At1g68660 | expressed protein |
|  | 1437 | | 0.47 | 9.36 | 3.94E-02 | At3g21540 | transducin / WD-40 repeat protein family |
|  | 1438 | | 0.46 | 9.76 | 3.94E-02 | At2g27150 | aldehyde oxidase 3 (AO3) |
|  | 1439 | | -0.45 | 11.97 | 3.94E-02 | At2g45640 | expressed protein |
|  | 1440 | | 0.47 | 9.95 | 3.95E-02 | At3g07010 | polysaccharide lyase family 1 (pectate lyase) |
|  | 1441 | | 0.34 | 10.20 | 3.95E-02 | At2g25760 | protein kinase family |
|  | 1442 | | -0.71 | 11.04 | 3.96E-02 | At3g43110 | expressed protein |
|  | 1443 | | -0.41 | 9.88 | 3.96E-02 | At3g15352 | expressed protein |
|  | 1444 | | -0.51 | 10.77 | 3.98E-02 | At2g22040 | transducin / WD-40 repeat protein family |
|  | 1445 | | -0.42 | 9.00 | 3.98E-02 | At1g46912 | F-box protein-related |
|  | 1446 | | -0.56 | 9.84 | 3.98E-02 | At5g16740 | amino acid transporter family |
|  | 1447 | | -0.42 | 10.00 | 3.98E-02 | At1g67940 | ABC transporter family protein |
|  | 1448 | | 0.35 | 10.21 | 3.98E-02 | At1g63270 | ABC transporter family protein |
|  | 1449 | | -0.37 | 9.88 | 3.98E-02 | At1g05170 | galactosyltransferase family |
|  | 1450 | | 0.39 | 11.98 | 3.99E-02 | At1g64740 | tubulin alpha-1 chain (TUA1) |
|  | 1451 | | 0.45 | 12.64 | 3.99E-02 |  | Genomic sequence for Arabidopsis thaliana BAC F10A5 |
|  | 1452 | | -0.43 | 13.99 | 3.99E-02 | At1g49400 | ribosomal protein S17p family |
|  | 1453 | | 0.40 | 10.32 | 3.99E-02 | At5g63510 | transferase hexapeptide repeat family |
|  | 1454 | | -0.39 | 13.78 | 4.00E-02 | At5g42300 | ubiquitin family |
|  | 1455 | | 0.49 | 10.12 | 4.00E-02 | At3g25860 | dihydrolipoamide S-acetyltransferase |
|  | 1456 | | -0.80 | 10.41 | 4.01E-02 | At1g29450 | auxin-induced (indole-3-acetic acid induced) protein, putative |
|  | 1457 | | 0.42 | 9.49 | 4.01E-02 | At5g11550 | expressed protein |
|  | 1458 | | -0.85 | 10.87 | 4.01E-02 | At2g29360 | short-chain dehydrogenase/reductase family protein (tropinone reductase, putative) |
|  | 1459 | | 0.36 | 10.83 | 4.01E-02 | At4g02580 | predicted NADH dehydrogenase 24 kD subunit |
|  | 1460 | | -0.38 | 9.47 | 4.01E-02 | At2g14590 | Mutator-related transposase |
|  | 1461 | | -0.69 | 10.99 | 4.01E-02 | At5g22210 | expressed protein |
|  | 1462 | | 0.45 | 12.71 | 4.02E-02 | At2g19570 | cytidine deaminase -related |
|  | 1463 | | 0.39 | 10.45 | 4.02E-02 | At4g31180 | aspartyl-tRNA synthetase (aspartate--tRNA ligase), putative |
|  | 1464 | | -0.48 | 9.35 | 4.03E-02 | At5g15990 | hypothetical protein |
|  | 1465 | | -0.46 | 10.47 | 4.03E-02 | At4g18000 | hypothetical protein |
|  | 1466 | | 0.33 | 9.92 | 4.03E-02 | At3g21250 | ABC transporter family protein |
|  | 1467 | | -0.60 | 9.33 | 4.03E-02 | At3g09600 | myb family transcription factor |
|  | 1468 | | 0.35 | 12.96 | 4.04E-02 | At1g15740 | leucine rich repeat protein-related |
|  | 1469 | | 0.54 | 10.91 | 4.05E-02 | At5g03380 | heavy-metal-associated domain-containing protein |
|  | 1470 | | 0.43 | 12.79 | 4.05E-02 | At4g24370 | expressed protein |
|  | 1471 | | -0.43 | 10.46 | 4.05E-02 | At3g05570 | hypothetical protein |
|  | 1472 | | 0.36 | 9.86 | 4.05E-02 | At2g41160 | Arabidopsis thaliana chromosome II section 222 of 255 of the complete sequence. |
|  | 1473 | | -0.70 | 10.93 | 4.05E-02 | At3g62960 | glutaredoxin protein family |
|  | 1474 | | -0.51 | 8.86 | 4.05E-02 | At1g17345 | auxin-induced (indole-3-acetic acid induced) protein-related |
|  | 1475 | | -0.42 | 10.11 | 4.05E-02 | At2g40330 | Bet v I allergen family |
|  | 1476 | | -0.44 | 9.25 | 4.06E-02 | At2g11090 | hypothetical protein |
|  | 1477 | | -0.70 | 9.95 | 4.06E-02 | At2g43590 | glycosyl hydrolase family 19 (chitinase) |
|  | 1478 | | 0.39 | 10.27 | 4.06E-02 | At3g58670 | expressed protein |
|  | 1479 | | -0.34 | 9.51 | 4.07E-02 | At1g34904 | retrofit -related |
|  | 1480 | | 0.42 | 10.38 | 4.07E-02 | At3g09880 | B' regulatory subunit of PP2A (AtB'beta) |
|  | 1481 | | 0.42 | 10.87 | 4.08E-02 | At1g55360 | expressed protein |
|  | 1482 | | 0.42 | 11.78 | 4.08E-02 | At1g18540 | 60S ribosomal protein L6 (RPL6A) |
|  | 1483 | | 0.42 | 11.40 | 4.08E-02 | At1g48600 | Genomic sequence for Arabidopsis thaliana BAC T1N15 from chromosome I |
|  | 1484 | | 0.34 | 10.51 | 4.10E-02 | At5g04050 | maturase -related protein |
|  | 1485 | | 0.59 | 12.34 | 4.10E-02 | At5g05380 | expressed protein |
|  | 1486 | | -0.42 | 13.24 | 4.11E-02 | At3g13520 | arabinogalactan-protein (AGP12) |
|  | 1487 | | 0.47 | 12.98 | 4.11E-02 | At2g42800 | leucine rich repeat protein family |
|  | 1488 | | -0.53 | 10.10 | 4.11E-02 | At4g36530 | hydrolase, alpha/beta fold family |
|  | 1489 | | 0.67 | 10.64 | 4.12E-02 | At1g69810 | WRKY family transcription factor |
|  | 1490 | | -0.44 | 9.55 | 4.13E-02 | At4g14000 | expressed protein |
|  | 1491 | | -0.38 | 11.79 | 4.13E-02 | At1g56330 | GTP-binding protein SAR1B |
|  | 1492 | | 0.43 | 13.32 | 4.14E-02 | At2g21620 | auxin-regulated protein |
|  | 1493 | | 0.40 | 10.25 | 4.14E-02 | At3g50590 | transducin / WD-40 repeat protein family |
|  | 1494 | | -0.74 | 12.01 | 4.14E-02 | At2g01520 | major latex protein (MLP)-related |
|  | 1495 | | 0.34 | 10.53 | 4.14E-02 | At5g44820 | expressed protein |
|  | 1496 | | -0.32 | 13.38 | 4.14E-02 | At3g63530 | ESTs |
|  | 1497 | | 0.37 | 9.95 | 4.14E-02 | At4g28210 | expressed protein |
|  | 1498 | | 0.34 | 10.50 | 4.14E-02 | At5g43080 | cyclin, putative |
|  | 1499 | | -0.44 | 11.67 | 4.15E-02 | At1g29990 | hydrophilic protein -related |
|  | 1500 | | 0.66 | 10.67 | 4.15E-02 | At5g55980 | hypothetical protein |
|  | 1501 | | -0.40 | 11.12 | 4.16E-02 | At5g16650 | DnaJ protein family |
|  | 1502 | | 0.47 | 11.14 | 4.16E-02 | At5g11770 | NADH dehydrogenase (ubiquinone) |
|  | 1503 | | 0.51 | 9.78 | 4.16E-02 | At3g06370 | sodium proton exchanger, putative (NHX4) |
|  | 1504 | | 0.41 | 12.31 | 4.16E-02 | At2g43680 | SF16 protein {Helianthus annuus} -related |
|  | 1505 | | -0.40 | 11.74 | 4.16E-02 | At4g08280 | expressed protein |
|  | 1506 | | 0.46 | 9.57 | 4.18E-02 | At1g79280 | expressed protein |
|  | 1507 | | 0.37 | 9.93 | 4.18E-02 | At5g04460 | expressed protein |
|  | 1508 | | 0.40 | 11.28 | 4.18E-02 | At5g05250 | expressed protein |
|  | 1509 | | -0.50 | 13.25 | 4.19E-02 | At5g17170 | expressed protein |
|  | 1510 | | 0.50 | 15.11 | 4.19E-02 | At1g49150 | hypothetical protein |
|  | 1511 | | -0.46 | 11.51 | 4.20E-02 | At2g25700 | E3 ubiquitin ligase SCF complex subunit SKP1/ASK1 (At3), putative |
|  | 1512 | | -0.49 | 11.17 | 4.20E-02 | At5g52970 | thylakoid lumen 15.0-kDa protein |
|  | 1513 | | -0.46 | 9.66 | 4.22E-02 | At2g32680 | disease resistance protein family |
|  | 1514 | | -0.43 | 9.58 | 4.22E-02 | At5g65080 | Arabidopsis thaliana MADS box protein FCL1 (FCL1) mRNA, partial cds |
|  | 1515 | | 0.39 | 9.35 | 4.23E-02 | At3g12280 | Retinoblastoma protein |
|  | 1516 | | 0.41 | 10.87 | 4.24E-02 | At4g27520 | expressed protein |
|  | 1517 | | -0.84 | 12.25 | 4.24E-02 | At3g15580 | autophagy protein APG8H (AtAPG8i) |
|  | 1518 | | 0.43 | 11.61 | 4.25E-02 | At5g23060 | expressed protein |
|  | 1519 | | 0.65 | 11.09 | 4.25E-02 | At3g09370 | myb family transcription factor |
|  | 1520 | | -0.69 | 10.03 | 4.25E-02 | At2g29170 | short-chain dehydrogenase/reductase family protein (tropinone reductase, putative) |
|  | 1521 | | 0.63 | 10.14 | 4.25E-02 | At3g29670 | transferase family |
|  | 1522 | | -0.50 | 10.32 | 4.25E-02 | At1g14930 | Arabidopsis thaliana Unknown protein mRNA, complete cds |
|  | 1523 | | -0.72 | 10.38 | 4.26E-02 | At4g14640 | calmodulin 8 |
|  | 1524 | | 0.50 | 11.20 | 4.26E-02 | At3g21937 | Arabidopsis thaliana genomic DNA, chromosome 3, P1 clone:MZN24 |
|  | 1525 | | -0.30 | 12.25 | 4.26E-02 | At2g33450 | 50S ribosomal protein L28, chloroplast precursor (CL28) |
|  | 1526 | | 0.49 | 10.85 | 4.27E-02 | At1g07350 | transformer serine/arginine-rich ribonucleoprotein, putative |
|  | 1527 | | 0.30 | 9.72 | 4.28E-02 | At1g73380 | expressed protein |
|  | 1528 | | 0.48 | 10.28 | 4.28E-02 | At5g42100 | glycosyl hydrolase family 17 |
|  | 1529 | | 0.39 | 9.52 | 4.28E-02 | At1g09010 | glycoside hydrolase family 2 |
|  | 1530 | | -0.42 | 14.02 | 4.28E-02 | At1g02780 | 60S ribosomal protein L19 (RPL19A) |
|  | 1531 | | -0.30 | 11.70 | 4.28E-02 | At1g03210 | EST, Weakly similar to T37257 hypothetical protein R02D1.1 - Caenorhabditis elegans [C.elegans] |
|  | 1532 | | 0.55 | 10.99 | 4.28E-02 | At4g35790 | phospholipase D -related |
|  | 1533 | | -0.39 | 12.51 | 4.29E-02 | At5g28750 | Tha4 protein - like |
|  | 1534 | | 0.42 | 10.21 | 4.29E-02 | At2g41560 | potential calcium-transporting ATPase 4, plasma membrane-type (Ca2+ATPase, isoform 4) |
|  | 1535 | | 0.40 | 11.24 | 4.30E-02 | At4g38630 | 26S proteasome regulatory subunit S5A (RPN10) |
|  | 1536 | | 0.36 | 11.56 | 4.31E-02 | At4g19880 | expressed protein |
|  | 1537 | | -0.38 | 9.69 | 4.31E-02 | At5g02780 | In2-1 protein, putative |
|  | 1538 | | -0.44 | 11.13 | 4.31E-02 | At5g63030 | glutaredoxin, putative |
|  | 1539 | | 0.35 | 13.92 | 4.32E-02 | At2g28000 | RuBisCO subunit binding-protein alpha subunit/60 kDa chaperonin alpha subunit |
|  | 1540 | | -0.39 | 10.13 | 4.33E-02 |  | Arabidopsis thaliana genomic DNA, chromosome 5, P1 clone:MPO12 |
|  | 1541 | | 0.40 | 9.11 | 4.35E-02 | At5g16560 | myb family transcription factor |
|  | 1542 | | -0.54 | 9.60 | 4.35E-02 | At2g01540 | C2 domain-containing protein |
|  | 1543 | | -0.55 | 13.69 | 4.35E-02 | At4g05180 | oxygen-evolving complex protein 16, chloroplast precursor (OEC16) |
|  | 1544 | | -0.35 | 11.88 | 4.36E-02 | At2g22990 | serine carboxypeptidase -related |
|  | 1545 | | 0.52 | 10.98 | 4.36E-02 | At4g15460 | glycine-rich protein |
|  | 1546 | | 0.37 | 13.24 | 4.39E-02 | At2g19730 | 60S ribosomal protein L28 (RPL28A) |
|  | 1547 | | 0.36 | 10.06 | 4.39E-02 | At3g51620 | expressed protein |
|  | 1548 | | 0.39 | 10.08 | 4.39E-02 | At1g75800 | thaumatin family |
|  | 1549 | | -0.36 | 10.93 | 4.39E-02 | At3g18880 | ribosomal protein S17p family |
|  | 1550 | | 0.50 | 9.82 | 4.39E-02 | At1g09620 | leucyl-tRNA synthetase -related |
|  | 1551 | | -0.52 | 9.72 | 4.39E-02 | At5g03050 | expressed protein |
|  | 1552 | | -0.56 | 13.32 | 4.39E-02 | At3g53990 | expressed protein |
|  | 1553 | | -0.45 | 11.03 | 4.40E-02 | At4g03960 | expressed protein |
|  | 1554 | | -0.58 | 9.98 | 4.40E-02 | At5g67390 | expressed protein |
|  | 1555 | | 0.33 | 11.98 | 4.40E-02 | At3g51800 | nuclear DNA-binding protein G2p -related |
|  | 1556 | | 0.44 | 13.43 | 4.40E-02 | At1g74640 | expressed protein |
|  | 1557 | | 0.41 | 10.33 | 4.43E-02 | At4g36010 | thaumatin family |
|  | 1558 | | -0.34 | 10.37 | 4.44E-02 | At1g75100 | expressed protein |
|  | 1559 | | 0.43 | 11.45 | 4.44E-02 | At5g57740 | expressed protein |
|  | 1560 | | 0.52 | 9.59 | 4.44E-02 | At5g15680 | expressed protein |
|  | 1561 | | -0.31 | 12.26 | 4.44E-02 | At2g40765 | expressed protein |
|  | 1562 | | -0.41 | 8.88 | 4.44E-02 |  | Genomic sequence for Arabidopsis thaliana BAC T4O12 from chromosome I |
|  | 1563 | | -0.44 | 9.60 | 4.44E-02 | At1g30950 | unusual floral organ (UFO) (FBX1) |
|  | 1564 | | 0.45 | 10.66 | 4.45E-02 | At1g14000 | protein kinase -related |
|  | 1565 | | 0.40 | 12.40 | 4.45E-02 | At3g49290 | expressed protein |
|  | 1566 | | -0.48 | 9.98 | 4.45E-02 | At4g16840 | Arabidopsis thaliana mRNA for DYW10 protein, partial |
|  | 1567 | | -1.35 | 11.27 | 4.45E-02 | At1g53890 | expressed protein |
|  | 1568 | | 0.33 | 11.61 | 4.45E-02 | At4g29840 | threonine synthase, chloroplast |
|  | 1569 | | -0.52 | 10.88 | 4.45E-02 | At1g26350 | hypothetical protein |
|  | 1570 | | 0.59 | 9.70 | 4.45E-02 | At3g05940 | Arabidopsis thaliana chromosome III BAC F10A16 genomic sequence |
|  | 1571 | | 0.97 | 10.17 | 4.45E-02 | At2g28290 | SNF2 domain/helicase domain-containing protein |
|  | 1572 | | -0.40 | 13.90 | 4.45E-02 | At5g42300 | ubiquitin family |
|  | 1573 | | 0.43 | 9.72 | 4.46E-02 | At4g10710 | transcriptional regulator -related |
|  | 1574 | | -0.48 | 9.94 | 4.46E-02 | At1g04290 | thioesterase-related |
|  | 1575 | | -0.55 | 10.66 | 4.46E-02 | At2g19680 | copia-related retroelement pol polyprotein |
|  | 1576 | | -0.38 | 14.11 | 4.46E-02 | At1g09340 | RNA-binding protein -related |
|  | 1577 | | 0.57 | 10.47 | 4.46E-02 | At3g03050 | cellulose synthase family |
|  | 1578 | | 0.53 | 10.23 | 4.46E-02 | At2g32730 | 26S proteasome regulatory subunit (RPN2), putative |
|  | 1579 | | -0.34 | 12.81 | 4.46E-02 | At3g27360 | histone H3 |
|  | 1580 | | -0.37 | 11.31 | 4.46E-02 | At2g33290 | SET-domain transcriptional regulator family |
|  | 1581 | | 0.49 | 10.69 | 4.46E-02 | At3g23940 | dihydroxyacid dehydratase -related |
|  | 1582 | | 0.47 | 10.24 | 4.46E-02 | At1g66560 | WRKY family transcription factor |
|  | 1583 | | 0.36 | 11.35 | 4.46E-02 | At3g05290 | mitochondrial carrier protein family |
|  | 1584 | | -0.69 | 12.02 | 4.46E-02 | At1g51110 | plastid-lipid associated protein PAP/fibrillin family |
|  | 1585 | | -0.41 | 11.32 | 4.46E-02 | At1g25260 | expressed protein |
|  | 1586 | | 0.42 | 9.17 | 4.46E-02 | At2g27060 | leucine-rich repeat transmembrane protein kinase, putative |
|  | 1587 | | 0.43 | 9.18 | 4.46E-02 | At3g50370 | expressed protein |
|  | 1588 | | 0.85 | 10.82 | 4.46E-02 | At3g51550 | protein kinase family |
|  | 1589 | | -0.35 | 11.05 | 4.46E-02 | At5g14580 | polynucleotide phosphorylase |
|  | 1590 | | -0.35 | 8.74 | 4.46E-02 | At2g02210 | Ulp1 protease family |
|  | 1591 | | 0.62 | 11.03 | 4.46E-02 | At4g37360 | cytochrome P450 family |
|  | 1592 | | 0.37 | 15.14 | 4.46E-02 | At4g11680 | zinc finger (C3HC4-type RING finger) protein family |
|  | 1593 | | -0.37 | 14.70 | 4.46E-02 | At3g04400 | 60S ribosomal protein L23 (RPL23C) |
|  | 1594 | | 0.38 | 11.03 | 4.46E-02 | At3g20940 | cytochrome P450 family |
|  | 1595 | | 1.22 | 9.89 | 4.46E-02 | At2g29500 | small heat shock protein -related |
|  | 1596 | | 0.41 | 9.68 | 4.47E-02 | At1g06220 | elongation factor Tu family protein |
|  | 1597 | | 0.60 | 11.79 | 4.48E-02 | At5g49910 | heat shock protein cpHsc70-2 (hsc70-7) |
|  | 1598 | | -0.75 | 10.89 | 4.48E-02 |  | Arabidopsis thaliana chromosome I BAC F20D23 genomic sequence |
|  | 1599 | | 0.40 | 9.21 | 4.49E-02 | At3g05420 | acyl CoA binding protein (ACBP) family |
|  | 1600 | | -0.50 | 9.63 | 4.49E-02 | At1g47580 | hypothetical protein |
|  | 1601 | | -0.63 | 11.25 | 4.49E-02 | At2g03310 | expressed protein |
|  | 1602 | | 0.38 | 10.10 | 4.49E-02 | At5g45620 | 26S proteasome regulatory subunit (RPN9), putative |
|  | 1603 | | 0.60 | 9.68 | 4.50E-02 | At3g52140 | tetratricopeptide repeat (TPR)-containing protein |
|  | 1604 | | 0.52 | 13.64 | 4.50E-02 | At1g53240 | malate dehydrogenase [NAD], mitochondrial, putative |
|  | 1605 | | 0.36 | 10.09 | 4.50E-02 | At2g41960 | expressed protein |
|  | 1606 | | 0.51 | 11.47 | 4.50E-02 | At4g31340 | expressed protein |
|  | 1607 | | -0.43 | 8.92 | 4.50E-02 | At4g18450 | ethylene response factor, putative |
|  | 1608 | | 0.57 | 10.11 | 4.50E-02 | At5g16390 | biotin carboxyl carrier protein 1 (BCCP1) |
|  | 1609 | | -0.37 | 11.99 | 4.51E-02 | At2g35260 | expressed protein |
|  | 1610 | | -0.40 | 14.36 | 4.52E-02 | At2g15890 | expressed protein |
|  | 1611 | | 0.49 | 10.56 | 4.52E-02 | At5g35210 | PHD finger transcription factor, putative |
|  | 1612 | | -0.51 | 11.03 | 4.53E-02 |  | Genomic sequence for Arabidopsis thaliana BAC T10O22 from chromosome I |
|  | 1613 | | -0.54 | 10.52 | 4.53E-02 | At1g19390 | Genomic sequence for Arabidopsis thaliana BAC F18O14 from chromosome I |
|  | 1614 | | 0.32 | 13.88 | 4.53E-02 | At2g37620 | actin 3 |
|  | 1615 | | -0.51 | 11.40 | 4.54E-02 | At3g14430 | expressed protein |
|  | 1616 | | 0.58 | 10.35 | 4.55E-02 | At4g09110 | hypothetical protein |
|  | 1617 | | 0.67 | 11.42 | 4.55E-02 | At5g14800 | pyrroline-5-carboxylate reductase |
|  | 1618 | | -0.47 | 11.48 | 4.56E-02 | At3g05550 | hypothetical protein |
|  | 1619 | | 0.71 | 11.89 | 4.56E-02 | At3g59440 | calcium-binding protein, putative |
|  | 1620 | | 0.48 | 9.22 | 4.56E-02 | At2g37260 | WRKY family transcription factor |
|  | 1621 | | 0.40 | 10.36 | 4.56E-02 | At3g05060 | SAR DNA-binding protein, putative |
|  | 1622 | | 0.72 | 10.49 | 4.56E-02 | At1g23790 | Arabidopsis thaliana chromosome 1 BAC F5O8 sequence |
|  | 1623 | | -0.39 | 10.46 | 4.56E-02 | At3g08920 | rhodanese-like domain protein |
|  | 1624 | | -0.32 | 9.45 | 4.56E-02 | At4g11750 | Kelch repeat containing F-box protein family |
|  | 1625 | | 0.52 | 10.46 | 4.57E-02 | At3g20110 | cytochrome P450 family |
|  | 1626 | | 0.43 | 9.96 | 4.58E-02 | At1g16270 | protein kinase family |
|  | 1627 | | 0.47 | 10.23 | 4.58E-02 | At5g64660 | expressed protein |
|  | 1628 | | -0.44 | 8.78 | 4.58E-02 | At3g32030 | Arabidopsis thaliana genomic DNA, chromosome 3, BAC clone: T22B15 |
|  | 1629 | | 0.41 | 12.72 | 4.58E-02 | At1g22900 | disease resistance response protein-related |
|  | 1630 | | -0.56 | 9.01 | 4.58E-02 | At1g63700 | ESTs |
|  | 1631 | | 0.29 | 8.88 | 4.58E-02 | At4g10600 | Arabidopsis thaliana BAC F3H7 |
|  | 1632 | | -0.55 | 10.80 | 4.59E-02 | At1g60000 | 29 kDa ribonucleoprotein, chloroplast (RNA-binding protein cp29), putative |
|  | 1633 | | 0.44 | 10.40 | 4.59E-02 | At4g17310 | Arabidopsis thaliana DNA chromosome 4, contig fragment No. 46 |
|  | 1634 | | 0.40 | 11.08 | 4.62E-02 | At1g56110 | nucleolar protein Nop56, putative |
|  | 1635 | | 0.62 | 10.58 | 4.63E-02 | At3g45010 | serine carboxypeptidase III, putative |
|  | 1636 | | -0.51 | 9.91 | 4.63E-02 | At1g08890 | sugar transporter family |
|  | 1637 | | 0.32 | 11.24 | 4.63E-02 | At1g77440 | 20S proteasome beta subunit C (PBC2) |
|  | 1638 | | -0.89 | 10.35 | 4.63E-02 | At1g66100 | thionin, putative |
|  | 1639 | | -0.43 | 11.42 | 4.63E-02 | At1g27435 | expressed protein |
|  | 1640 | | 0.38 | 9.31 | 4.63E-02 | At3g48520 | cytochrome P450 family |
|  | 1641 | | -0.36 | 11.56 | 4.63E-02 | At2g17380 | clathrin assembly protein AP19, small subunit |
|  | 1642 | | 0.59 | 11.06 | 4.64E-02 | At2g40840 | glycosyl hydrolase family 77 (4-alpha-glucanotransferase) |
|  | 1643 | | -0.56 | 11.91 | 4.64E-02 |  | Arabidopsis thaliana genomic DNA, chromosome 3, P1 clone: MQP17 |
|  | 1644 | | 0.57 | 10.21 | 4.64E-02 | At3g15470 | expressed protein |
|  | 1645 | | -0.41 | 13.59 | 4.64E-02 | At5g42300 | ubiquitin family |
|  | 1646 | | 0.33 | 9.62 | 4.64E-02 | At3g44270 | hypothetical protein |
|  | 1647 | | 0.48 | 9.71 | 4.64E-02 | At5g61130 | glycosyl hydrolase family 17 |
|  | 1648 | | 0.57 | 11.64 | 4.64E-02 | At5g49910 | heat shock protein cpHsc70-2 (hsc70-7) |
|  | 1649 | | 0.69 | 9.38 | 4.66E-02 | At3g56980 | bHLH protein family |
|  | 1650 | | 0.50 | 8.97 | 4.66E-02 | At3g50870 | GATA zinc finger protein |
|  | 1651 | | 0.92 | 12.16 | 4.67E-02 | At2g41730 | expressed protein |
|  | 1652 | | 0.47 | 8.88 | 4.67E-02 | At3g24040 | expressed protein |
|  | 1653 | | -0.61 | 10.45 | 4.67E-02 | At4g25060 | hypothetical protein |
|  | 1654 | | 0.59 | 10.70 | 4.67E-02 | At5g59050 | expressed protein |
|  | 1655 | | -0.58 | 11.60 | 4.67E-02 | At3g22920 | cyclophilin -related |
|  | 1656 | | -0.43 | 9.78 | 4.68E-02 | At5g50760 | auxin-induced (indole-3-acetic acid induced) protein family |
|  | 1657 | | 0.50 | 9.83 | 4.68E-02 | At4g00630 | Arabidopsis thaliana DNA chromosome 4, contig fragment No. 2 |
|  | 1658 | | -0.51 | 9.34 | 4.68E-02 | At3g09050 | expressed protein |
|  | 1659 | | 0.30 | 11.99 | 4.68E-02 | At3g18130 | guanine nucleotide-binding protein / activated protein kinase C receptor RACK1 |
|  | 1660 | | -0.53 | 10.68 | 4.68E-02 | At2g44920 | thylakoid lumen pentapeptide repeat family protein |
|  | 1661 | | 0.54 | 14.76 | 4.69E-02 | At5g22260 | expressed protein |
|  | 1662 | | -0.42 | 9.55 | 4.69E-02 | At2g28330 | expressed protein |
|  | 1663 | | 0.35 | 15.63 | 4.71E-02 | At5g02490 | heat shock protein hsc70-2 (hsc70.2) (hsp70-2) |
|  | 1664 | | -0.37 | 13.06 | 4.71E-02 | At3g10520 | class 2 non-symbiotic hemoglobin |
|  | 1665 | | 0.51 | 10.11 | 4.71E-02 | At1g15810 | chloroplast 30S ribosomal protein S15, putative |
|  | 1666 | | -0.36 | 11.54 | 4.71E-02 | At3g45600 | senescence-associated protein family |
|  | 1667 | | -0.35 | 10.07 | 4.73E-02 | At1g29060 | Arabidopsis thaliana chromosome I BAC F28N24 genomic sequence |
|  | 1668 | | 0.47 | 10.29 | 4.73E-02 | At1g75490 | DRE binding transcription factor, putative |
|  | 1669 | | 0.46 | 9.87 | 4.73E-02 | At5g59710 | VIP2 protein |
|  | 1670 | | 0.59 | 9.86 | 4.73E-02 | At3g16070 | hypothetical protein |
|  | 1671 | | -0.53 | 12.36 | 4.74E-02 | At3g33073 | hypothetical protein |
|  | 1672 | | 0.45 | 13.47 | 4.74E-02 | At2g36400 | transcription activator (GRL3) |
|  | 1673 | | 0.50 | 10.09 | 4.75E-02 | At3g48500 | expressed protein |
|  | 1674 | | -0.67 | 9.73 | 4.75E-02 | At1g66330 | expressed protein |
|  | 1675 | | -0.97 | 9.73 | 4.75E-02 | At3g26210 | cytochrome P450 71B23 |
|  | 1676 | | 0.37 | 9.96 | 4.75E-02 | At5g45510 | leucine rich repeat protein family |
|  | 1677 | | 0.33 | 10.07 | 4.75E-02 | At5g03290 | isocitrate dehydrogenase [NAD], putative |
|  | 1678 | | -0.59 | 11.98 | 4.75E-02 | At1g22780 | 40S ribosomal protein S18 (RPS18A) |
|  | 1679 | | -0.70 | 9.58 | 4.75E-02 | At1g49200 | RING-H2 finger protein RHA3a -related |
|  | 1680 | | 0.32 | 9.85 | 4.75E-02 | At1g09000 | protein kinase, putative |
|  | 1681 | | -0.37 | 10.83 | 4.75E-02 | At3g16100 | Ras family GTP-binding protein |
|  | 1682 | | -0.49 | 10.06 | 4.75E-02 | At2g46140 | desiccation related protein -related |
|  | 1683 | | 0.43 | 11.13 | 4.75E-02 | At5g17570 | expressed protein |
|  | 1684 | | 0.33 | 10.27 | 4.76E-02 | At1g60070 | gamma-adaptin, putative |
|  | 1685 | | 0.30 | 10.19 | 4.76E-02 | At1g12140 | flavin-containing monooxygenase (FMO) family |
|  | 1686 | | -0.53 | 10.16 | 4.76E-02 | At3g53730 | histone H4 |
|  | 1687 | | -0.42 | 12.48 | 4.76E-02 | At3g55170 | 60S ribosomal protein L35 (RPL35C) |
|  | 1688 | | -0.32 | 11.22 | 4.76E-02 | At5g54660 | expressed protein |
|  | 1689 | | 0.33 | 10.16 | 4.77E-02 | At3g02530 | chaperonin, putative |
|  | 1690 | | 0.38 | 10.85 | 4.77E-02 | At2g01290 | ribose 5-phosphate isomerase -related |
|  | 1691 | | 0.52 | 9.85 | 4.77E-02 | At4g37150 | hydrolase, alpha/beta fold family |
|  | 1692 | | -0.33 | 10.92 | 4.78E-02 | At2g21190 | ER lumen protein retaining receptor -related |
|  | 1693 | | -0.73 | 10.91 | 4.79E-02 | At3g46080 | zinc finger -related protein |
|  | 1694 | | 0.51 | 13.27 | 4.79E-02 | At5g16930 | expressed protein |
|  | 1695 | | 0.39 | 12.39 | 4.79E-02 | At5g47210 | nuclear RNA binding protein, putative |
|  | 1696 | | 0.37 | 13.78 | 4.79E-02 | At4g17800 | expressed protein |
|  | 1697 | | 0.34 | 9.63 | 4.80E-02 | At4g16810 | hypothetical protein |
|  | 1698 | | -0.46 | 9.67 | 4.81E-02 | At5g59560 | sensitivity to red light reduced protein (SRR1) |
|  | 1699 | | -0.45 | 13.30 | 4.81E-02 | At1g18710 | myb-related transcription factor mixta, putative |
|  | 1700 | | 0.43 | 9.74 | 4.82E-02 | At5g06300 | lysine decarboxylase-related protein |
|  | 1701 | | 0.36 | 13.76 | 4.82E-02 | At5g62700 | tubulin beta-2/beta-3 chain (TUB3) |
|  | 1702 | | -0.48 | 12.04 | 4.82E-02 | At1g64680 | expressed protein |
|  | 1703 | | 0.70 | 10.28 | 4.82E-02 | At1g61930 | expressed protein |
|  | 1704 | | 0.29 | 12.68 | 4.82E-02 | At3g53750 | actin (ACT3) |
|  | 1705 | | -0.67 | 14.50 | 4.82E-02 | At1g13930 | expressed protein |
|  | 1706 | | -0.78 | 9.60 | 4.83E-02 | At1g62975 | basic helix-loop-helix transcription factor (bHLH125) |
|  | 1707 | | 0.43 | 9.75 | 4.83E-02 | At1g19835 | expressed protein |
|  | 1708 | | 0.73 | 10.57 | 4.84E-02 | At4g39400 | brassinosteroid insensitive 1 (BRI1) |
|  | 1709 | | 0.35 | 10.46 | 4.85E-02 | At4g13780 | methionyl-tRNA synthetase - like protein |
|  | 1710 | | 0.31 | 10.44 | 4.85E-02 | At5g56080 | nicotianamine synthase, putative |
|  | 1711 | | 0.47 | 10.07 | 4.86E-02 | At5g02410 | expressed protein |
|  | 1712 | | 0.30 | 11.02 | 4.87E-02 | At4g37870 | phosphoenolpyruvate carboxykinase (ATP) -related protein |
|  | 1713 | | -0.99 | 9.68 | 4.87E-02 | At3g16360 | two-component phosphorelay mediator -related |
|  | 1714 | | 0.41 | 11.27 | 4.87E-02 | At4g22530 | expressed protein |
|  | 1715 | | -0.34 | 10.94 | 4.89E-02 | At1g28490 | syntaxin SYP6 family |
|  | 1716 | | 0.66 | 9.75 | 4.90E-02 | At1g06950 | chloroplast inner envelope protein -related |
|  | 1717 | | 0.40 | 11.36 | 4.91E-02 | At3g63390 | expressed protein |
|  | 1718 | | -0.64 | 9.20 | 4.91E-02 | At1g01750 | actin depolymerizing factor -related |
|  | 1719 | | -0.58 | 9.17 | 4.91E-02 | At1g54660 | Sequence of BAC T22H22 from Arabidopsis thaliana chromosome 1 |
|  | 1720 | | 0.38 | 12.36 | 4.92E-02 | At3g60480 | expressed protein |
|  | 1721 | | -0.83 | 11.72 | 4.92E-02 | At1g31170 | expressed protein |
|  | 1722 | | -0.32 | 10.91 | 4.93E-02 | At4g14010 | expressed protein |
|  | 1723 | | 0.51 | 9.79 | 4.93E-02 | At3g27170 | CLC-b chloride channel protein |
|  | 1724 | | 0.39 | 8.60 | 4.93E-02 | At2g26130 | hypothetical protein |
|  | 1725 | | -0.38 | 8.98 | 4.94E-02 | At1g70760 | expressed protein |
|  | 1726 | | -0.47 | 9.18 | 4.95E-02 | At1g73440 | expressed protein |
|  | 1727 | | -0.44 | 14.91 | 4.95E-02 | At5g37780 | calmodulin 4 |
|  | 1728 | | -0.34 | 10.15 | 4.96E-02 | At1g75980 | Genomic sequence for Arabidopsis thaliana BAC T4O12 from chromosome I |
|  | 1729 | | -0.39 | 10.04 | 4.96E-02 |  | EST |
|  | 1730 | | 0.57 | 10.48 | 4.97E-02 | At3g06690 | hypothetical protein |
|  | 1731 | | -0.31 | 11.98 | 4.97E-02 | At1g73655 | immunophilin / FKBP-type peptidyl-prolyl cis-trans isomerase, putative |
|  | 1732 | | -0.50 | 13.16 | 4.97E-02 | At1g75350 | chloroplast 50S ribosomal protein L31 -related |
|  | 1733 | | 0.51 | 9.85 | 4.98E-02 | At1g08600 | SNF2domain/helicase domain-containing protein |
|  | 1734 | | -0.36 | 10.69 | 4.98E-02 | At5g04850 | expressed protein |
|  | 1735 | | -0.34 | 9.26 | 4.98E-02 | At2g02310 | F-box protein (SKP1 interacting partner 3-related) |
|  | 1736 | | 0.40 | 9.16 | 4.98E-02 | At2g33840 | EST, Weakly similar to SYY_HUMAN TYROSYL-TRNA SYNTHETASE [H.sapiens] |
|  | 1737 | | -0.56 | 8.91 | 4.98E-02 | At2g17740 | CHP-rich zinc finger protein, putative |
|  | 1738 | | -0.62 | 10.23 | 4.98E-02 | At5g64120 | A.thaliana mRNA for peroxidase ATP15a, clone EST 151A15T7 |
|  | 1739 | | 0.52 | 11.02 | 4.99E-02 | At2g26150 | heat shock transcription factor family |
|  | 1740 | | 0.54 | 11.98 | 4.99E-02 | At5g53050 | hydrolase, alpha/beta fold family |
|  | 1741 | | -0.40 | 10.27 | 4.99E-02 | At1g26340 | cytochrome b5, putative |
|  | 1742 | | -0.42 | 9.86 | 5.00E-02 | At5g62360 | pectinesterase family |
|  | 1743 | | -0.35 | 13.79 | 5.00E-02 | At5g42300 | ubiquitin family |
|  | 1744 | | -0.39 | 10.94 | 5.00E-02 | At5g11280 | expressed protein |
