# Supplementary material for: Identification of a cis-regulatory element by transient analysis of co-ordinately regulated genes
Source: Plant Methods. 2008 Jul 7;4:17. doi: 10.1186/1746-4811-4-17 (PMC2491621; doi:10.1186/1746-4811-4-17)
Supplement: Additional file 2 — Oligonucleotide primer pairs used to PCR amplify 1 kb of Arabidopsis promoter sequence. [file 1746-4811-4-17-S2.doc]

| Gene ID | 5' oligonucleotide primer (contains XmaI) | 3' oligonucleotide primer (contains NotI) |
| --- | --- | --- |
| At5g42800 | ACTTTGTCTCCCCGGGTGGAGGAGAGTCAAAATTAA | CTCTTTCTGAGCGGCCGCTTTTGTGGTTATATGATAGA |
| At5g17220 | AGTCAGTGTCCCCGGGGGTTGTTTCTGTTGTAGAAA | ATATAGTTTCGCGGCCGCTCTATAAGTTTACAATAACT |
| At1g56650 | AAGTCCTCAACCCGGGCGACTAAAAATGCTTTTCTT | TTTGGACGAAGCGGCCGCGGAACAAAGATAGATACGTA |
| At4g22880 | AACGGCGGAGCCCGGGCGAGGACTTGTCAAACTAAA | TCTTTCAACCGCGGCCGCCTTCTTTAGTCTTCTGTTTA |
| At5g13930 | TACGCCTCGCCCCGGGATCATATTTAACCGTCAATA | AGCACCAGCCGCGGCCGCTATAGTATACACCAACTTGG |
| At5g59310 | ATTTTGTAATCCCGGGAACTATGAGACATATATGAG | CCTCAAAGCGGCGGCCGCTGTGTTTGCTCTTCTCTTTT |
| At1g66380 | TTATGTTATACCCGGGTTGTTTCACCAAAATCAAAT | TTTGGACGAAGCGGCCGCGGACCAAAGATAGATATCTA |
| At5g24770 | GTTTTCGACGCCCGGGCGACGGGTTTTGGCGACGAA | AAGTGAGAGGGCGGCCGCGTTTTTTATGGTATGGTTTA |
| At1g03940 | GGGGCTCTTTCCCGGGGATCTTGTTGTTTCTGTGGG | TTGAAGATGAGCGGCCGCGGTGTGATTAAGTTTTGAGG |
| At4g34710 | AACCTAAAAACCCGGGAAGGAGAAGAAGCAGAGACG | GCAAGCTAAAGCGGCCGCCTTTATCTTCACCCTCTTAT |
| At5g05270 | TTAAAAACAACCCGGGCATGGAGATTTGGGCTTATA | GACCATCTCTGCGGCCGCTTTCTAATTTATATTATTTT |
| At2g37040 | AACGGCGTGTCCCGGGACTCGCCTCCTATTGGGAAT | TGCCCCGTTAGCGGCCGCTTAGACTTTTGATCTTAGTT |
| At1g02950 | TATGTTGATGCCCGGGTCATGTCAAGACACTTGGAG | CATCTGTAAGGCGGCCGCATATACGATATAGGCATGTA |
| At3g51240 | TTGGCTGATGCCCGGGTCTCCATTTGAATTTCGAGT | CAAAGTTCCTGCGGCCGCTGTAATTACGAAGACAAAAG |
| At5g56000 | ACTATTTTTTCCCGGGAAAATACAAAACTTTATTAA | GGTCTCTGCGGCGGCCGCTATCGTTGATCGGGAAAAAA |
| At5g09530 | GGAAACTGACCCCGGGTTGATTCGTTTGTGGAATCA | CTTCTTCATTGCGGCCGCGTTTGAGCTTGCTTGAGAAC |
| At4g09820 | CATTTATTTTCCCGGGAAAAAAGCCAAGAAACATTC | AATACTTGATGCGGCCGCCGTTCCCGGAGATACGAAAA |
| At5g07990 | TTCTGAAAGTCCCGGGATATGGTACTAGATATTCTC | GAGAAATAGAGCGGCCGCAGTGTTGGGTTTGAATGGTA |
| At4g35800 | AGAATCCTATCCCGGGATCTTAATCAAAAGAACAAA | CGGAAACCTCGCGGCCGCGGCGGCTAAGCTCCGATCAA |
| At1g30835 | CTCCTCGGAGCCCGGGAGTTTTTTCTGCGATTCGAC | ATTCATCCTTGCGGCCGCCGGATCTGGAAGGTGATGGA |
| At3g49270 | CATTCCGCAACCCGGGTTGTTCTCACTATAAAGAGA | CTGCATCTTTGCGGCCGCGTTTTTTTTTCCGAGAAAAA |
| At1g53540 | ACATCTCTACCCCGGGTCCAATCTCTGTTTTTGCAG | GCTTGGAATTGCGGCCGCCGTTTCACTTCCTCTTGTGA |
| At5g22860 | GCAAATGCTACCCGGGTGATGTATCAATCATATGGA | TGTATATGGAGCGGCCGCTTTAGAATGAGAGTGTTTAT |
| At3g53230 | GGTTTTGTTTCCCGGGTTTCAGGTGTGGAGTTTGTA | TTCAGCTTGAGCGGCCGCGTTTGATAATCGAGTAGAGA |
| At5g56010 | TTGTTAAATTCCCGGGTTAGCTAAACTAATCAATAA | GGTTTCTGCGGCGGCCGCTCTCGTTGATCGGAAAAAAA |
| At4g14090 | CACTCTATCCCCCGGGTCCATAAAACTTGAAGAAAC | ATTGACGGAAGCGGCCGCTTTAGAATGTTTTGAAGATG |
| At5g13170 | AACATGCATGCCCGGGAACGTTTTCTTTTTGTACAA | ATTGATCATGGCGGCCGCTTTCTATAGCAATTGAGAAA |
| At3g02480 | AGGGAATCTACCCGGGTTTTTCTTCCTAATAACTAT | GTTTTGCTTGGCGGCCGCTTTCTCTCGTTCTGGTTTTG |
| At3g08860 | CTTATTCCGTCCCGGGTTGCTTTTGTTATCAACTCA | CGCCGTTAACGCGGCCGCTTTTTCACCGACGTTAAAAC |
| At5g24560 | GCTTTATGTTCCCGGGCGTTGACGGCTCAGTTTTCT | TAAATCAAGAGCGGCCGCCTCCATTGTAATAAGTTGAA |
| At5g24780 | TAAAGCTGTACCCGGGTATAAGAATTCATAGTAGTT | AAGTGAGAGGGCGGCCGCATTTTTTTGTATGGTTTATT |
| At5g48180 | CGAATCGTTCCCCGGGTCAATTTGGTTTGTGATGAG | GTTCTCCACCGCGGCCGCTTTTTTCGTTACTCACCAGA |
| At5g59320 | AAAAATTTATCCCGGGTTATATTTAAGAGTTTAAAA | CCTCAAAGCGGCGGCCGCTGTGTTTGAACTTCTTTTTG |
| At1g09500 | TGAGATGATACCCGGGGTGTGTGTGGAGAAAGTGAA | CTTTCCTCCAGCGGCCGCCTCTTTTCACTTTATTTGGC |
| At5g48850 | CAAGTCGGAACCCGGGACAAGACCATCGAAAGATCT | CTTCAAGCTTGCGGCCGCCTTTTTTTCCTCTGTTTTTC |
| At4g33150 | CCATACTATTCCCGGGACACACTGATTTTGAAGTCA | ATGGCCATTTGCGGCCGCCTGATGAGTTTGATCATGCA |
| At5g48570 | GAGGCTGAGACCCGGGAAGAAGTCATGATATTCATA | GTCGAAATCGGCGGCCGCTTTCTGATCAAATTTAAGAG |
| At2g43150 | TATTAAAATCCCCGGGTTATGCCACAAATCAAAGTC | CCATGCCGGAGCGGCCGCTCTTCACCGGATAATGTTCA |
| At3g21720 | TTCGTAAACCCCCGGGAAAAACGCTTCCGAGAAAAA | AGAGAAAGATGCGGCCGCGGCTTTAACTTTTATAAATT |
| At2g29500 | AACACCAGGACCCGGGCGAGAGTGTTATTGGCAGTG | ACTTGGAATCGCGGCCGCTTTAAAGAATTTACTTTTTT |
| At2g41240 | GTTTTTAGAGCCCGGGATTTAGATCTTTGGATTTAA | AGGGACAAGTGCGGCCGCTTTGAGTTTTAGATAGTTAC |
| At1g59860 | TTACTATTAGCCCGGGTCCAAAATTTCTAGTTTTCG | GCTTGGAATCGCGGCCGCTTTCTCGGTTGATTTCGAAA |
| At1g07400 | CACATCTTAACCCGGGTGATGGTGTCAGGAATGGTT | GCTTGGAATAGCGGCCGCTCTCGATTTGATTTCGAAAG |
| At1g74310 | TTGAATGCCACCCGGGAAGATAAATCTCTCTGTACA | GAATTTCTCTGCGGCCGCCTTCGATTAGCTTTTGTAAT |
| At3g22120 | CCTTATAAAACCCGGGTGGACCTACCTCGCAAGGAA | TTGAGAGCGAGCGGCCGCTTTTTGGAATGAGGGTTTGG |
| At3g54580 | CAAAAATCAACCCGGGGACTAAGTGACTAATAAAGA | ATGGGTTGAAGCGGCCGCCATAGAAGAAGATCTCATTG |
| At5g06640 | TTAATAGTGACCCGGGATATACAGTATATAACACTG | TATGTTATACGCGGCCGCGAATACCATTTATATAGTCT |
| At3g28550 | GTATAATCTTCCCGGGCGTTTTTACTAGGTTTATTA | ATGGGCAGAAGCGGCCGCTTTAGAAGAAGATATCATTG |

Additional file 2. Primer pairs used to PCR amplify 1kb of Arabidopsis promoter sequence. Restriction enzyme sites are incorporated in the 5’ (XmaI) and 3’ (NotI) primers to facilitate directional cloning.
