# Supplementary material for: Identification of a cis-regulatory element by transient analysis of co-ordinately regulated genes
Source: Plant Methods. 2008 Jul 7;4:17. doi: 10.1186/1746-4811-4-17 (PMC2491621; doi:10.1186/1746-4811-4-17)
Supplement: Additional file 3 — Oligonucleotide primer pairs used to delete or mutate the PCE in the At5g17220 and At4g14090 promoters. [file 1746-4811-4-17-S3.doc]

Additional file 3. Oligonucleotide primer pairs used to delete or mutate bases in the PCE in the At5g17220 and At4g14090 promoters

| Oligonucleotide Primer | Sequence |
| --- | --- |
| At5g17220 deletion forward | TTCTACCCAACCCTCACAACAACC |
| Atg517220 deletion reverse | TTGTTCCTCTAACGGTGGATATGTATCA |
| Atg517220 mutation forward | ATATATTCTACCCAACCCTCACAACAACC |
| Atg517220 mutation reverse | GATGATTGTTCCTCTAACGGTGGAT |
| At4g14090 deletion forward | TTTCAGTCTGTTTTTGATCAGTTCTCAA |
| At4g 14090 deletion reverse | CATGGTGGTTGGTTGCGGT |
| At4g 14090 mutation forward | ATTAAGATTGAGAACTGATCAAAAACAG |
| At4g 14090 mutation forward | TATACCGCAACCAACCACCATGGTA |
